# Supplementary material for: Ambient-pressure alkoxycarbonylation for sustainable synthesis of ester
Source: Nat Commun. 2024 Sep 7;15:7837. doi: 10.1038/s41467-024-52163-2 (PMC11380687; doi:10.1038/s41467-024-52163-2)
Supplement: Supplementary file 1 — Supplementary Information [file 41467_2024_52163_MOESM1_ESM.pdf]

# Ambient-pressure Alkoxycarbonylation for Sustainable Synthesis of Ester

Bin Zhang[#], Haiyang Yuan[#], Ye Liu[#], Zijie Deng, Mark Douthwaite*, Nicholas F. Dummer, Richard J. Lewis, Xingwu Liu, Sen Luan, Minghua Dong, Tianjiao Wang, Zhijuan Zhao, Qingling Xu*, Huizhen Liu*, Buxing Han, Graham J. Hutchings

**Supplementary materials:**

Supplementary Table 1–22

Supplementary Figure 1–21

Supplementary Note 1–7

## Table of Content

Supplementary Table 1 Representative recent cases of alkoxycarbonylation of alkenes via heterogenous catalysis (Entry 1-7) and homogeneous catalysis (Entry 8-15)....... 4
Supplementary Note 1: CO utilization efficiency and TOF calculations in Supplementary Table 1. ....... 5
Supplementary Table 2 Screening of effective heterogeneous catalysts for styrene methoxylcarbonylation. ....... 5
Supplementary Table 3. The best-fitted EXAFS results of $Ru/NbO_x^a$....... 5
Supplementary Table 4 Optimization of temperature for styrene carbonylation. ....... 6
Supplementary Table 5 Pressure-dependent evolutions of styrene carbonylation. ....... 7
Supplementary Table 6 Effects of catalyst weight loadings on styrene carbonylation. ....... 7
Supplementary Table 7 Effects of styrene concentration on styrene carbonylation....... 7
Supplementary Table 8 Effects of solvent for styrene carbonylation. ....... 7
Supplementary Table 9 Effects of water for styrene carbonylation....... 8
Supplementary Table 10 Effects of reaction atmosphere for styrene carbonylation....... 8
Supplementary Table 11 Ru dispersion, CO pulse chemisorption data and ICP-OES data of $Ru_1/NbO_x$ and 2 wt.% $Ru/NbO_x$ catalysts....... 8
Supplementary Table 12 Effects of fully dispersed Ru and size-dependent Ru clusters on styrene carbonylation....... 8
Supplementary Table 13 The deconvolution of CO-adsorption FTIR spectra on 2 wt.% $Ru/NbO_x$ catalysts (Figure 2h)....... 9
Supplementary Note 2 Calculation of the average factor of $n_{Ru}/n_{CO}$....... 9
Supplementary Table 14 The reported band assignment of various Ru carbonyl species absorbed on oxide in CO-probed infrared spectra. 9
Supplementary Table 15 Effects of active Ru components on styrene carbonylation. ....... 9
Supplementary Table 16 Substrate scopes of substituted vinyl arenes. ....... 10
Supplementary Table 17 Dependence of side chains of aryl olefins on their carbonylation performance. ....... 10
Supplementary Table 18 Dependence between terminal/internal olefins and carbonylation acitivity in aryl olefins. ....... 11
Supplementary Table 19 Comparison of product compositions of terminal/internal olefins carbonylation reactions. ....... 12
Supplementary Table 20 Substrate scope of alcohols for alkoxycarbonylation....... 12
Supplementary Table 21 Cycle performance. ....... 12
Supplementary Table 22 Effects of pyridine poison on styrene carbonylation....... 12
Supplementary Figure 1 Reaction kinetics of methoxycarbonylation. ....... 13
Supplementary Figure 2 The effects of the stirring speed on reaction rate of methoxycarbonylation. ....... 13
Supplementary Figure 3 The determination of apparent activation energy of methoxycarbonylation reactions....... 14
Supplementary Figure 4 Characterizations of $Ru_1/NbO_x$ and 2 wt.% $Ru/NbO_x$ catalyst. ....... 14
CO pulse chemisorption, Ru3p XPS data and CO-probe FTIR spectra of $Ru1/NbO_x$ and 2 wt.% $Ru/NbO_x$ catalyst....... 15
Supplementary Note 3 Characterizations of single-atom $Ru_1/NbO_x$ and nanoparticulate $Ru/NbO_x$....... 15
Supplementary Figure 5 HRTEM and lattice fringe of main exposed niobium pentoxide (001) facets in 2 wt.%$Ru/NbO_x$....... 15
Supplementary Figure 6 Physcial characterizations of Nb-based materials....... 15
Supplementary Figure 7 EXAFS fitting of $Ru/NbO_x$ catalyst. ....... 16
Supplementary Figure 8 In situ FTIR on $Nb_2O_5$ material at room temperature. ....... 16
Supplementary Figure 9 In situ FTIR on $NbO_x$ material at room temperature. ....... 17
Supplementary Note 4 In situ FTIR analysis of Supplementary Figure 9....... 17
Supplementary Figure 10 In situ FTIR surface reaction on 0.5 wt.%$Ru/Nb_2O_5$ through sequent adsorption. ....... 17
Supplementary Note 5 In situ FTIR analysis of Supplementary Figure 10....... 17
Supplementary Figure 11 In situ FTIR surface reaction on 0.5 wt.%$Ru/NbO_x$ through sequent adsorption....... 18
Supplementary Note 6 In situ FTIR analysis of Supplementary Figure 11....... 18
Supplementary Figure 12 Acidity quantification via pyridine-probe FTIR spectra....... 19
Supplementary Figure 13 Adsorption energy (eV) of styrene and CO on Ru surface....... 19

Supplementary Figure 14 Structures of the key intermediates in Figure 5. ........ 20

Supplementary Figure 15 The illustration scheme of in situ infrared spectroscopic system applied in this work. ........ 20

Supplementary Figure 16 The simulated infrared vibration frequency of *COOMe using Vienna Ab initio Simulation Package (VASP). 21

Supplementary Figure 17 Identification and test of gas in carbonylation reaction. ........ 21

Supplementary Figure 18 Regioselectivity regulation of methoxycarbonylation reactions on Ru/$NbO_x$. ........ 22

Supplementary Figure 19 Characterizations of Ru/$NbO_x$ catalysts before and after carbonylation reaction. ........ 22

Supplementary Figure 20 AC-HAADF STEM images and the corresponding Ru and Nb element map of the used 2 wt.% Ru/$NbO_x$........ 23

Supplementary Figure 21 GC spectra of MP production from ethylene methoxycarbonylation in a scale-up run........ 23

Supplementary Note 7 The structural data of $Nb_2O_5$, $NbO_x$ and Ru/$NbO_x$ support, the dissociative adsorption process of methanol, and the hydromethoxycarbonylation process of styrene were attached. The details include the optimized xyz coordinates, the corresponding total energy, thermodynamic zero point energy (*ZPE*) correction, and the entropy contribution ($T\Delta S$)........ 24

**Supplementary Table 1 Representative recent cases of alkoxycarbonylation of alkenes via heterogenous catalysis (Entry 1-7) and homogeneous catalysis (Entry 8-15).**

| Entry | Substrate | $n_{Sub}$ (mmol) | Catalyst | | | Nucleophile | Solvent | condition | | | Yield (%) | **TOF ($h^{-1}$)** | Reactor V (ml) | **CO utilization** (%) | **CO efficiency** ($mmol_{COO}\cdot mol_{CO}^{-1}\cdot h^{-1}$) | Reference |
|---|---|---|---|---|---|---|---|---|---|---|---|---|---|---|---|---|
| | | | **Metal** | **Ligand** | **Promoters** | | | CO (bar) | T/°C | t/h | | | | | | |
| 1 | styrene | 0.188 | **Ru/NbO$_x$** | | | MeOH | | 1.6 | 170 | 16 | 71 | **4.1** | 14.0 | 14.8 | **9.2** | **this work** |
| 2 | styrene | 1.88 | | | | MeOH | | 1.6 | 170 | 10 | 60 | **8.4** | 14 | 62.4 | **62.4** | |
| 3 | ethylene | 0.57 | | | | | | 1 | 170 | 1 | 33.6 | **33.8** | 14 | 33.6 | **336** | |
| 4 | ethylene | 1.13 | | | | MeOH | | 1 | 170 | 2 | 22.5 | **22.7** | 14 | 45.0 | **225** | |
| 5 | styrene | 0.5 | **Ru/CeO$_2$** | | | MeOH | | 2 | 165 | 14 | 80 | **1.4** | 46 | 10.8 | **7.7** | Chin. J. Catal. , 2020, 41, 963-969. |
| 6 | ethylene | 7.5 | **Ru/CeO$_2$** | | | MeOH | | 7 | 165 | 8 | 62 | **19.6** | 46 | 35.8 | **44.7** | J. Am. Chem. Soc. , 2018, 140, 4172-4181. |
| 7 | styrene | 5 | **Pd/LSI** | **TPPTS–OTPPTS** | | MeOH | toluene | 30 | 80 | 3.5 | 92.7 | **82.9** | 46 | 8.1 | **23** | Catal. Sci. Technol., 2014, 4, 1092-1103. |
| 8 | butadiene | 1 | **Pd(TFA)$_2$** | **HeMaRaphos** | **PTSA•H$_2$O** | nBuOH | toluene | 40 | 120 | 24 | 85 | **7.1** | 300.0 | 0.4 | **0.15** | Science, 2019, 366, 1514-1517. |
| 9 | butadiene | 1 | **Pd(TFA)$_2$** | **HeMaRaphos** | **PTSA•H$_2$O** | nBuOH | toluene | 40 | 120 | 120 | 64 | **533.3** | 300.0 | 0.4 | **0.07** | Science, 2019, 366, 1514-1517. |
| 10 | deca-1,3-diyn-1-ylbenzene | 0.25 | **Pd(TFA)$_3$** | **ferrocenediyl-pyridyl phosphine** | **PTSA•H$_2$O** | MeOH | toluene | 40 | 23 | 20 | 95 | **4.8** | 300.0 | 0.1 | **0.05** | Angew. Chem. Int. Ed. , 2019, 58, 10683-10687. |
| 11 | isoprene | 1.2 | **[Pd(cod)Cl$_2$]** | **Xantphos** | - | benzyl alcohol | toluene | 50 | 100 | 20 | 94 | **4.7** | 300.0 | 0.2 | **0.09** | Angew. Chem. Int. Ed. , 2014, 53, 9030-9034. |
| 12 | 1-phenylallene | 0.25 | **Pd(OAc)$_2$, Cu(OAc)$_2$** | **(S,S,S)-SKP** | **Et$_3$N, PhNH$_2$** | MeOH | MeOH | 1 | 25 | 24 | 89 | **0.4** | 2000.0 | 0.3 | **0.1** | J. Am. Chem. Soc. , 2015, 137, 15346-15349. |
| 13 | tetramethylethylene | 4 | **Pd(acac)$_2$** | **pytbpx** | **PTSA•H$_2$O** | MeOH | | 40 | 120 | 20 | 98 | **49.0** | 300.0 | 0.8 | **0.4** | Nat. Commun., 2017, 8, 14117. |
| 14 | styrene | 1 | **Pd(OAc)$_2$** | **PPh$_3$** | **FeCl$_3$** | $H_2O$ | 1,4-dioxane | 50 | 80 | 10 | 91 | **9.1** | 3.0 | 0.2 | **0.18** | Chem. Commun., 2018, 54, 3967--3970 |
| 15 | styrene | 0.5 | **PdCl$_2$** | **pyridylcarbazoylphosphines** | **MnBr(CO)$_5$** | MeOH | toluene | 60 | 120 | 20 | 85 | **4.3** | 4.0 | 0.1 | **0.04** | J. Catal. 2022, 409, 98-104. |

**Supplementary Note 1: CO utilization efficiency and TOF calculations in Supplementary Table 1.**

CO utilization (rate) is defined as the ratio of the amount of ester produced to the total amount of CO and calculated by the following equation. p, V, R and T represent the actual CO pressure (Pa), volume ($14\times10^{-6}$ $m^3$), molar gas constant (8.314 $J\cdot K^{-1}\cdot mol^{-1}$) and temperature (K). The p (Pa) equals the sum of gauge pressure ($p_g$, Pa) and atmospheric pressure ($p_0$, $10^5$ Pa).

$$CO\ \ utilization\ \ (\%)\ \ =\ \frac{n_{ester}}{n_{CO}}\times 100\% = \frac{n_{ester}}{pV/RT}\times 100\%$$

CO utilization efficiency ($h^{-1}$) is defined as the CO utilization rate per unit time (h) and calculated by CO utilization/t (h).

The TOF ($h^{-1}$) is defined as the molar amount of carbonylation produced per molar Ru metal per hour and calculated by equation.

$$TOF\ \ (h^{-1}) = \frac{n_{coo}}{m_{cat}\times wt.\%_{Ru}/M_{Ru}\times t}\ \times 100\%$$

The $n_{coo}$ is the molar amount of carbonylation (mol), which is equal to the molar amount of monocarbonylated esters or twice the molar amount of bicarbonylated esters. $M_{Ru}$ is the atomic weight of ruthenium (101.1 g/mol), $m_{cat}$ is the mass weight of the catalysts (g), wt.%$_{Ru}$ is the actual Ru weight percentage of the catalysts, and t is the reaction time (h).

In bicarbonylation reactions, the TOF ($h^{-1}$), CO utilization (%) and efficiency ($h^{-1}$), have been doubled according to the above equation, which have shown in Entry 8–10.

**Supplementary Table 2 Screening of effective heterogeneous catalysts for styrene methoxylcarbonylation.**

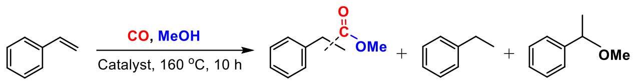


| Entry | Catalyst | Atmosphere (MPa) | Conversion (%) | Selectivity (%) | | | C. B. (%) |
|---|---|---|---|---|---|---|---|
| | | | | carbonylation | hydrogenation | methoxylation | |
| 1 | Pd/C | 1CO | 22 | 0 | 8 | 92 | 92±4 |
| 2 | Pt/C | 1CO | 18 | 0 | 9 | 91 | 98±2 |
| 3 | Ir/C | 1CO | 28 | 3 | 17 | 81 | 90±3 |
| 4 | Ru/C | 1CO | 100 | 6 | 74 | 5 | 101±2 |
| 5 | Ru/Hβ | 1CO | 59 | 1 | 3 | 93 | 83±5 |
| 6 | Ru/WZr$O_x$ | 1CO | 10 | 0 | 5 | 95 | 81±3 |
| 7 | Ru/$Al_2O_3$ | 1CO | 7 | 23 | 77 | 0 | 82±3 |
| 8 | Ru/$CeO_2$ | 1CO | 3 | 80 | 15 | 3 | 95±4 |
| 9 | Ru/$CeO_x$ | 1CO | 5 | 90 | 10 | 0 | 96±4 |
| 10 | Ru/$Nb_2O_5$ | 1CO | 7 | 87 | 6 | 7 | 98±2 |
| 11 | Ru/$NbO_x$ | 1CO | 13 | 90 | 8 | 2 | 91±4 |
| 12 | Ru/$NbO_x$ | 0.1CO | 55 | 90 | 10 | 0 | 101±2 |

Reaction conditions: 20 mg catalysts with 5 wt.% metal loading in Entry 1-4, 30 mg catalysts with 2 wt.% metal loading in Entry 5-12, styrene (0.2 mmol), MeOH (2 mL), CO, 160 °C, 10 h. The C.B. denotes carbon balance calculated based on benzene rings before and after reactions.

**Supplementary Table 3. The best-fitted EXAFS results of Ru/$NbO_x$[a].**

| Sample | Shell | CN | R (Å) | $\sigma^2$ ($10^{-2}$ Å$^2$ ) | $\Delta E_0$ (eV) | r-factor (%) |
|---|---|---|---|---|---|---|
| Ru foil | Ru-Ru | 12 | 2.67 | - | - | - |
| | Ru-O | 3.6±0.4 | 1.94±0.02 | 0.3±0.1 | -7.0±1.8 | |
| Ru/$NbO_x$ | Ru-Ru | 5.1±0.9 | 2.67±0.01 | 0.3±0.1 | -7.0±1.8 | 0.9 |
| | Ru-O-Nb/Ru | 1.3±0.4 | 3.08±0.05 | 0.3±0.1 | -7.0±1.8 | |

[a]CN is the coordination number for the absorber-backscattered pair, R is the average absorber-backscattered distance, $\sigma^2$ is the Debye-Waller factor, and $\Delta E_0$ is the inner potential correction. * $S_0^2$ was fixed to 0.72 as determined from Ru foil fitting. The data range used for data fitting in k-space (Δk) and R-space (ΔR) are 3.0-12.7 Å$^{-1}$ and 1.0-3.3 Å, respectively.

**Supplementary Table 4 Optimization of temperature for styrene carbonylation.**

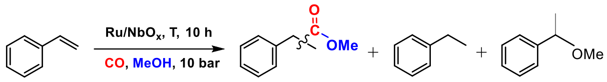


| Entry | Temperature(℃) | Conv. (%) | Sele.(%) | | | L/(L+B) (%) | C. B. (%) |
|---|---|---|---|---|---|---|---|
| | | | carbonylation | hydrogenation | methoxylation | | |
| 1 | 140 | 2 | 56 | 34 | 9 | 61 | 98 |
| 2 | 160 | 7 | 65 | 31 | 4 | 73 | 83 |
| 3 | 170 | 22 | 71 | 25 | 4 | 79 | 104 |
| 4 | 180 | 39 | 63 | 36 | 2 | 79 | 86 |
| 5 | 200 | 92 | 40 | 56 | 1 | 79 | 107 |

Reaction conditions: 2 wt.% Ru/NbO$_x$ (30 mg, 5.6 umol Ru), styrene (0.2 mmol), $H_3BO_3$ (4.0 mmol, 5 mol%), MeOH (2.0 mL), CO (10 bar), 10 h. The L/(L+B) denotes the ratio of linear esters to branched esters.

**Supplementary Table 5 Pressure-dependent evolutions of styrene carbonylation.**

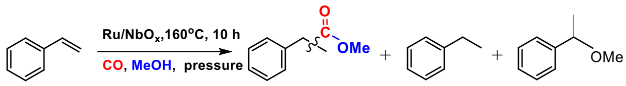


| Entry | pressure (bar) | Conv. (%) | Sele.(%) | | | L/(L+B) (%) | C. B. (%) |
|---|---|---|---|---|---|---|---|
| | | | carbonylation | hydrogenation | methoxylation | | |
| 1 | 0.6 | 24 | 84 | 16 | 0 | 74 | 92 |
| 2 | 1.0 | 15 | 88 | 12 | 0 | 73 | 102 |
| 3 | 3.5 | 5 | 80 | 20 | 0 | 71 | 86 |
| 4 | 5.0 | 4 | 77 | 23 | 0 | 66 | 83 |

Reaction conditions: 1 wt.% Ru/$NbO_x$ (30 mg, 5.6 umol Ru), styrene (0.2 mmol), MeOH (2.0 mL), CO, 160 ºC, 10 h.

**Supplementary Table 6 Effects of catalyst weight loadings on styrene carbonylation.**

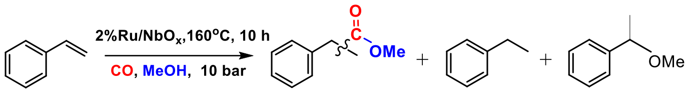


| Entry | catalyst mass (mg) | Conv. (%) | Sele.(%) | | |
|---|---|---|---|---|---|
| | | | carbonylation | hydrogenation | methoxylation |
| 1 | 20 | 4 | 87 | 7 | 6 |
| 2 | 30 | 5 | 87 | 9 | 4 |
| 3 | 100 | 19 | 83 | 11 | 1 |

Reaction conditions: 2 wt.% Ru/$NbO_x$, styrene (0.2 mmol), MeOH (2.0 mL), CO (10 bar), 160 ºC, 10 h.

**Supplementary Table 7 Effects of styrene concentration on styrene carbonylation.**

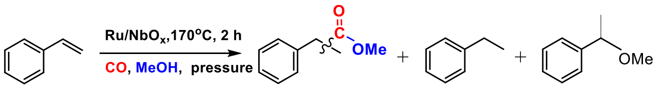


| Entry | styrene | Conv. (%) | Yield (%) | | | L/(L+B) (%) |
|---|---|---|---|---|---|---|
| | | | carbonylation | hydrogenation | methoxylation | |
| 1 | 1 eq. | 26 | 22 | 4 | 0 | 75 |
| 2 | 3.5 eq. | 24 | 21 | 2 | 0 | 75 |
| 3 | 10 eq. | 21 | 19 | 2 | 0 | 75 |
| 4* | 10 eq. | 24 | 20 | 3 | 0 | 76 |

Reaction conditions: 2 wt.% Ru/$NbO_x$ (30 mg), styrene (1 eq, 0.188 mmol), MeOH (2.0 mL), CO (1 bar), 170 ºC, 2 h. *(0.6 bar).

**Supplementary Table 8 Effects of solvent for styrene carbonylation.**

| Entry | Solvent | Conv. (%) | Yield (%) | | | L/(L+B) (%) |
|---|---|---|---|---|---|---|
| | | | carbonylation | hydrogenation | methoxylation | |
| 1 | 2-MTHF | 16 | 1 | 15 | 0 | 68 |
| 2 | cyclohexane | 15 | 4 | 9 | 1 | 83 |
| 3 | toluene | 22 | 7 | 13 | 1 | 85 |
| 4 | methanol | 95 | 71 | 24 | 0 | 75 |

Reaction conditions: 2 wt.% Ru/$NbO_x$, styrene (0.2 mmol), solvent (2.0 mL), CO (1 bar), 170 ºC, 16 h.

**Supplementary Table 9 Effects of water for styrene carbonylation.**

| Entry | amount of $H_2O$ | Conv. (%) | Yield (%) | | | L/(L+B)(%) | C. B. (%) |
|---|---|---|---|---|---|---|---|
| | | | carbonylation | hydrogenation | methoxylation | | |
| 1 | 0 | 68 | 56 | 12 | 0 | 75 | 94 |
| 2 | 3 eq. | 71 | 48 | 22 | 0 | 75 | 89 |
| 3 | 7 eq. | 75 | 56 | 19 | 0 | 76 | 93 |
| 4 | 21 eq. | 3 | 0 | 3 | 0 | - | 91 |

Reaction conditions: 2 wt.% $Ru/NbO_x$ (30 mg), styrene (0.2 mmol, 1 eq.), MeOH (2.0 mL), CO (1 bar), 170 ºC, 10 h.

**Supplementary Table 10 Effects of reaction atmosphere for styrene carbonylation.**

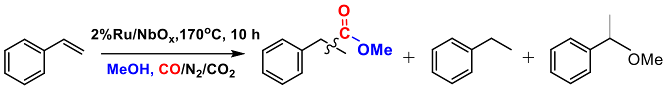


| Entry | Atmosphere (bar) | Conv. (%) | Yield (%) | | | L/(L+B) (%) | C. B. (%) |
|---|---|---|---|---|---|---|---|
| | | | carbonylation | hydrogenation | methoxylation | | |
| 1 | 1CO | 68 | 56 | 12 | 0 | 75 | 94 |
| 2 | $1CO/10N_2$ | 14 | 9 | 5 | 0 | 73 | 90 |
| 3 | $1CO/2CO_2$ | 37 | 28 | 9 | 0 | 75 | 93 |
| 4 | $1CO/2CO_2/H_2O$ | 9 | 5 | 4 | 0 | 74 | 85 |

Reaction conditions: 2 wt.% $Ru/NbO_x$ (30 mg), styrene (0.2 mmol), MeOH (2.0 mL), $CO/N_2/CO_2$/0.4 mmol $H_2O$, 170 ºC, 10 h.

**Supplementary Table 11 Ru dispersion, CO pulse chemisorption data and ICP-OES data of $Ru_1/NbO_x$ and 2 wt.% $Ru/NbO_x$ catalysts.**

| Sample | Ru wt.% from ICP-OES[a] | CO accumulative sorption quantity (mmol/g)[b] | Ru dispersion[b] | Ru active diameter (nm)[b] |
|---|---|---|---|---|
| $Ru_1/NbO_x$ | 0.098% | 0.00969 | 99.8% | 1.1 |
| 2 wt.% $Ru/NbO_x$ | 1.891% | 0.08172 | 44.4% | 2.2 |
| 4 wt.% $Ru/NbO_x$ | 3.331% | 0.14432 | 43.8% | 3.1 |

[a]The ruthenium loading was determined by ICP-OES.

[b]CO pulse chemisorption.

**Supplementary Table 12 Effects of fully dispersed Ru and size-dependent Ru clusters on styrene carbonylation.**

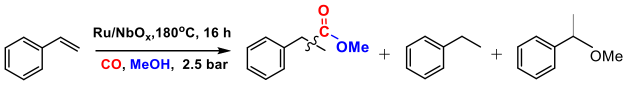


| Entry | catalysts | Conv. (%) | Yield (%) | | | L/(L+B) (%) | C. B. (%) |
|---|---|---|---|---|---|---|---|
| | | | carbonylation | hydrogenation | methoxylation | | |
| 1 | $Ru_1/NbO_x$ | 0.8 | 0 | 0.8 | 0 | - | 90 |
| 2 | $Ru/NbO_x$-2.2 nm | 80 | 62 | 17 | 0 | 75 | 97 |
| 3 | $Ru/NbO_x$-3.1 nm | 42 | 14 | 27 | 0 | 74 | 95 |

Reaction conditions: catalyst (30 mg), styrene (0.2 mmol), MeOH (2.0 mL), CO (2.5 bar), 180 ºC, 16 h.

**Supplementary Table 13 The deconvolution of CO-adsorption FTIR spectra on 2 wt.% Ru/$NbO_x$ catalysts (Figure 2h).**

| No. | Peak($cm^{-1}$) | Species | Factor of $n_{Ru}/n_{CO}$ | Ratio of peak area(%) |
|---|---|---|---|---|
| 1 | 2140 | $Ru(CO)_3$ | 1/3 | 3.4 |
| 2 | 2081 | $Ru(CO)_3$ | 1/3 | 13.8 |
| 3 | 2128 | $Ru(CO)_2$ | 1/2 | 1.1 |
| 4 | 2063 | $Ru(CO)_2$ | 1/2 | 11.6 |
| 5 | 2015 | Ru(CO) | 1 | 52.1 |
| 6 | 1969 | Ru(CO) | 1 | 18.0 |

**Supplementary Note 2 Calculation of the average factor of $n_{Ru}/n_{CO}$.**

$$AF_{Ru/CO} = \frac{\sum_1^3 A_{Ru(CO)_n} \times \varepsilon \times 1/n}{\sum_1^3 A_{Ru(CO)_n}} = \ 1/n\sum_1^3 R_{Ru(CO)_n} = 1/3R_{Ru(CO)_3} + 1/2R_{Ru(CO)_2} + R_{Ru-CO}$$

The $A_{Ru(CO)n}$ and $\varepsilon$ represent peak areas and extinct coefficients ($\varepsilon_{linear} \approx 0.1\varepsilon_{bridge}$), and $R_{Ru(CO)n}$ are the proportions of the peak area of the $Ru(CO)_n$ ($n$ = 1, 2, 3) species, and evaluated from the peak deconvolution of the CO-adsorption FTIR spectra (Supplementary Table 13). No bridge-bonded CO peak (<1900 $cm^{-1}$) was observed on Ru/$NbO_x$. Based on the results of Supplementary Table 13, the $AF_{Ru/CO}$ is 0.822 and the calibrated Ru dispersion of 2 wt.%Ru/$NbO_x$ catalysts is 36.4%.

**Supplementary Table 14 The reported band assignment of various Ru carbonyl species absorbed on oxide in CO-probed infrared spectra.**

| Adsorbed species | Ru adsorption sites | Frequency ($cm^{-1}$) | Samples | Ref. |
|---|---|---|---|---|
| tricarbonyl species $Ru^{n+}(CO)_3$ | $Ru^{n+}$ (n=1-3) | 2142-2028, 2082-2067 | Ru/$Nb_2O_5$, Ru/$TiO_2$, Ru/$SiO_2$, Ru/$Al_2O_3$ | 1-5 |
| | $(SiO)_xRu^{n+}$ (n=1-3)<br>$(RuO)_xRu^{n+}$ (n=1-3) | 2140, 2080<br>2130, 2070 | Ru/$SiO_2$ | 2-3 |
| dicarbonyl species $Ru(CO)_2$ | $Ru^{2+}$ | 2132, 2070 | Ru/$SiO_2$ | 2 |
| | $Ru^0$ | 2080, 2015 | Ru/$SiO_2$ | 2 |
| linear Ru-CO | $Ru^{n+}$ (n=1-3) | 2110-2080 | Ru/$SiO_2$ | 2, 3 |
| | $Ru^0$ | 2050-2010 | Ru/$SiO_2$, Ru/$Nb_2O_5$, Ru/$TiO_2$ | 1-5 |
| | $Ru^{\delta+}$ | 1995 | Ru/$TiO_2$ | 2,4 |
| | interfacial ($TiO_2$)Ru | 1975 | Ru/$TiO_2$ | 4 |
| | | 1950 | Ru/$TiO_2$ | 5 |
| bridged $Ru_2$-CO | $Ru^0$ | 1990-1750 | Ru/$SiO_2$ | 3 |
| | $Ru^0$ | 1810 | Ru/$TiO_2$ | 4 |


[1] Komanoya, T.; Kinemura, T.; Kita, Y.; Kamata, K.; Hara, M. ***J. Am. Chem. Soc.*** 139 (2017) 11493-11499.
[2] S.Y. Chin, C.T. Williams, M.D. Amiridis, ***J. Phys. Chem. B*** 110 (2006) 871-882.
[3] G.H. Yokomizo, C. Louis, T. Bell, ***J. Catal.*** 120 (1989) 1-14.
[4] Panagiotopoulou, P.; Kondarides, D. I.; Verykios, X. E. ***J. Phys. Chem. C*** 115 (2011) 1220-1230.
[5] Zhou, J.; Gao, Z.; Xiang, G.; Zhai, T.; Liu, Z.; Zhao, W.; Liang, X.; Wang, L ***Nat. Commun.*** 13 (2022) 327.


**Supplementary Table 15 Effects of active Ru components on styrene carbonylation.**

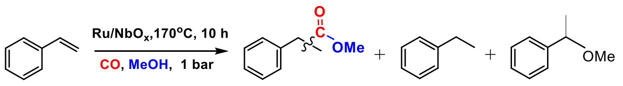


| Catalyst | Mass (g) | Time (h) | Conv. (%) | Yield (%) | | | L/(L+B) (%) | C. B. (%) |
|---|---|---|---|---|---|---|---|---|
| | | | | carbonylation | hydrogenation | methoxylation | | |
| Ru/$NbO_x$ | 20 | 10 | 54 | 38 | 15 | 0 | 75 | 90 |
| $Ru^0$/$NbO_x$ | 20 | 10 | 28 | 18 | 10 | 0 | 67 | 84 |
| $Ru^{3+}$/$NbO_x$ | 20 | 10 | 78 | 47 | 30 | 1 | 78 | 95 |

Reaction conditions: styrene (0.2 mmol), MeOH (2.0 mL), CO (1 bar), 170 ºC. The Ru loading of each catalysts is 2 wt.%.

In contrast, $RuCl_3/NbO_x$ catalysts showed a similar carbonylation yield in comparison to 2 wt.% $Ru/NbO_x$, due to in-situ reduction by active H or $H_2$ produced during the reaction (Supplementary Fig. 17).

**Supplementary Table 16 Substrate scopes of substituted vinyl arenes.**

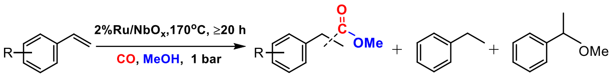


| substrate | time/h | Conv.(%) | Yield (%) | | | L/(L+B) (%) |
|---|---|---|---|---|---|---|
| | | | carbonylation | hydrogenation | methoxylation | |
| 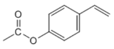 | 20 | 100 | 40 | 45 | 15 | 72 |
| 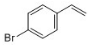 | 20 | 100 | 52 | 48 | 0 | 74 |
| 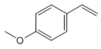 | 20 | 96 | 46 | 30 | 20 | 72 |
| 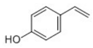 | 23 | 95 | 34 | 47 | 14 | 70 |
| 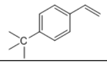 | 20 | 72 | 50 | 20 | 2 | 74 |

Reaction conditions: 2 wt.% $Ru/NbO_x$ (30 mg), substrate (0.2 mmol), MeOH (2.0 mL), CO (1 bar), 170 ºC.

**Supplementary Table 17 Dependence of side chains of aryl olefins on their carbonylation performance.**

| substrate | p/MPa | T/℃ | t/h | Conv.(%) | Yield (%) | | | | L/(L+B) (%) |
|---|---|---|---|---|---|---|---|---|---|
| | | | | | carbonylation | isomerisation | hydrogenation | methoxylation | |
| 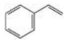 | 0.1 | 170 | 16 | 96 | 71 | 0 | 24 | 0 | 75 |
| 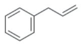 | 0.1 | 170 | 23 | 100 | 21 | 69 | 9 | 0 | 53 |
| 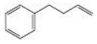 | 0.1 | 170 | 21 | 99 | 25 | 68 | 8 | 0 | 48 |

Reaction conditions: 2 wt.% $Ru/NbO_x$ (30 mg), substrate (0.2 mmol), MeOH (2.0 mL).

**Supplementary Table 18 Dependence between terminal/internal olefins and carbonylation acitivity in aryl olefins.**

| substrate | p/MPa | T/℃ | t/h | Conv.(%) | Yield (%) | | | | Linear ratio (%) |
|---|---|---|---|---|---|---|---|---|---|
| | | | | | carbonylation | isomerisation | hydrogenation | methoxylation | |
| 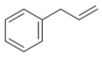 | 0.1 | 170 | 23 | 100 | 21 | 69 | 9 | 0 | 53 |
| 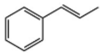 | 0.1 | 170 | 23 | 37 | 20 | 6 | 9 | 0 | 57 |
| 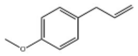 | 0.1 | 170 | 20 | 100 | 17 | 77 | 6 | 0 | 35 |
| 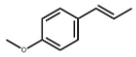 | 0.1 | 170 | 20 | 42 | 20 | 5 | 17 | 0 | 39 |

Reaction conditions: 2 wt.% Ru/$NbO_x$ (30 mg), substrate (0.2 mmol), MeOH (2.0 mL).

**Supplementary Table 19 Comparison of product compositions of terminal/internal olefins carbonylation reactions.**

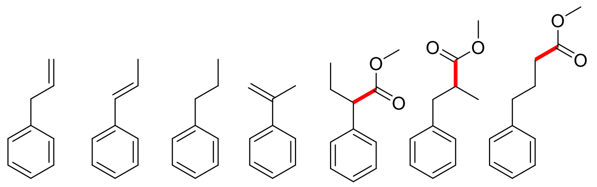

2a 2b 2c 2d 2e 2f 2g

2a 100% —Ru/NbOx, 1 bar CO, MeOH, 170°C, 20 h→ 2b + 2c + 2d + 2e + 2f + 2g
63% 9% 6% 2% 8% 11%

2b 37% —Ru/NbOx, 1 bar CO, MeOH, 170°C, 20 h→ 2b + 2c + 2d + 2e + 2f + 2g
63% 9% 6% 0% 9% 12%

Conversion Composition

**Supplementary Table 20 Substrate scope of alcohols for alkoxycarbonylation.**

| substrate | p/MPa | T/℃ | t/h | Conv.(%) | Selectivity (%) | | | L/(L+B) (%) |
|---|---|---|---|---|---|---|---|---|
| | | | | | carbonylation | hydrogenation | methoxylation | |
| MeOH | 0.1 | 170 | 18 | 98 | 81 | 19 | 0 | 75 |
| EtOH | 0.1 | 170 | 16 | 86 | 52 | 48 | 0 | 73 |
| PrOH | 0.1 | 170 | 18 | 79 | 32 | 32 | 36 | 74 |
| i-PrOH | 0.1 | 170 | 16 | 78 | 21 | 73 | 6 | 73 |

Reaction conditions: 2 wt.%Ru/$NbO_x$ (30 mg), substrate (0.2 mmol), solvent (2.0 mL).

**Supplementary Table 21 Cycle performance.**

| Entry | Substrate | Cylce | Conversion (%) | Yield of carbonylation (%) |
|---|---|---|---|---|
| 1 | ethylene | 1 | 32 | 29 |
| 2 | | 2 | 3 | 3 |
| 3 | | 3 | 4 | 4 |
| 4 | styrene | 1 | 70 | 52 |
| 5 | | 2 | 7 | 2 |

Reaction conditions: [a]2 wt.%Ru/$NbO_x$ (30 mg), ethylene (0.57 mmol), MeOH (2.0 mL), CO (1 bar), 170 °C, 2 h. [b]2 wt.%Ru/$NbO_x$ (30 mg), styrene (0.2 mmol), MeOH (2.0 mL), CO (1 bar), 170 °C, 10 h.

**Supplementary Table 22 Effects of pyridine poison on styrene carbonylation.**

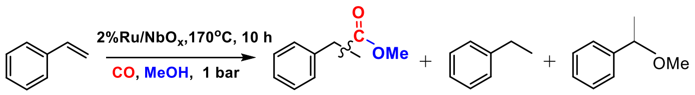


| Entry | Poison | Conv. (%) | Yield.(%) | | |
|---|---|---|---|---|---|
| | | | carbonylation | hydrogenation | methoxylation |
| 1 | pyridine | 74 | 49 | 16 | 0 |
| 2 | no | 73 | 50 | 14 | 0 |

Reaction conditions: 2 wt.%Ru/$NbO_x$ (30 mg), styrene (0.2 mmol), pyridine (6.3 μmol), MeOH (2.0 mL), CO (1 bar), 170 °C,10 h.

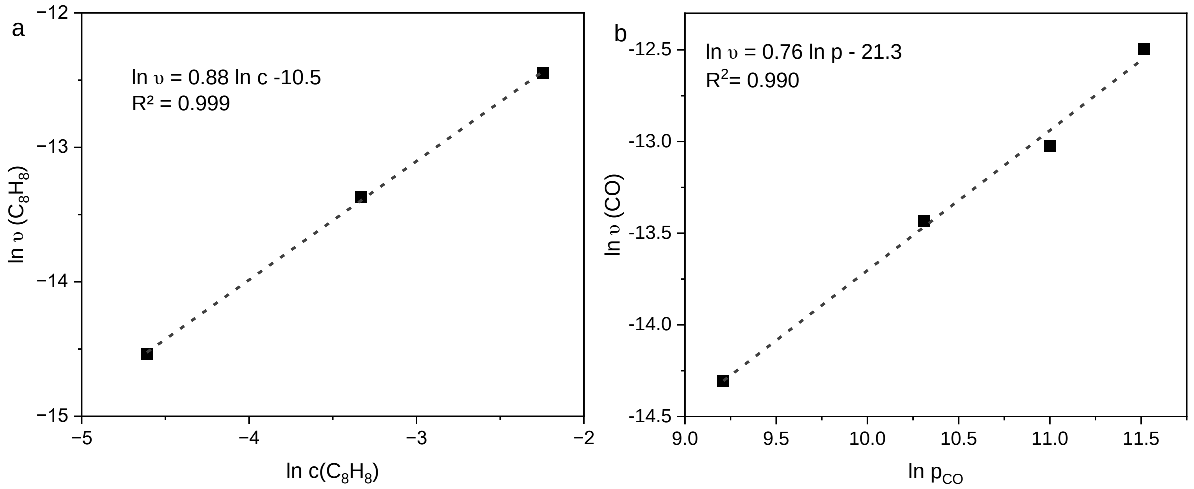


**Supplementary Figure 1 Reaction kinetics of methoxycarbonylation.**

(a) Reaction orders for styrene (a) and CO (b). Reaction conditions: (a) 20 mg Ru/$NbO_x$, styrene (0.01~0.1 mol/L), 2 mL MeOH, CO 0.1 MPa, 600 rpm, 170 °C for 2 h. (b) 20 mg Ru/$NbO_x$, styrene (0.1 mol/L), 2 mL MeOH, CO ($10^4$ Pa ~$10^5$ Pa), 600 rpm, 170 °C for 2 h.

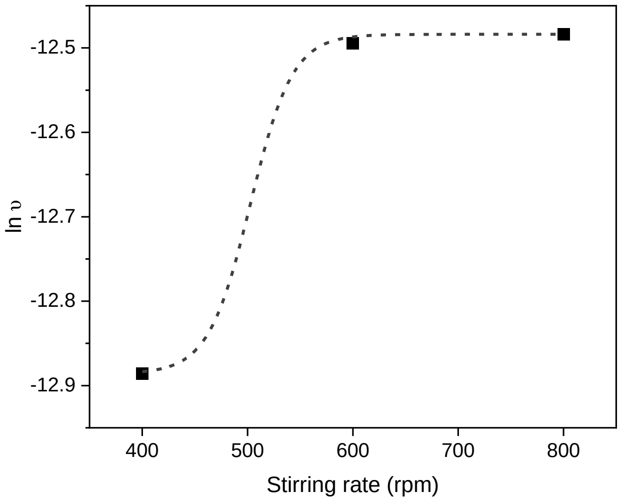


**Supplementary Figure 2 The effects of the stirring speed on reaction rate of methoxycarbonylation.**

Reaction conditions: 20 mg Ru/$NbO_x$, 0.2 mmol styrene, 2 mL MeOH, CO 0.1 MPa, 170°C for 2 h, stirring rate (400, 600, 800 rpm).

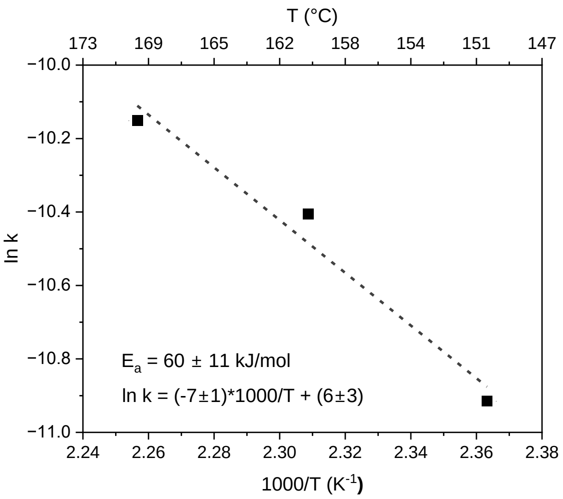


**Supplementary Figure 3 The determination of apparent activation energy of methoxycarbonylation reactions.**

Reaction conditions: 20 mg cat, 0.2 mmol styrene, 2 mL MeOH, CO 0.1 MPa, 600 rpm, 150~180 °C for 2 h.

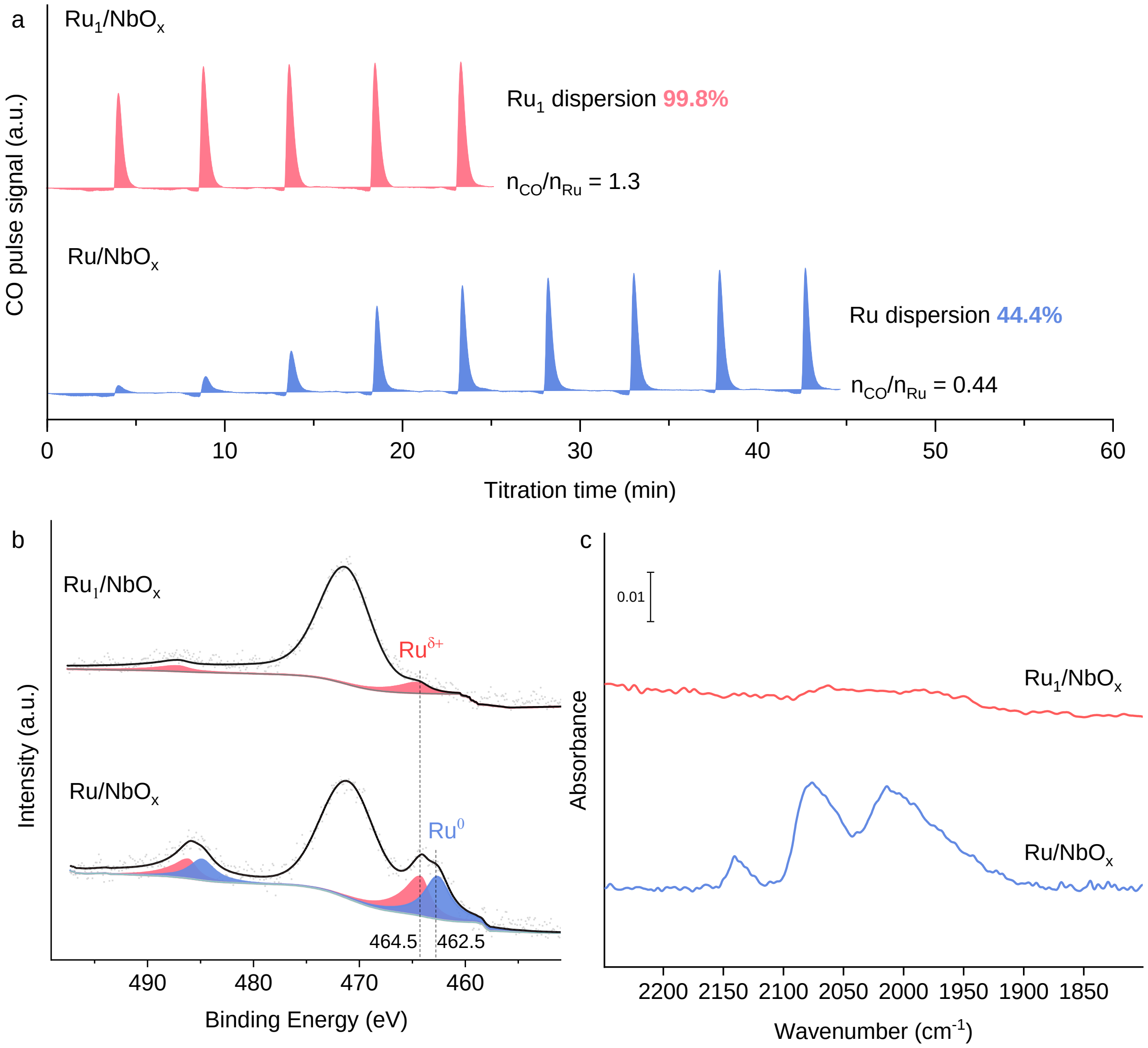


**Supplementary Figure 4 Characterizations of $Ru_1/NbO_x$ and 2 wt.% $Ru/NbO_x$ catalyst.**

CO pulse chemisorption, Ru3p XPS data and CO-probe FTIR spectra of Ru1/$NbO_x$ and 2 wt.% Ru/$NbO_x$ catalyst.

**Supplementary Note 3 Characterizations of single-atom $Ru_1/NbO_x$ and nanoparticulate $Ru/NbO_x$.**

The CO adsorption volume on the catalysts was meticulously measured via CO pulse chemisorption (Supplementary Figure 4), while the average factor of $n_{Ru}/n_{CO}$ was derived from CO-probe FTIR spectra (Supplementary Table 13). These measurements were utilized to calculate Ru dispersion, as detailed in the Materials and Methods section of the main text.

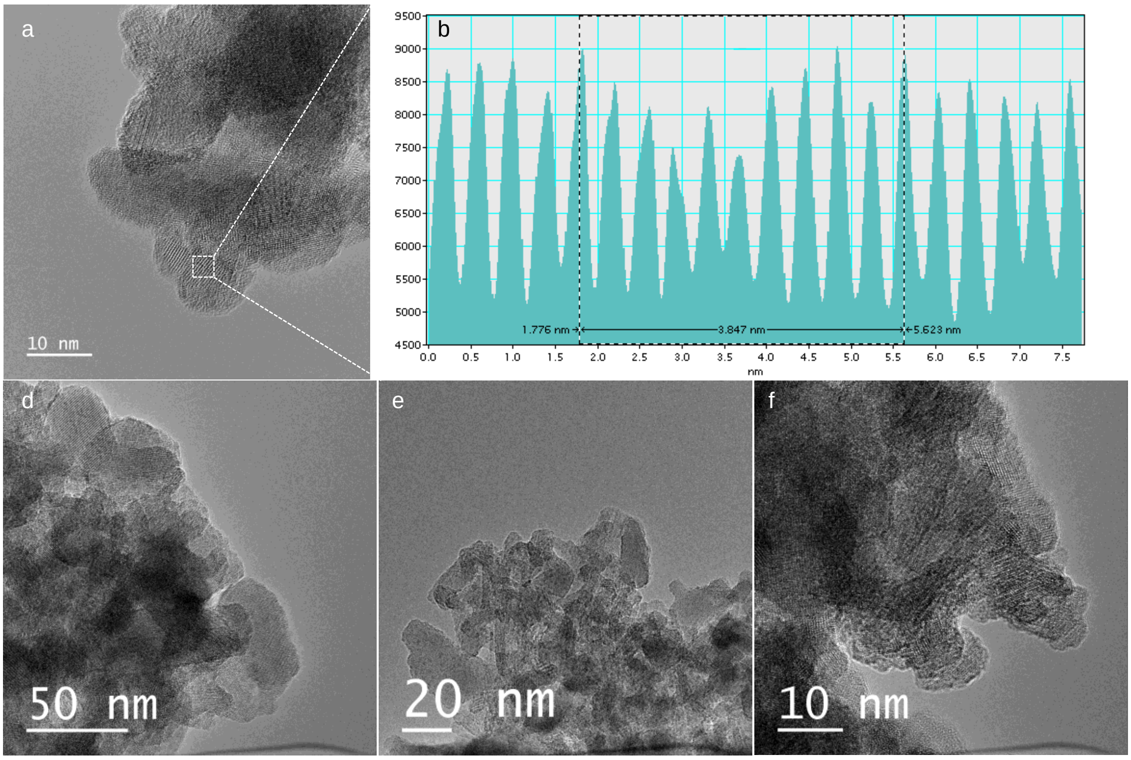


**Supplementary Figure 5 HRTEM and lattice fringe of main exposed niobium pentoxide (001) facets in 2 wt.%Ru/$NbO_x$.**

The units for the values in Figure (b) is arbitrary unit.

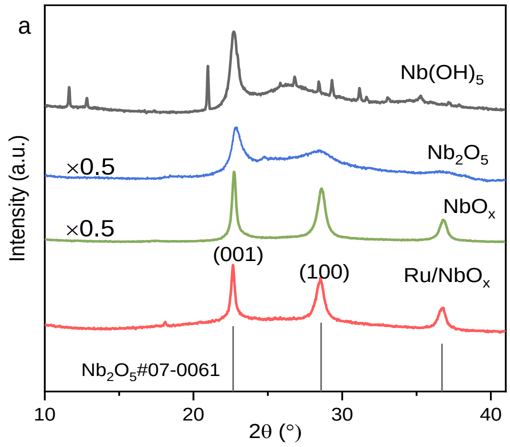


b

| samples | $O_V$ site concentration (spins/g) |
|---|---|
| $Nb(OH)_5$ | $5.679*10^{14}$ |
| $Nb_2O_5$ | $6.420*10^{14}$ |
| $Ru/Nb_2O_5$ | $8.090*10^{14}$ |
| $NbO_x$ | $1.208*10^{15}$ |
| $Ru/NbO_x$ | $1.739*10^{15}$ |

**Supplementary Figure 6 Physcial characterizations of Nb-based materials.**

(a) XRD patterns and (b) quantitative EPR data on oxygen vacancy concentrations.

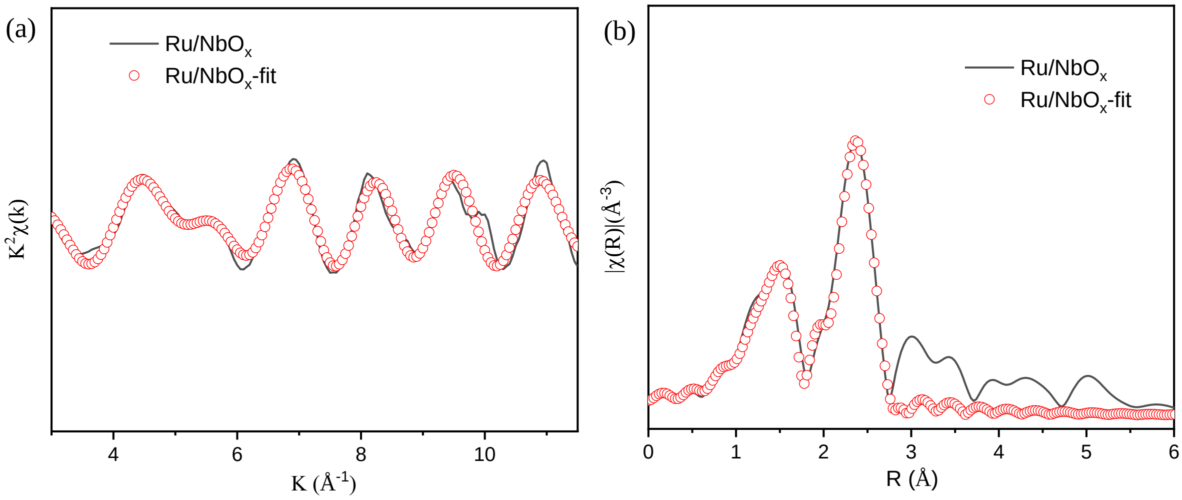


**Supplementary Figure 7 EXAFS fitting of Ru/NbO$_x$ catalyst.**

(a) Ru K-edge EXAFS fitting curve, shown in k$^2$ weighted *k*-space. (b) EXAFS fitting curve in the region of 1.0-3.3 Å, shown in k$^2$ weighted *R*-space.

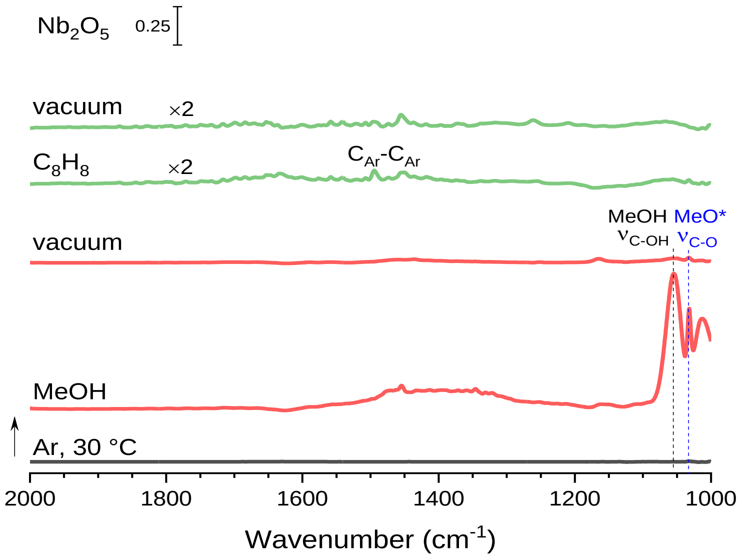


**Supplementary Figure 8 In situ FTIR on Nb$_2$O$_5$ material at room temperature.**

The preparation method of Nb$_2$O$_5$ with fewer oxygen defects was detailed in Methods and infrared experiments are given in Methods as well.

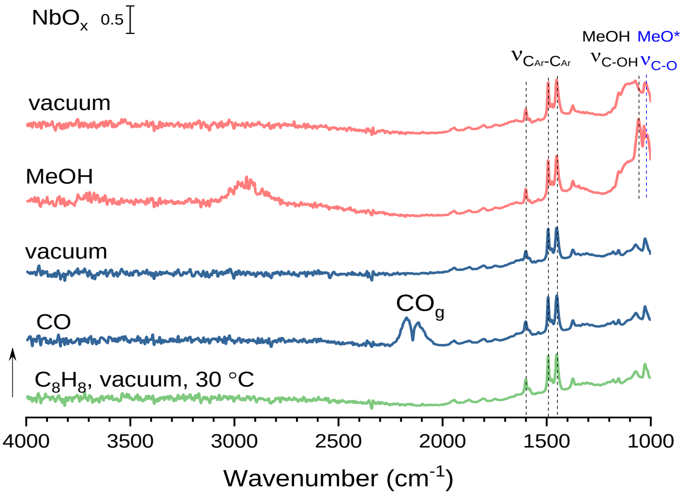


**Supplementary Figure 9 In situ FTIR on $NbO_x$ material at room temperature.**

The preparation method of $NbO_x$ with abundant oxygen defects was detailed in Methods and infrared experiments are given in Methods as well.

**Supplementary Note 4 In situ FTIR analysis of Supplementary Figure 9.**

As shown in Supplementary Figure 9, styrene can be adsorbed on $NbO_x$ support, because the characteristic vibrations of aromatic ring were presented in 1600, 1490 and 1450 $cm^{-1}$ after the adsorption of styrene in an infrared cell. Then pure CO gas (40 mL/min) passed through the cell for a while until the signal kept unchanged, and vacuumed until the gaseous CO peak was removed thoroughly. Following that, methanol was bubbled by Ar (40 mL/min) into the cell. Then MeOH adsorbed and $CH_3O^*$ generated from methanol dissociation in support can be observed, as suggested from the stretching vibrations of C-OH and C-O bonds. Through vacuum, the adsorbed methanol molecules were taken away and leaving $CH_3O^*$ species on the surface of catalysts.

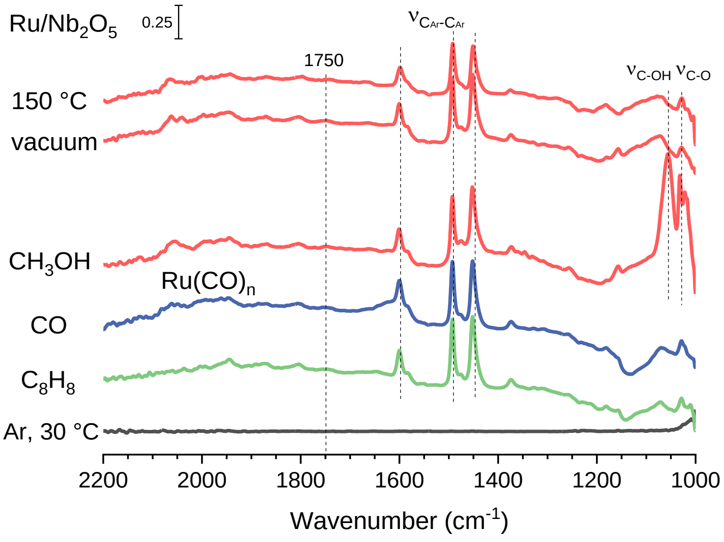


**Supplementary Figure 10 In situ FTIR surface reaction on 0.5 wt.%Ru/$Nb_2O_5$ through sequent adsorption.**

**Supplementary Note 5 In situ FTIR analysis of Supplementary Figure 10.**

In a typical FTIR, a self-supported catalyst wafer with 13 mm diameter was treated in 10% $H_2$/Ar at 350 °C for 30 min with a heating rate of 10 °C/min. Once cooling, the atmosphere was switched to Ar (40 mL/min). The background spectrum was recorded at 30 °C when the signal kept unchanged. The spectrum was shown here (the bottom).Subsequently, styrene was bubbled by Ar (40 mL/min) until unchanged. Pure Ar (40 mL/min) was introduced untill unchanged. The steady state spectra was shown here (green spectrum).Then the pure CO gas was introduced into the reaction cell until the spectra were unchanged and then pure Ar (40

mL/min) was introduced untill unchanged. The steady state spectra were shown here.
The cell was vacuumized and methanol vapour was introduced by saturation adsorption tube until unchanged. The steady state spectra were shown here. Then it was vacuumized for 5 minutes and record the spectra. Finally, the temperature was programmed to the desired temperature and collected the spectra.

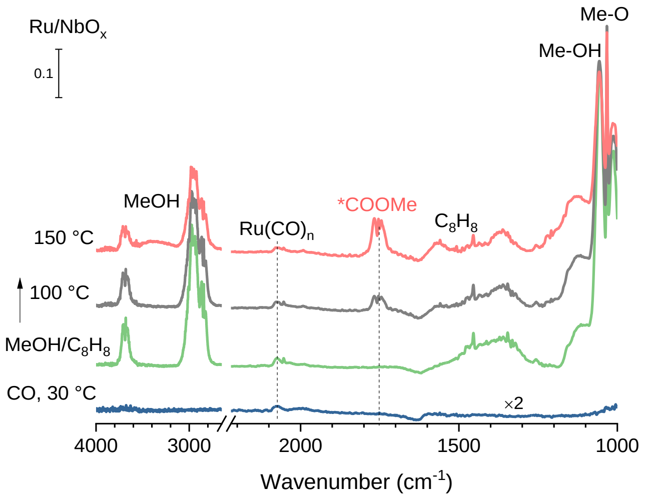


**Supplementary Figure 11 In situ FTIR surface reaction on 0.5 wt.%Ru/NbO$_x$ through sequent adsorption.**

**Supplementary Note 6 In situ FTIR analysis of Supplementary Figure 11.**

In a typical FTIR, a self-supported catalyst wafer with 13 mm diameter was treated in 10% $H_2$/Ar at 350 °C for 30 min with a heating rate of 10 °C/min. Once cooling, the atmosphere was switched to Ar (40 mL/min). The background spectrum was recorded at 30 °C when the signal kept unchanged. The pure CO gas (40 mL/min) was introduced into the reaction cell until the spectrum were unchanged. Pure Ar (40 mL/min) was introduced untill unchanged. The steady state spectrum was shown here (the bottom spectra). The methanol/styrene (1/10 V/V) mixture was bubbled by Ar (40 mL/min) until unchanged. Pure Ar (40 mL/min) was introduced the spectrum untill unchanged. The steady state spectrum were shown here. The temperature was programmed to the desired temperature and collected the spectra.

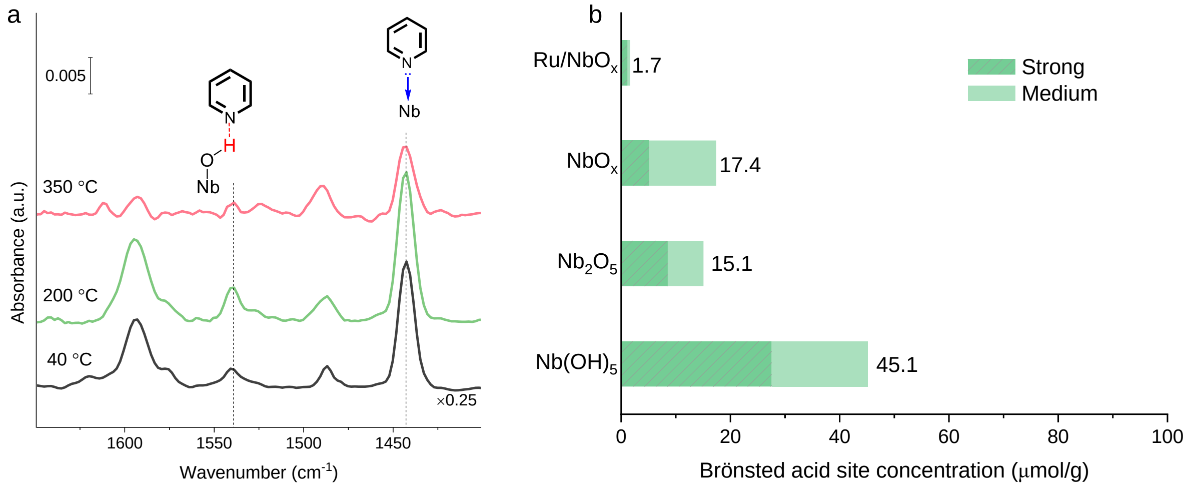


**Supplementary Figure 12 Acidity quantification via pyridine-probe FTIR spectra.**

(a) Pyridine-probe FTIR spectra of Ru/$NbO_x$ catalyst at 40 °C, 200 °C and 350 °C, respectively. (b) The concentrations of Brönsted acid sites of Ru/$NbO_x$ equivalents at 200 °C.

| Adsorbate \ Substrate | $NbO_x$ | Ru |
|---|---|---|
| methanol | -0.96 | -0.61 |
| styrene | -0.98 | -3.46 |
| CO* | -0.23 | -1.65 |

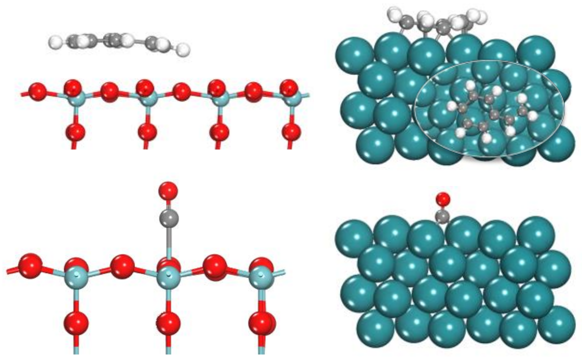

**Supplementary Figure 13 Adsorption energy (eV) of styrene and CO on Ru surface**.

The corresponding adsorption models are also attached.

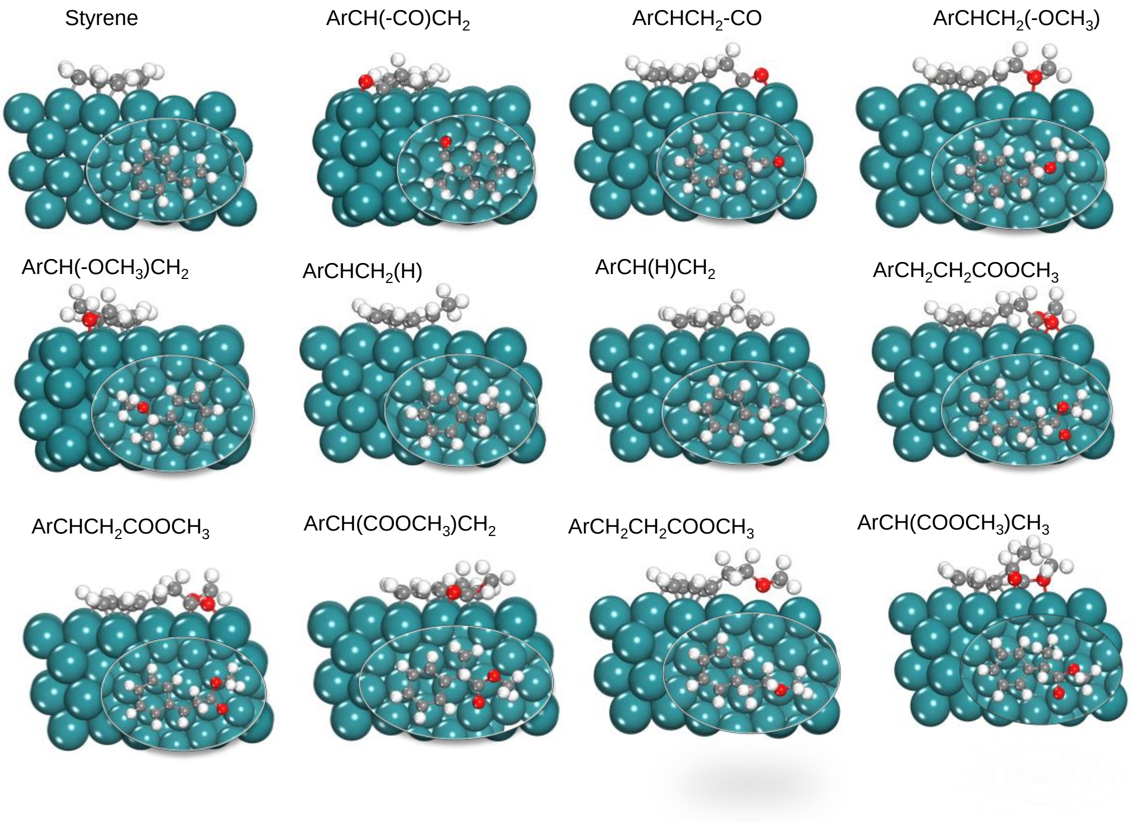


**Supplementary Figure 14 Structures of the key intermediates in Figure 5.**

Nb, O, C and H atoms are shown in green, red, grey and white, respectively. More details see Methods and Supplementary note 7.

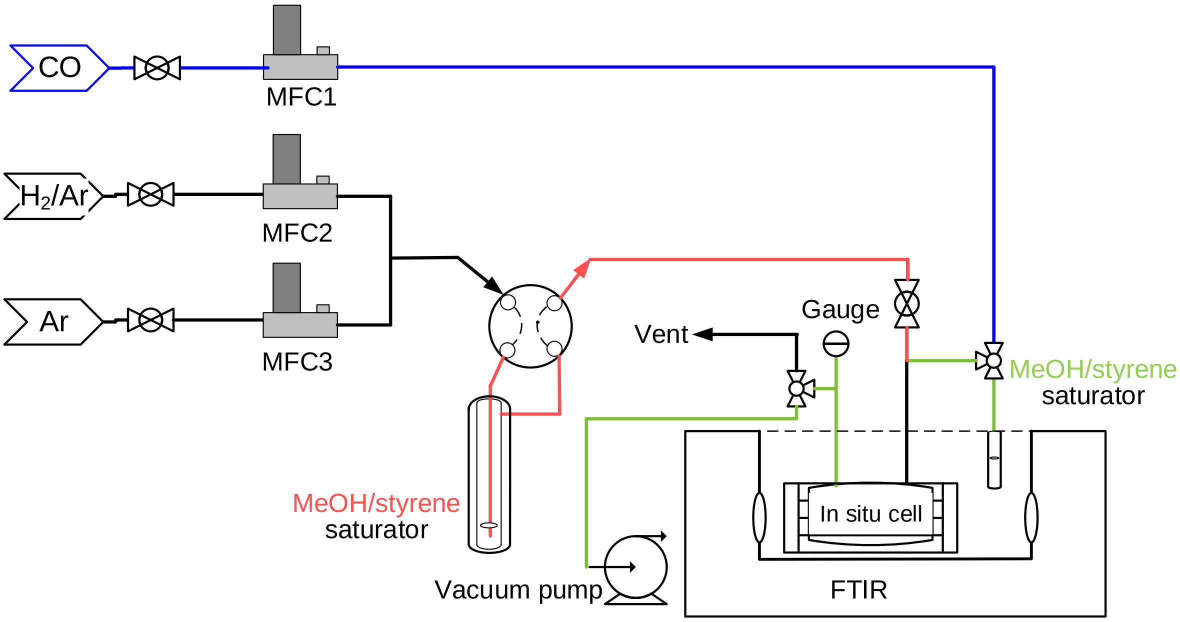


**Supplementary Figure 15 The illustration scheme of in situ infrared spectroscopic system applied in this work.**

The in situ infrared experiments are detailed in Methods.

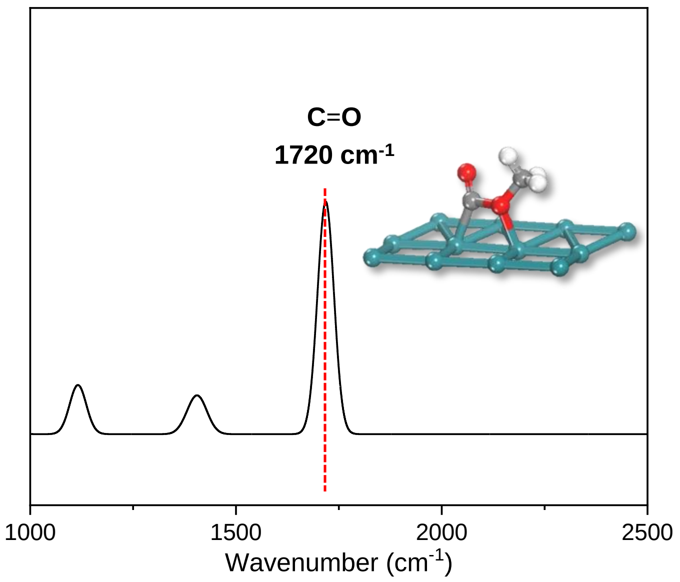


**Supplementary Figure 16 The simulated infrared vibration frequency of *COOMe using Vienna Ab initio Simulation Package (VASP).**

The stretching vibration frequency of C=O of *COOMe with strong oscillator strength is located at 1720 $cm^{-1}$.

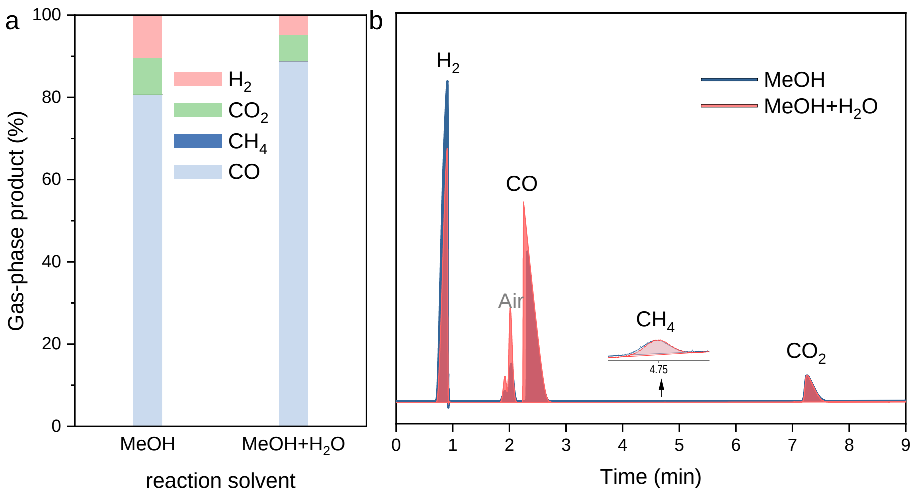


**Supplementary Figure 17 Identification and test of gas in carbonylation reaction.**

(a) Gas-phase composition. (b) Gas chromatogram. Reaction conditions: 30 mg 2 wt.% Ru/$NbO_x$, 0.2 mmol styrene, 2 mL MeOH (20 ul H2O), CO (1 bar), 160 °C, 10 h.

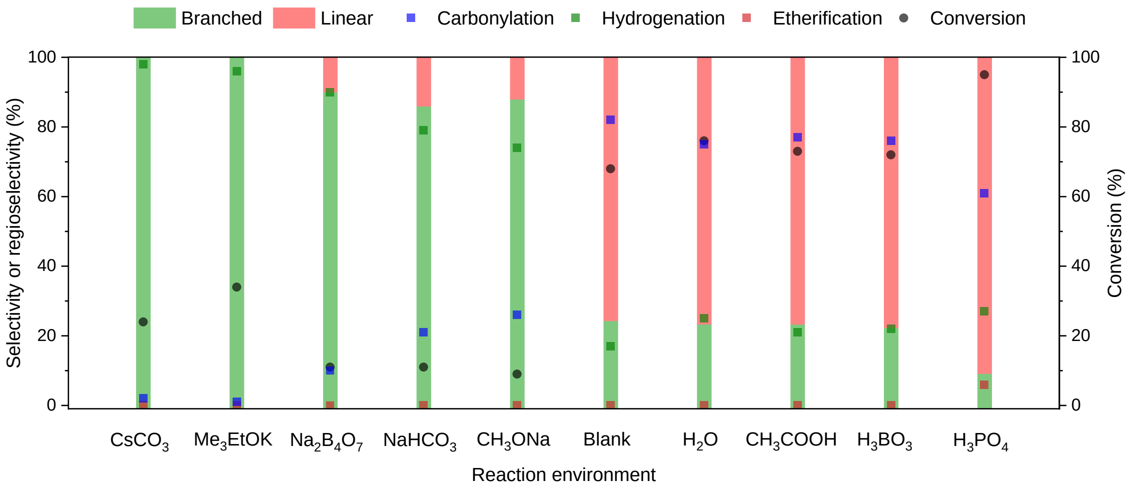


**Supplementary Figure 18 Regioselectivity regulation of methoxycarbonylation reactions on Ru/NbO$_x$.**

Reaction conditions: 2 wt.% Ru/NbO$_x$ (30 mg), styrene (2 mmol), 4 mmol MeOH (2 mL), additives (4 mmol), CO (1 bar), 170 °C, 10 h.

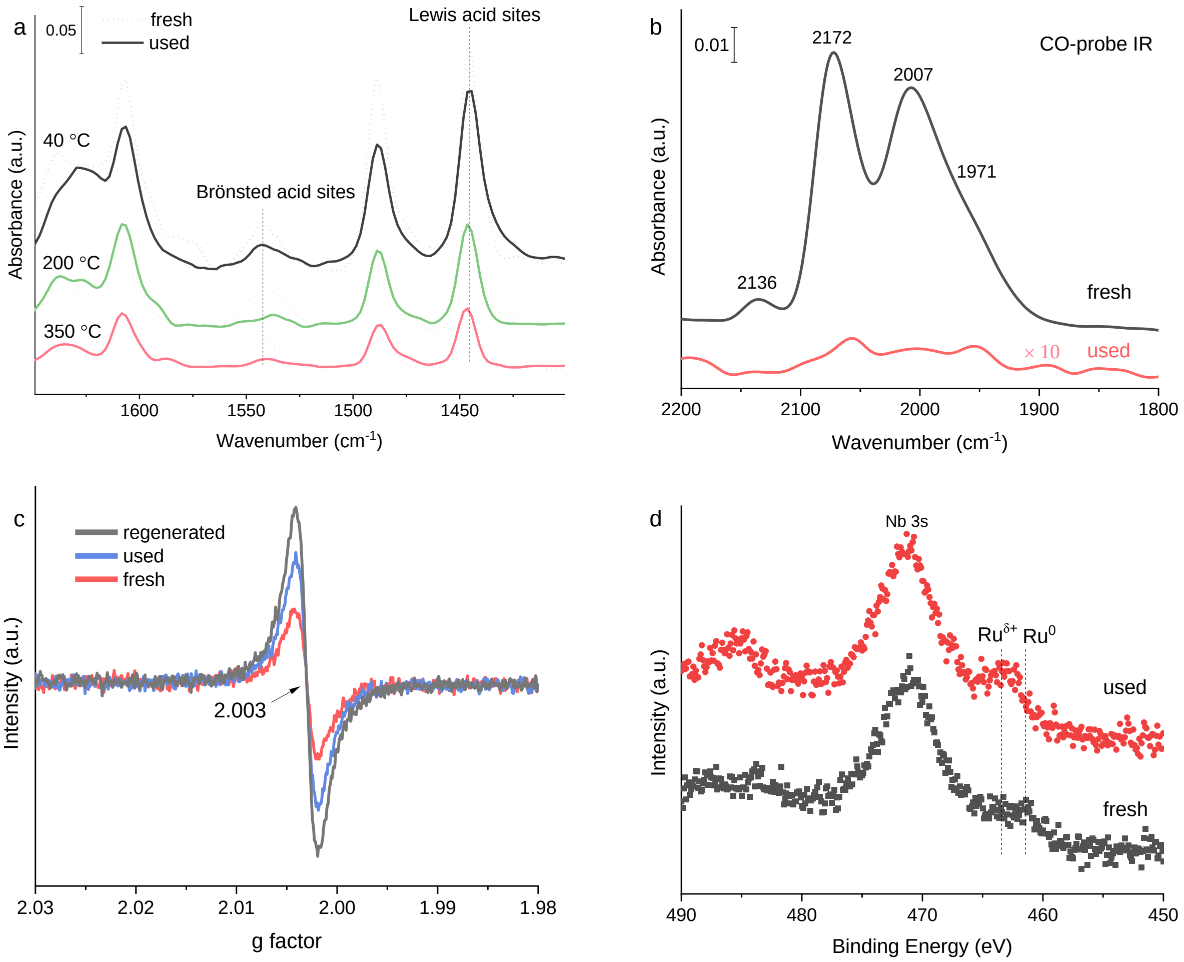


**Supplementary Figure 19 Characterizations of Ru/NbO$_x$ catalysts before and after carbonylation reaction.** (a) Pyridine-probe FTIR. (b) CO-probe FTIR. (c) EPR. (d) Ru 3*d* XPS.

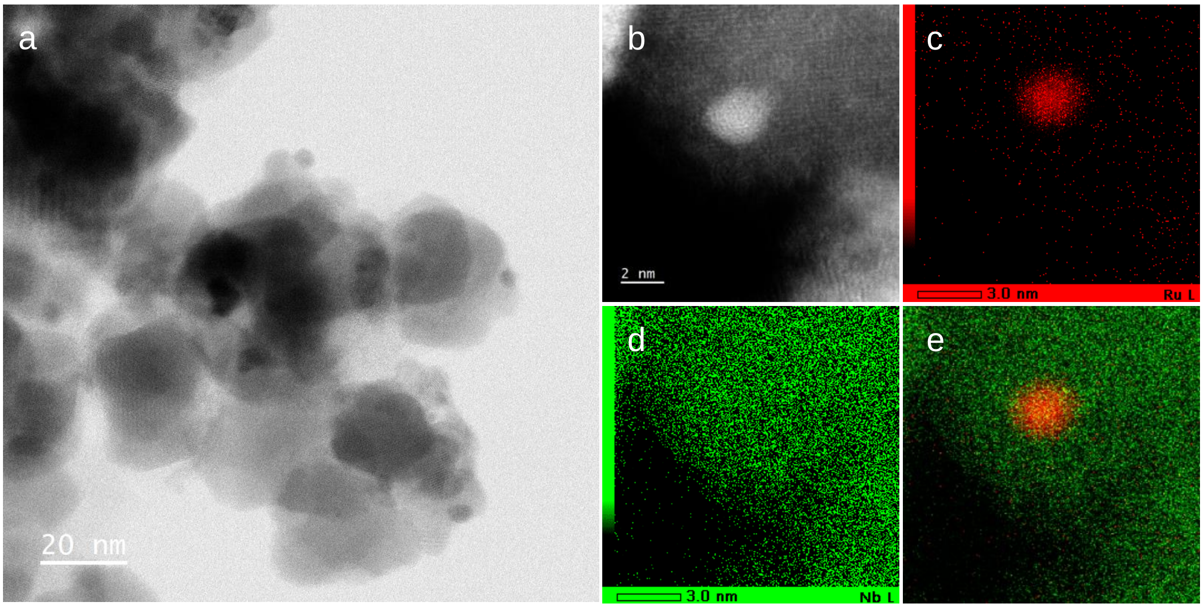


**Supplementary Figure 20 AC-HAADF STEM images and the corresponding Ru and Nb element map of the used 2 wt.% Ru/NbO$_x$.**

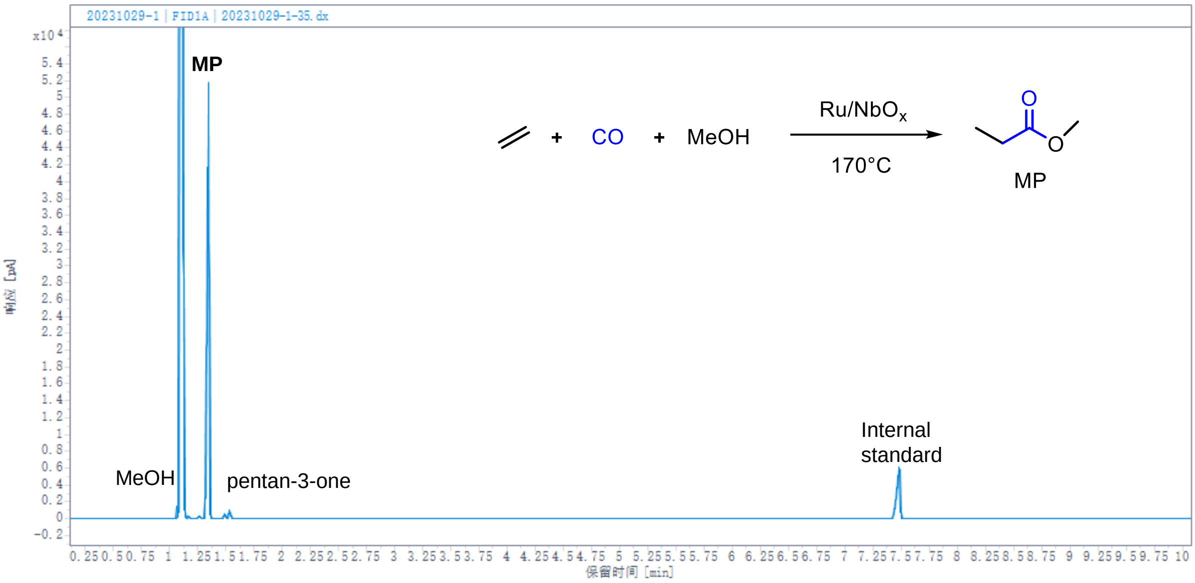


**Supplementary Figure 21 GC spectra of MP production from ethylene methoxycarbonylation in a scale-up run.**

Reaction conditions: 150 mg Ru/NbO$_x$, 4 umol $C_2H_4$ (6 bar), 8.5 umol CO (14 bar), 20 ul dodecane, 2 mL MeOH, 170°C, 10 h.

**Supplementary Note 7 The structural data of $Nb_2O_5$, $NbO_x$ and Ru/$NbO_x$ support, the dissociative adsorption process of methanol, and the hydromethoxycarbonylation process of styrene were attached. The details include the optimized xyz coordinates, the corresponding total energy, thermodynamic zero point energy (*ZPE*) correction, and the entropy contribution (*T*Δ*S*).**

$Nb_2O_5$: $CH_3OH$ adsorption
(*E* = -1140.9262 eV)
AutoCreatByScript: O Nb
1.00000000000000

| | | |
|---|---|---|
| 7.9332688600000001 | 0.0000006300000000 | 0.0000000000000000 |
| 0.0000000000000000 | 11.4522889699999997 | 0.0000000000000000 |
| 0.0000000000000000 | 0.0000000000000000 | 32.7181709299999994 |

O Nb
90 36
Selective dynamics
Direct

| | | |
|---|---|---|
| 0.4534627765720065 | 0.0949785566820023 | 0.0000000000000000 |
| 0.9534627765720097 | 0.0949785566820012 | 0.0000000000000000 |
| 0.4534627765720065 | 0.4283118900150074 | 0.0000000000000000 |
| 0.9534627765720097 | 0.4283118900150051 | 0.0000000000000000 |
| 0.4534627765720065 | 0.7616452233490068 | 0.0000000000000000 |
| 0.9534627765720097 | 0.7616452233489994 | 0.0000000000000000 |
| 0.1955712230439985 | 0.2616453474380011 | 0.0135137049989993 |
| 0.6955712230439985 | 0.2616453474380008 | 0.0135137049989993 |
| 0.1955712230439985 | 0.5949786807720082 | 0.0135137049989993 |
| 0.6955712230439985 | 0.5949786807720017 | 0.0135137049989993 |
| 0.1955712230439985 | 0.9283120141050134 | 0.0135137049989993 |
| 0.6955712230439985 | 0.9283120141050035 | 0.0135137049989993 |
| 0.4484906587389972 | 0.2616449757260033 | 0.0728553503239983 |
| 0.9484906587390037 | 0.2616449757260000 | 0.0728553503239983 |
| 0.4484906587389972 | 0.5949783090590017 | 0.0728553503239983 |
| 0.9484906587390037 | 0.5949783090590083 | 0.0728553503239983 |
| 0.4484906587389972 | 0.9283116423920035 | 0.0728553503239983 |
| 0.9484906587390037 | 0.9283116423920035 | 0.0728553503239983 |
| 0.2014097329349996 | 0.2616445452179974 | 0.1321970365760023 |
| 0.7014097329350044 | 0.2616445452180007 | 0.1321970365760023 |
| 0.2014097329349996 | 0.5949778785510057 | 0.1321970365760023 |
| 0.7014097329350044 | 0.5949778785509992 | 0.1321970365760023 |
| 0.2014097329349996 | 0.9283112118840073 | 0.1321970365760023 |
| 0.7014097329350044 | 0.9283112118840009 | 0.1321970365760023 |
| 0.4435183522890004 | 0.0949778074770009 | 0.1457107178800001 |
| 0.9435183522889974 | 0.0949778074770020 | 0.1457107178800001 |
| 0.4435183522890004 | 0.4283111408100057 | 0.1457107178800001 |
| 0.9435183522889974 | 0.4283111408100057 | 0.1457107178800001 |
| 0.4435183522890004 | 0.7616444741430077 | 0.1457107178800001 |
| 0.9435183522889974 | 0.7616444741430041 | 0.1457107178800001 |

| | | |
|---|---|---|
| 0.4392793396900064 | 0.2616444789110020 | 0.1979144111660034 |
| 0.9392793396900032 | 0.2616444789110020 | 0.1979144111660034 |
| 0.4392793396900064 | 0.5949778122439966 | 0.1979144111660034 |
| 0.9392793396900032 | 0.5949778122439966 | 0.1979144111660034 |
| 0.4392793396900064 | 0.9283111455780046 | 0.1979144111660034 |
| 0.9392793396900032 | 0.9283111455780046 | 0.1979144111660034 |
| 0.1813874112409989 | 0.0949778936079966 | 0.2114279908170005 |
| 0.6813874112409977 | 0.0949778936079977 | 0.2114279908170005 |
| 0.1813874112409989 | 0.4283112269410032 | 0.2114279908170005 |
| 0.6813874112409977 | 0.4283112269409983 | 0.2114279908170005 |
| 0.1813874112409989 | 0.7616445602750019 | 0.2114279908170005 |
| 0.6813874112409977 | 0.7616445602750019 | 0.2114279908170005 |
| 0.4343069956900042 | 0.0949774386079987 | 0.2707694632339979 |
| 0.9343069956900010 | 0.0949774386079987 | 0.2707694632339979 |
| 0.4343069956900042 | 0.4283107719410044 | 0.2707694632339979 |
| 0.9343069956900010 | 0.4283107719409998 | 0.2707694632339979 |
| 0.4343069956900042 | 0.7616441052750124 | 0.2707694632339979 |
| 0.9343069956900010 | 0.7616441052750088 | 0.2707694632339979 |
| 0.1931333422341817 | 0.0953909190457136 | 0.3303836680001395 |
| 0.6931393290047970 | 0.0955390480973493 | 0.3303722240464433 |
| 0.6931363662749562 | 0.4287124761239600 | 0.3303821382639223 |
| 0.1931280850034064 | 0.4289018522699089 | 0.3303894198117502 |
| 0.1931258870212754 | 0.7621454062644015 | 0.3303793669014862 |
| 0.6931329809726962 | 0.7621702948367827 | 0.3303702844526099 |
| 0.9353651974902187 | 0.2620071182150203 | 0.3451913276730020 |
| 0.4350548596356361 | 0.2620123508243664 | 0.3451874155763921 |
| 0.4353050825917123 | 0.5953435516739917 | 0.3451958024478267 |
| 0.9350972895875654 | 0.5953516051752001 | 0.3451914676691814 |
| 0.4352619040095059 | 0.9286843835455802 | 0.3451987960368796 |
| 0.9351494531570844 | 0.9286684266480182 | 0.3451915860542960 |
| 0.4320110407142365 | 0.0953357917086065 | 0.3968797735297834 |
| 0.9318765477955336 | 0.0953208941341097 | 0.3968721600619210 |
| 0.4319408951114188 | 0.4286593508840887 | 0.3968766954686998 |
| 0.9319330918089789 | 0.4286683627926317 | 0.3968776821538430 |
| 0.9319623940348881 | 0.7619954875837730 | 0.3968782534075556 |
| 0.4318976188839470 | 0.7620003648634074 | 0.3968774242916635 |
| 0.1734216550874398 | 0.2617711666759022 | 0.4115368922019293 |
| 0.6734110886716794 | 0.2618284946979861 | 0.4114937089505133 |
| 0.1734120740477011 | 0.5951130227874503 | 0.4115114220948593 |
| 0.6734058185867633 | 0.5951421696804881 | 0.4115485140269376 |
| 0.6734027379797095 | 0.9284286430941552 | 0.4115317247951497 |
| 0.1734187425136890 | 0.9285219663849881 | 0.4115282581651662 |
| 0.4256362781767872 | 0.2621208587633009 | 0.4715336185915799 |
| 0.9254798951832817 | 0.2620673587251856 | 0.4715310797903773 |

| | | |
|---|---|---|
| 0.4254844588619043 | 0.5954222782076862 | 0.4715356570948228 |
| 0.9256327314511008 | 0.5954796004674099 | 0.4715383393947027 |
| 0.9255252105953309 | 0.9287714296794908 | 0.4715419059129604 |
| 0.4255880249235065 | 0.9287793897277911 | 0.4715361908139508 |
| 0.9053295108040091 | 0.4287544791524617 | 0.5436354423839752 |
| 0.4053150954948626 | 0.0954267771506894 | 0.5436467381170766 |
| 0.9053518012716576 | 0.0954201444380374 | 0.5436574821820398 |
| 0.4053503939583061 | 0.4287518460836294 | 0.5436514955965771 |
| 0.4053441654788096 | 0.7620886601590460 | 0.5436396697873105 |
| 0.9053420873497257 | 0.7620930752315738 | 0.5436630708254500 |
| 0.6603859641051265 | 0.2620738306256559 | 0.5398235223838069 |
| 0.1603827204000494 | 0.2620906829102407 | 0.5397974685086144 |
| 0.6603900340759071 | 0.5954218117390122 | 0.5398071501078068 |
| 0.1603913502470372 | 0.5954152926091943 | 0.5398262849869044 |
| 0.1603888191402443 | 0.9287455730356814 | 0.5398082999929622 |
| 0.6603795679269031 | 0.9287582744050202 | 0.5398288023119390 |
| 0.4212494542350058 | 0.2616454108449986 | 0.0151065289700014 |
| 0.9212494542350093 | 0.2616454108449986 | 0.0151065289700014 |
| 0.4212494542350058 | 0.5949787441780003 | 0.0151065289700014 |
| 0.9212494542350093 | 0.5949787441780003 | 0.0151065289700014 |
| 0.4212494542350058 | 0.9283120775110072 | 0.0151065289700014 |
| 0.9212494542350093 | 0.9283120775110089 | 0.0151065289700014 |
| 0.4757319089200000 | 0.2616445689349973 | 0.1306041949610020 |
| 0.9757319089200033 | 0.2616445689349974 | 0.1306041949610020 |
| 0.4757319089200000 | 0.5949779022690094 | 0.1306041949610020 |
| 0.9757319089200033 | 0.5949779022690009 | 0.1306041949610020 |
| 0.4757319089200000 | 0.9283112356020069 | 0.1306041949610020 |
| 0.9757319089200033 | 0.9283112356020092 | 0.1306041949610020 |
| 0.4070655843149993 | 0.0949779281479989 | 0.2130205561419984 |
| 0.9070655843150029 | 0.0949779281479977 | 0.2130205561419984 |
| 0.4070655843149993 | 0.4283112614820013 | 0.2130205561419984 |
| 0.9070655843150029 | 0.4283112614820013 | 0.2130205561419984 |
| 0.4070655843149993 | 0.7616445948150098 | 0.2130205561419984 |
| 0.9070655843150029 | 0.7616445948150102 | 0.2130205561419984 |
| 0.9653223725684019 | 0.0953108215970485 | 0.3289725032015784 |
| 0.4653017838618465 | 0.0953381073037183 | 0.3289719692821170 |
| 0.4652996178734511 | 0.4286525643562009 | 0.3289721640923026 |
| 0.9653177900059943 | 0.4286713698467616 | 0.3289733406095800 |
| 0.4653027102944991 | 0.7620011378160395 | 0.3289723829004780 |
| 0.9653042045391568 | 0.7619921808051743 | 0.3289724723356092 |
| 0.4015044008330633 | 0.2620355010886776 | 0.4130542076792449 |
| 0.9015216967312509 | 0.2620323300451974 | 0.4130462778076836 |
| 0.4014924917231171 | 0.5953657707169493 | 0.4130500680660887 |
| 0.9015205926786316 | 0.5953739788703358 | 0.4130589915360232 |

| | | |
|---|---|---|
| 0.4015069503070589 | 0.9287112724966186 | 0.4130529212448273 |
| 0.9015133721560311 | 0.9286973606846664 | 0.4130590901896004 |
| 0.4312374794780725 | 0.2621845288550268 | 0.5297478852221488 |
| 0.9312145188887533 | 0.2621777833322589 | 0.5297433031987099 |
| 0.4312273824122727 | 0.5955123902469770 | 0.5297471145425419 |
| 0.9312395985052325 | 0.5955277535810783 | 0.5297512638973978 |
| 0.4312335149133767 | 0.9288538264692252 | 0.5297481415459849 |
| 0.9312206316086598 | 0.9288505033809509 | 0.5297544037119349 |

$NbO_x$

($E$ = -1131.9928 eV)

```
AutoCreatByScript: O Nb
   1.00000000000000
     7.9332688600000001    0.0000006300000000    0.0000000000000000
     0.0000000000000000   11.4522889699999997    0.0000000000000000
     0.0000000000000000    0.0000000000000000   32.7181709299999994
   O    Nb
    89    36
Selective dynamics
Direct
  0.4534627765720146  0.0949785566820024  0.0000000000000000
  0.9534627765720126  0.0949785566820012  0.0000000000000000
  0.4534627765720146  0.4283118900150100  0.0000000000000000
  0.9534627765720126  0.4283118900150030  0.0000000000000000
  0.4534627765720146  0.7616452233490305  0.0000000000000000
  0.9534627765720126  0.7616452233489994  0.0000000000000000
  0.1955712230439985  0.2616453474380008  0.0135137049989993
  0.6955712230439985  0.2616453474380017  0.0135137049989993
  0.1955712230439985  0.5949786807720315  0.0135137049989993
  0.6955712230439985  0.5949786807720017  0.0135137049989993
  0.1955712230439985  0.9283120141050357  0.0135137049989993
  0.6955712230439985  0.9283120141050035  0.0135137049989993
  0.4484906587389972  0.2616449757260111  0.0728553503239982
  0.9484906587390199  0.2616449757260000  0.0728553503239982
  0.4484906587389972  0.5949783090590017  0.0728553503239982
  0.9484906587390199  0.5949783090590316  0.0728553503239982
  0.4484906587389972  0.9283116423920035  0.0728553503239982
  0.9484906587390199  0.9283116423920035  0.0728553503239982
  0.2014097329349997  0.2616445452179974  0.1321970365760023
  0.7014097329350051  0.2616445452180088  0.1321970365760023
  0.2014097329349997  0.5949778785510290  0.1321970365760023
  0.7014097329350051  0.5949778785509992  0.1321970365760023
  0.2014097329349997  0.9283112118840395  0.1321970365760023
  0.7014097329350051  0.9283112118840009  0.1321970365760023
  0.4435183522890077  0.0949778074770009  0.1457107178800001
  0.9435183522889974  0.0949778074770021  0.1457107178800001
  0.4435183522890077  0.4283111408100136  0.1457107178800001
  0.9435183522889974  0.4283111408100143  0.1457107178800001
  0.4435183522890077  0.7616444741430443  0.1457107178800001
  0.9435183522889974  0.7616444741430216  0.1457107178800001
  0.4392793396900138  0.2616444789110020  0.1979144111660034
  0.9392793396900032  0.2616444789110020  0.1979144111660034
  0.4392793396900138  0.5949778122439966  0.1979144111660034
```

| | | |
|---|---|---|
| 0.9392793396900032 | 0.5949778122439966 | 0.1979144111660034 |
| 0.4392793396900138 | 0.9283111455780074 | 0.1979144111660034 |
| 0.9392793396900032 | 0.9283111455780004 | 0.1979144111660034 |
| 0.1813874112409989 | 0.0949778936079966 | 0.2114279908170005 |
| 0.6813874112409977 | 0.0949778936079979 | 0.2114279908170005 |
| 0.1813874112409989 | 0.4283112269410178 | 0.2114279908170005 |
| 0.6813874112409977 | 0.4283112269409983 | 0.2114279908170005 |
| 0.1813874112409989 | 0.7616445602750019 | 0.2114279908170005 |
| 0.6813874112409977 | 0.7616445602750019 | 0.2114279908170005 |
| 0.4343069956900116 | 0.0949774386079991 | 0.2707694632339977 |
| 0.9343069956900010 | 0.0949774386079989 | 0.2707694632339977 |
| 0.4343069956900116 | 0.4283107719410190 | 0.2707694632339977 |
| 0.9343069956900010 | 0.4283107719409998 | 0.2707694632339977 |
| 0.4343069956900116 | 0.7616441052750166 | 0.2707694632339977 |
| 0.9343069956900010 | 0.7616441052750106 | 0.2707694632339977 |
| 0.1893645798287616 | 0.0958876999596116 | 0.3305798563654579 |
| 0.6894421780900338 | 0.0957992537234093 | 0.3303318393395864 |
| 0.6898051389759928 | 0.4287264140704642 | 0.3302676763825102 |
| 0.1900384239136696 | 0.4250565989805027 | 0.3299886232331342 |
| 0.1899720056047489 | 0.7665242133364906 | 0.3300662451397820 |
| 0.6897400767381620 | 0.7628557763841951 | 0.3303148242497137 |
| 0.9312418849605459 | 0.2612387839255291 | 0.3469123822348867 |
| 0.4335768039700260 | 0.2613752195327312 | 0.3469777156698370 |
| 0.4274992706417635 | 0.5955735902340639 | 0.3449277142698403 |
| 0.9375547429349573 | 0.5956281363570769 | 0.3449195188442701 |
| 0.4334788141277865 | 0.9298610360794058 | 0.3469525892019441 |
| 0.9312527902808784 | 0.9300157416551481 | 0.3469013012855460 |
| 0.4286955113798663 | 0.0955817459953139 | 0.3983525315848318 |
| 0.9264952672047663 | 0.0956135649846032 | 0.3983029610964342 |
| 0.4249873956799820 | 0.4279778654442572 | 0.3968555806246147 |
| 0.9277335017991238 | 0.4279678813179956 | 0.3967342421538036 |
| 0.9278018613523110 | 0.7631986681313967 | 0.3967934331699053 |
| 0.4250414581735756 | 0.7631254697435800 | 0.3968885767936834 |
| 0.1710573905191125 | 0.2605838151810764 | 0.4161843119070851 |
| 0.6710954018322172 | 0.2619650913800050 | 0.4167698123736688 |
| 0.1687284213020165 | 0.5954777713482334 | 0.4155880196341638 |
| 0.6678469544908702 | 0.5955132345587373 | 0.4097656226649477 |
| 0.6710562680644198 | 0.9287771739149585 | 0.4166320940444032 |
| 0.1710330829631000 | 0.9302897800352795 | 0.4161312410574516 |
| 0.4218740773930665 | 0.2700700178017124 | 0.4744423530491425 |
| 0.9254164232383871 | 0.2713397089058466 | 0.4743052620365294 |
| 0.4366989019606384 | 0.5953496572031917 | 0.4713013734957621 |
| 0.8985800471159362 | 0.5952821059458480 | 0.4713683018460023 |
| 0.9252008012816223 | 0.9209051086022026 | 0.4742794491802828 |

| | | |
|---|---|---|
| 0.4220212337279402 | 0.9220609936280434 | 0.4744132601107668 |
| 0.8846522092169541 | 0.4434881186041135 | 0.5469940300425259 |
| 0.4053276220348151 | 0.0957885343888795 | 0.5410270002984959 |
| 0.9349853124637389 | 0.0958049354465301 | 0.5405764178929383 |
| 0.4531000093307607 | 0.4444113349414109 | 0.5474778331252633 |
| 0.4528820968336695 | 0.7469948351771076 | 0.5473484161566184 |
| 0.8852785841223466 | 0.7479164001654476 | 0.5468796273029100 |
| 0.6690585483179422 | 0.2589048385168563 | 0.5406695161155872 |
| 0.1684759085747442 | 0.2845601193657021 | 0.5417494819127808 |
| 0.1681558226233475 | 0.5951913750113967 | 0.5319478134192680 |
| 0.1685627168723375 | 0.9071332048885054 | 0.5416577944838608 |
| 0.6692015269887857 | 0.9325272088801996 | 0.5407946561577163 |
| 0.4212494542350133 | 0.2616454108449986 | 0.0151065289700014 |
| 0.9212494542350100 | 0.2616454108449986 | 0.0151065289700014 |
| 0.4212494542350133 | 0.5949787441780003 | 0.0151065289700014 |
| 0.9212494542350100 | 0.5949787441780003 | 0.0151065289700014 |
| 0.4212494542350133 | 0.9283120775110093 | 0.0151065289700014 |
| 0.9212494542350100 | 0.9283120775110333 | 0.0151065289700014 |
| 0.4757319089200072 | 0.2616445689349973 | 0.1306041949610020 |
| 0.9757319089200038 | 0.2616445689349974 | 0.1306041949610020 |
| 0.4757319089200072 | 0.5949779022690308 | 0.1306041949610020 |
| 0.9757319089200038 | 0.5949779022690009 | 0.1306041949610020 |
| 0.4757319089200072 | 0.9283112356020100 | 0.1306041949610020 |
| 0.9757319089200038 | 0.9283112356020100 | 0.1306041949610020 |
| 0.4070655843149993 | 0.0949779281479989 | 0.2130205561419984 |
| 0.9070655843150148 | 0.0949779281479977 | 0.2130205561419984 |
| 0.4070655843149993 | 0.4283112614820013 | 0.2130205561419984 |
| 0.9070655843150148 | 0.4283112614820013 | 0.2130205561419984 |
| 0.4070655843149993 | 0.7616445948150270 | 0.2130205561419984 |
| 0.9070655843150148 | 0.7616445948150344 | 0.2130205561419984 |
| 0.9597839956166531 | 0.0956041539306551 | 0.3290718641724757 |
| 0.4600014180981259 | 0.0955721836022683 | 0.3290706806614705 |
| 0.4605335384564537 | 0.4283998527061810 | 0.3289464210643628 |
| 0.9610028512639304 | 0.4279323337410601 | 0.3288912379892048 |
| 0.4604522793206943 | 0.7627018150146050 | 0.3289512950378570 |
| 0.9609694380012411 | 0.7632378957565441 | 0.3288899594338667 |
| 0.4010674391902848 | 0.2623856145117418 | 0.4137061824993613 |
| 0.9010786989763758 | 0.2626088737758430 | 0.4136691268615971 |
| 0.4014057953767195 | 0.5955600995456845 | 0.4119733698673949 |
| 0.8995212004525246 | 0.5956026125477817 | 0.4114044868449255 |
| 0.4010247033504691 | 0.9288089519411082 | 0.4136851744308769 |
| 0.9010400763957985 | 0.9286598297297435 | 0.4136423919094541 |
| 0.4276805098448102 | 0.2643331295356329 | 0.5310324371441653 |
| 0.9263134491576186 | 0.2651156700556734 | 0.5308898649407960 |

| | | |
|---|---|---|
| 0.4036562053951772 | 0.5956327263424581 | 0.5278451831871329 |
| 0.9327114606314686 | 0.5956020517383172 | 0.5277171624294643 |
| 0.4278198259772953 | 0.9274516355106657 | 0.5310321988792994 |
| 0.9264149404943691 | 0.9266362120445693 | 0.5308778892784752 |

$Nb_2O_5$: $CH_3OH$ adsorption

($E$ = -1172.0421 eV, ZPE = 1.427442046 eV, $S*T$ = 0.38777092 eV)

AutoCreatByScript: H C O Nb

1.00000000000000

| | | |
|---|---|---|
| 7.9332688600000001 | 0.0000006300000000 | 0.0000000000000000 |
| 0.0000000000000000 | 11.4522889699999997 | 0.0000000000000000 |
| 0.0000000000000000 | 0.0000000000000000 | 32.7181709299999994 |

| H | C | O | Nb |
|---|---|---|---|
| 4 | 1 | 91 | 36 |

Selective dynamics

Direct

| | | |
|---|---|---|
| 0.6683632886861002 | 0.6541850176669897 | 0.6126922712979506 |
| 0.3547546289342547 | 0.5315775403293933 | 0.6092050456562147 |
| 0.6437372989706355 | 0.4990883582963614 | 0.6156644290541418 |
| 0.5704098475698549 | 0.5914038770250771 | 0.6570670484314570 |
| 0.5874879777204124 | 0.5836375453571473 | 0.6238738858777041 |
| 0.4534627765720095 | 0.0949785566820042 | 0.0000000000000000 |
| 0.9534627765720175 | 0.0949785566820012 | 0.0000000000000000 |
| 0.4534627765720095 | 0.4283118900150107 | 0.0000000000000000 |
| 0.9534627765720175 | 0.4283118900150030 | 0.0000000000000000 |
| 0.4534627765720095 | 0.7616452233490214 | 0.0000000000000000 |
| 0.9534627765720175 | 0.7616452233489994 | 0.0000000000000000 |
| 0.1955712230439985 | 0.2616453474380037 | 0.0135137049989993 |
| 0.6955712230439985 | 0.2616453474380057 | 0.0135137049989993 |
| 0.1955712230439985 | 0.5949786807720169 | 0.0135137049989993 |
| 0.6955712230439985 | 0.5949786807720017 | 0.0135137049989993 |
| 0.1955712230439985 | 0.9283120141050208 | 0.0135137049989993 |
| 0.6955712230439985 | 0.9283120141050035 | 0.0135137049989993 |
| 0.4484906587389972 | 0.2616449757260075 | 0.0728553503239982 |
| 0.9484906587390119 | 0.2616449757260000 | 0.0728553503239982 |
| 0.4484906587389972 | 0.5949783090590017 | 0.0728553503239982 |
| 0.9484906587390119 | 0.5949783090590169 | 0.0728553503239982 |
| 0.4484906587389972 | 0.9283116423920035 | 0.0728553503239982 |
| 0.9484906587390119 | 0.9283116423920035 | 0.0728553503239982 |
| 0.2014097329350003 | 0.2616445452179974 | 0.1321970365760023 |
| 0.7014097329350062 | 0.2616445452180050 | 0.1321970365760023 |
| 0.2014097329350003 | 0.5949778785510144 | 0.1321970365760023 |
| 0.7014097329350062 | 0.5949778785509992 | 0.1321970365760023 |
| 0.2014097329350003 | 0.9283112118840239 | 0.1321970365760023 |
| 0.7014097329350062 | 0.9283112118840009 | 0.1321970365760023 |
| 0.4435183522890019 | 0.0949778074770009 | 0.1457107178800001 |
| 0.9435183522889974 | 0.0949778074770039 | 0.1457107178800001 |
| 0.4435183522890019 | 0.4283111408100105 | 0.1457107178800001 |
| 0.9435183522889974 | 0.4283111408100120 | 0.1457107178800001 |

| | | |
|---|---|---|
| 0.4435183522890019 | 0.7616444741430167 | 0.1457107178800001 |
| 0.9435183522889974 | 0.7616444741430166 | 0.1457107178800001 |
| 0.4392793396900079 | 0.2616444789110020 | 0.1979144111660034 |
| 0.9392793396900032 | 0.2616444789110020 | 0.1979144111660034 |
| 0.4392793396900079 | 0.5949778122439966 | 0.1979144111660034 |
| 0.9392793396900032 | 0.5949778122439966 | 0.1979144111660034 |
| 0.4392793396900079 | 0.9283111455780004 | 0.1979144111660034 |
| 0.9392793396900032 | 0.9283111455780004 | 0.1979144111660034 |
| 0.1813874112409991 | 0.0949778936079966 | 0.2114279908170005 |
| 0.6813874112409977 | 0.0949778936079988 | 0.2114279908170005 |
| 0.1813874112409991 | 0.4283112269410086 | 0.2114279908170005 |
| 0.6813874112409977 | 0.4283112269409983 | 0.2114279908170005 |
| 0.1813874112409991 | 0.7616445602750019 | 0.2114279908170005 |
| 0.6813874112409977 | 0.7616445602750019 | 0.2114279908170005 |
| 0.4343069956900057 | 0.0949774386079999 | 0.2707694632339976 |
| 0.9343069956900010 | 0.0949774386079998 | 0.2707694632339976 |
| 0.4343069956900057 | 0.4283107719410092 | 0.2707694632339976 |
| 0.9343069956900010 | 0.4283107719409998 | 0.2707694632339976 |
| 0.4343069956900057 | 0.7616441052750190 | 0.2707694632339976 |
| 0.9343069956900010 | 0.7616441052750184 | 0.2707694632339976 |
| 0.1902362427229229 | 0.0954532789783692 | 0.3298525621998628 |
| 0.6903686059411301 | 0.0956042140423699 | 0.3302929787882552 |
| 0.6903118769560996 | 0.4260780351561666 | 0.3299761767423248 |
| 0.1898573368748547 | 0.4301628311343137 | 0.3302441706583429 |
| 0.1898549264728025 | 0.7616912737452681 | 0.3303151254840756 |
| 0.6900642709343842 | 0.7657038404042532 | 0.3300322264184726 |
| 0.9343618279069543 | 0.2618057331438108 | 0.3459349968957766 |
| 0.4311430764485301 | 0.2622673980065948 | 0.3457052756327870 |
| 0.4370183171829637 | 0.5957501510531701 | 0.3453256580467637 |
| 0.9276785218636713 | 0.5953716894837372 | 0.3454057370200821 |
| 0.4310956273263812 | 0.9290145880672327 | 0.3460263850447153 |
| 0.9344449493209489 | 0.9288456967757293 | 0.3460091967307391 |
| 0.4275937777560369 | 0.0961300565228757 | 0.3967746894184958 |
| 0.9312253321071069 | 0.0952177392476657 | 0.3975844370824233 |
| 0.4304617798973011 | 0.4289727985592993 | 0.3972980246214299 |
| 0.9275541860658861 | 0.4286934030785230 | 0.3971613229764273 |
| 0.9277561770799280 | 0.7619687136210436 | 0.3971549191815975 |
| 0.4305778339153211 | 0.7622065459408165 | 0.3979597370422716 |
| 0.1715315092028192 | 0.2628954048518085 | 0.4135375168809575 |
| 0.6718011772123867 | 0.2604732160904543 | 0.4127610929157456 |
| 0.1705457665655830 | 0.5955451051576799 | 0.4098055472476345 |
| 0.6712471803806840 | 0.5955661163916550 | 0.4144777993573808 |
| 0.6722410648857376 | 0.9289185639017115 | 0.4127899379031101 |
| 0.1718630162086195 | 0.9268833005130442 | 0.4139690223535388 |

| | | |
|---|---|---|
| 0.4268014381203725 | 0.2615435233573730 | 0.4722834271127427 |
| 0.9233767366681170 | 0.2639697753029762 | 0.4724348391071887 |
| 0.4087576078361747 | 0.5912549532763874 | 0.4727821951874550 |
| 0.9358196106453515 | 0.5951252406100865 | 0.4719441782392381 |
| 0.9227187330778728 | 0.9266947996253241 | 0.4726636406275163 |
| 0.4293184999080540 | 0.9426633419983017 | 0.4719404382645068 |
| 0.9056481873159843 | 0.4300321241265932 | 0.5455633650764826 |
| 0.4047503193137125 | 0.0980077268566682 | 0.5470425779275234 |
| 0.9102189423879089 | 0.0954530217672217 | 0.5442610660028374 |
| 0.4083861188519219 | 0.4261020847848022 | 0.5449657503820067 |
| 0.4095850893526228 | 0.7663823837922052 | 0.5396911482479791 |
| 0.9061885008144211 | 0.7612442894539397 | 0.5455153350413244 |
| 0.6636255794934641 | 0.2610526615574305 | 0.5409387339803838 |
| 0.1612522673418514 | 0.2636932341005487 | 0.5402557678155272 |
| 0.6633378071654843 | 0.5958965329063364 | 0.5362997440134681 |
| 0.1614617124657457 | 0.5947992409683392 | 0.5428535218408127 |
| 0.1628581989915573 | 0.9283030352868852 | 0.5400730567207693 |
| 0.6624575459055326 | 0.9317948334369468 | 0.5413952013476314 |
| 0.4264289120985675 | 0.5989641620106841 | 0.6035870727470072 |
| 0.4212494542350074 | 0.2616454108449986 | 0.0151065289700014 |
| 0.9212494542350144 | 0.2616454108449986 | 0.0151065289700014 |
| 0.4212494542350074 | 0.5949787441780003 | 0.0151065289700014 |
| 0.9212494542350144 | 0.5949787441780003 | 0.0151065289700014 |
| 0.4212494542350074 | 0.9283120775110023 | 0.0151065289700014 |
| 0.9212494542350144 | 0.9283120775110212 | 0.0151065289700014 |
| 0.4757319089200030 | 0.2616445689349973 | 0.1306041949610020 |
| 0.9757319089200063 | 0.2616445689349974 | 0.1306041949610020 |
| 0.4757319089200030 | 0.5949779022690176 | 0.1306041949610020 |
| 0.9757319089200063 | 0.5949779022690009 | 0.1306041949610020 |
| 0.4757319089200030 | 0.9283112356020153 | 0.1306041949610020 |
| 0.9757319089200063 | 0.9283112356020206 | 0.1306041949610020 |
| 0.4070655843149993 | 0.0949779281480000 | 0.2130205561419984 |
| 0.9070655843150041 | 0.0949779281479977 | 0.2130205561419984 |
| 0.4070655843149993 | 0.4283112614820013 | 0.2130205561419984 |
| 0.9070655843150041 | 0.4283112614820013 | 0.2130205561419984 |
| 0.4070655843149993 | 0.7616445948150223 | 0.2130205561419984 |
| 0.9070655843150041 | 0.7616445948150239 | 0.2130205561419984 |
| 0.9622593748702578 | 0.0951805634258557 | 0.3291662582067964 |
| 0.4620457379875957 | 0.0957649128981415 | 0.3292256900988436 |
| 0.4622416466196588 | 0.4292194641413382 | 0.3292189345034044 |
| 0.9617722221021139 | 0.4286946784886038 | 0.3291634534170082 |
| 0.4620155979192443 | 0.7618290280591540 | 0.3292788655400225 |
| 0.9616636497596621 | 0.7618419816874420 | 0.3291853097689449 |
| 0.3998631460659697 | 0.2628564483289935 | 0.4137786365857635 |

| | | |
|---|---|---|
| 0.8999119017137147 | 0.2620672103604114 | 0.4137513982548521 |
| 0.3981185756734907 | 0.5954195513579796 | 0.4148577456291584 |
| 0.8998671749000459 | 0.5954405447952436 | 0.4136815494240702 |
| 0.4002165566459555 | 0.9296871255422474 | 0.4135337449390051 |
| 0.9002551305425769 | 0.9286749441463480 | 0.4138791681051558 |
| 0.4336807226136358 | 0.2637762924644826 | 0.5304715837789026 |
| 0.9318936386437282 | 0.2625224565094419 | 0.5304979443444415 |
| 0.4311781425414496 | 0.5991181750707976 | 0.5321259237175366 |
| 0.9343620617517251 | 0.5951937150783401 | 0.5300860571281568 |
| 0.4339822725848162 | 0.9358118547517500 | 0.5298746590436116 |
| 0.9329418192044455 | 0.9281936318870887 | 0.5307060256311408 |

$Nb_2O_5$: Transition state for $CH_3OH$ dissociation
($E$ = -1171.7298 eV, ZPE = 1.278640088 eV, $S*T$ = 0.417567218 eV)
AutoCreatByScript: H C O Nb
1.00000000000000
7.9332688600000001 0.0000006300000000 0.0000000000000000
0.0000000000000000 11.4522889699999997 0.0000000000000000
0.0000000000000000 0.0000000000000000 32.7181709299999994
H C O Nb
4 1 91 36
Selective dynamics
Direct
0.2357514577214416 0.6029354398690020 0.5848406268421695
0.5917400719386553 0.6115255726669862 0.6330364393783637
0.4906402770118340 0.4738542857850604 0.6246002701502898
0.3981244914722071 0.5793070997487680 0.6587529065470580
0.4688175469813988 0.5675585475689855 0.6301481697618150
0.4534627765720079 0.0949785566820325 1.0000000000000000
-0.0465372234279875 0.0949785566820012 1.0000000000000000
0.4534627765720079 0.4283118900150047 1.0000000000000000
-0.0465372234279744 0.4283118900150032 1.0000000000000000
0.4534627765720079 0.7616452233492312 1.0000000000000000
-0.0465372234279744 0.7616452233491414 1.0000000000000000
0.1955712230439985 0.2616453474379983 1.0135137049990011
0.6955712230439985 0.2616453474379983 1.0135137049990011
0.1955712230439985 0.5949786807721748 1.0135137049990011
0.6955712230439985 0.5949786807720017 1.0135137049990011
0.1955712230439985 0.9283120141052881 1.0135137049990011
0.6955712230439985 0.9283120141050035 1.0135137049990011
0.4484906587389972 0.2616449757260013 1.0728553503239988
-0.0515093412609511 0.2616449757260002 1.0728553503239988
0.4484906587389972 0.5949783090590017 1.0728553503239988
-0.0515093412609524 0.5949783090591024 1.0728553503239988
0.4484906587389972 0.9283116423920035 1.0728553503239988
-0.0515093412609524 0.9283116423920038 1.0728553503239988
0.2014097329349994 0.2616445452179974 0.1321970365760023
0.7014097329350044 0.2616445452180839 0.1321970365760023
0.2014097329349994 0.5949778785511722 0.1321970365760023
0.7014097329350044 0.5949778785509992 0.1321970365760023
0.2014097329349994 0.9283112118842010 0.1321970365760023
0.7014097329350044 0.9283112118840009 0.1321970365760023
0.4435183522890007 0.0949778074770009 0.1457107178800001
-0.0564816477110023 0.0949778074770174 0.1457107178800001
0.4435183522890007 0.4283111408100028 0.1457107178800001
-0.0564816477110023 0.4283111408100028 0.1457107178800001

| | | |
|---|---|---|
| 0.4435183522890007 | 0.7616444741432578 | 0.1457107178800001 |
| -0.0564816477110023 | 0.7616444741431428 | 0.1457107178800001 |
| 0.4392793396900061 | 0.2616444789110020 | 0.1979144111660034 |
| -0.0607206603099968 | 0.2616444789110020 | 0.1979144111660034 |
| 0.4392793396900061 | 0.5949778122439966 | 0.1979144111660034 |
| -0.0607206603099968 | 0.5949778122441510 | 0.1979144111660034 |
| 0.4392793396900061 | 0.9283111455780004 | 0.1979144111660034 |
| -0.0607206603099968 | 0.9283111455780004 | 0.1979144111660034 |
| 0.1813874112409984 | 0.0949778936079966 | 0.2114279908170005 |
| 0.6813874112409977 | 0.0949778936080203 | 0.2114279908170005 |
| 0.1813874112409984 | 0.4283112269411141 | 0.2114279908170005 |
| 0.6813874112409977 | 0.4283112269409983 | 0.2114279908170005 |
| 0.1813874112409984 | 0.7616445602750019 | 0.2114279908170005 |
| 0.6813874112409977 | 0.7616445602750019 | 0.2114279908170005 |
| 0.4343069956900041 | 0.0949774386079981 | 0.2707694632339970 |
| -0.0656930043099990 | 0.0949774386079981 | 0.2707694632339970 |
| 0.4343069956900041 | 0.4283107719411249 | 0.2707694632339970 |
| -0.0656930043099990 | 0.4283107719409998 | 0.2707694632339970 |
| 0.4343069956900041 | 0.7616441052750063 | 0.2707694632339970 |
| -0.0656930043099990 | 0.7616441052751846 | 0.2707694632339970 |
| 0.1897567780879993 | 0.0966683991521332 | 0.3296150738215803 |
| 0.6898838359537163 | 0.0963879454491281 | 0.3304908206703812 |
| 0.6900004425116620 | 0.4281478238518736 | 0.3301120959235525 |
| 0.1891634192483040 | 0.4336849395131672 | 0.3305900139464495 |
| 0.1891544482747437 | 0.7605246504683182 | 0.3306079650199117 |
| 0.6896586290703336 | 0.7664551708883417 | 0.3302316668410925 |
| 0.4384599705660133 | 0.5966984833321745 | 0.3458840059108206 |
| -0.0734955222389754 | 0.5963806479959091 | 0.3465936439524668 |
| 0.4299829564583151 | 0.2638305327832416 | 0.3453531694141185 |
| -0.0655615371277047 | 0.2633816732375147 | 0.3456260572479631 |
| -0.0646608555399512 | 0.9291153867939315 | 0.3457731334904228 |
| 0.4293164281316915 | 0.9292438533485721 | 0.3457491073198386 |
| 0.4249965446610594 | 0.0972043475608557 | 0.3959415974008405 |
| -0.0716163172025631 | 0.7625129706531965 | 0.3976858561036512 |
| -0.0727250956368505 | 0.4300492580386397 | 0.3976570696203217 |
| -0.0674274150846849 | 0.0961278571122493 | 0.3969637670129839 |
| 0.4329825004798569 | 0.4303278234580351 | 0.3976797772175428 |
| 0.4320787583036544 | 0.7622855503932493 | 0.3985757066753665 |
| 0.1741491461748879 | 0.5956856578206935 | 0.4118216625096686 |
| 0.6715845452117838 | 0.2605783244356622 | 0.4119270032875927 |
| 0.6723810474656032 | 0.9298471926589559 | 0.4117321766872661 |
| 0.1711920977185090 | 0.2656109706532601 | 0.4130902822584998 |
| 0.1714866836474511 | 0.9261318093885206 | 0.4140068801682304 |
| 0.6763027802927200 | 0.5963407664301668 | 0.4176052266564164 |

| | | |
|---|---|---|
| 0.4330372272918441 | 0.9516100960616927 | 0.4707394590481896 |
| 0.4277464658967318 | 0.2584726437676197 | 0.4716622921800290 |
| -0.0528484414304320 | 0.5981482166779049 | 0.4738422214924774 |
| -0.0772823391085438 | 0.2593811568915336 | 0.4719107154757495 |
| -0.0790068844968257 | 0.9349078364452831 | 0.4723072971510907 |
| 0.4139144058730588 | 0.5903788292123633 | 0.4744020773067254 |
| 0.6740924342054923 | 0.6015085331939989 | 0.5430857854409360 |
| 0.4052618976395745 | 0.7690442098594310 | 0.5324800169627535 |
| 0.4115967399125808 | 0.4309613749042785 | 0.5419092526196853 |
| 0.1631133324636367 | 0.9331878051477185 | 0.5397604068981899 |
| 0.1614351963203590 | 0.2679440588883198 | 0.5397305201250877 |
| 0.6626585580756633 | 0.2636925215882875 | 0.5405644803282136 |
| 0.6605405857601320 | 0.9340890963648377 | 0.5413107699621721 |
| -0.0920788865876865 | 0.7671238358970410 | 0.5429280302988848 |
| -0.0915373527051535 | 0.0988998666129179 | 0.5457943856150453 |
| -0.0966301811303740 | 0.4310544293117669 | 0.5422710197021948 |
| 0.4028764192587966 | 0.1014446578812473 | 0.5464123432508667 |
| 0.1481378262327516 | 0.5972012343847685 | 0.5547490731821663 |
| 0.3798187775572191 | 0.6208200386410425 | 0.5973145598822123 |
| 0.4212494542350055 | 0.2616454108449986 | 1.0151065289700014 |
| -0.0787505457649912 | 0.2616454108450635 | 1.0151065289700014 |
| -0.0787505457649912 | 0.5949787441780003 | 1.0151065289700014 |
| 0.4212494542350055 | 0.5949787441780003 | 1.0151065289700014 |
| 0.4212494542350055 | 0.9283120775110023 | 1.0151065289700014 |
| -0.0787505457649912 | 0.9283120775112003 | 1.0151065289700014 |
| 0.4757319089200003 | 0.2616445689349973 | 0.1306041949610020 |
| -0.0242680910799963 | 0.2616445689349974 | 0.1306041949610020 |
| 0.4757319089200003 | 0.5949779022691092 | 0.1306041949610020 |
| -0.0242680910799963 | 0.5949779022690010 | 0.1306041949610020 |
| 0.4757319089200003 | 0.9283112356020030 | 0.1306041949610020 |
| -0.0242680910799963 | 0.9283112356020033 | 0.1306041949610020 |
| 0.4070655843149993 | 0.0949779281480307 | 0.2130205561419984 |
| -0.0929344156849991 | 0.0949779281479977 | 0.2130205561419984 |
| 0.4070655843149993 | 0.4283112614820013 | 0.2130205561419984 |
| -0.0929344156849991 | 0.4283112614820945 | 0.2130205561419984 |
| 0.4070655843149993 | 0.7616445948152319 | 0.2130205561419984 |
| -0.0929344156849991 | 0.7616445948151462 | 0.2130205561419984 |
| -0.0379858937525760 | 0.0959445897751670 | 0.3290803156944559 |
| -0.0390368692715989 | 0.4301174330930202 | 0.3292250884788574 |
| -0.0391740885105694 | 0.7621454419189981 | 0.3292444048747328 |
| 0.4615578929881132 | 0.0966314615231657 | 0.3291923213714812 |
| 0.4617580323177460 | 0.4308614412095156 | 0.3292780089932731 |
| 0.4613783425921480 | 0.7617332767655398 | 0.3293501007584864 |
| -0.0950361770474384 | 0.5965696794141305 | 0.4147130768070018 |

| | | |
|---|---|---|
| 0.3998016438880335 | 0.9305029528098433 | 0.4129506567773879 |
| 0.3994486584030449 | 0.2638282046456047 | 0.4133106280892211 |
| -0.1004533008512764 | 0.2626175790832346 | 0.4133637633161651 |
| -0.0998973475324027 | 0.9301435796058458 | 0.4136200359145682 |
| 0.4024567608834510 | 0.5959586201379451 | 0.4167265051738515 |
| -0.0697618676060640 | 0.5979420569664824 | 0.5314759741280933 |
| 0.4338482883063595 | 0.9375875892908353 | 0.5284239804992540 |
| 0.4337507237997196 | 0.2634536117609269 | 0.5297108160217925 |
| -0.0677610150155126 | 0.2630021891372387 | 0.5299417969574616 |
| -0.0665970168929433 | 0.9331378601627751 | 0.5303257007494466 |
| 0.4452626130239641 | 0.5998572764867299 | 0.5347736173461051 |

$Nb_2O_5$: Final state for $CH_3OH$ dissociation

($E$ = -1172.3475 eV, ZPE = 1.411184356 eV, $S*T$ = 0.375869317 eV)

AutoCreatByScript: H C O Nb

1.00000000000000

| | | |
|---|---|---|
| 7.9332688600000001 | 0.0000006300000000 | 0.0000000000000000 |
| 0.0000000000000000 | 11.4522889699999997 | 0.0000000000000000 |
| 0.0000000000000000 | 0.0000000000000000 | 32.7181709299999994 |

| H | C | O | Nb |
|---|---|---|---|
| 4 | 1 | 91 | 36 |

Selective dynamics

Direct

| | | |
|---|---|---|
| 0.1498762027505622 | 0.4931476594002956 | 0.5571926471509011 |
| 0.7095990660996188 | 0.6467748933438060 | 0.6129531222673711 |
| 0.6564005261221197 | 0.4947103487054332 | 0.6180954391441064 |
| 0.5726820973889472 | 0.6005916027812511 | 0.6533281244220126 |
| 0.6086139169114440 | 0.5846902061465241 | 0.6213220366466812 |
| 0.4534627765720093 | 0.0949785566820042 | 0.0000000000000000 |
| 0.9534627765720113 | 0.0949785566820012 | 0.0000000000000000 |
| 0.4534627765720093 | 0.4283118900150115 | 0.0000000000000000 |
| 0.9534627765720113 | 0.4283118900150059 | 0.0000000000000000 |
| 0.4534627765720093 | 0.7616452233490222 | 0.0000000000000000 |
| 0.9534627765720113 | 0.7616452233489994 | 0.0000000000000000 |
| 0.1955712230439985 | 0.2616453474380004 | 0.0135137049989993 |
| 0.6955712230439985 | 0.2616453474380082 | 0.0135137049989993 |
| 0.1955712230439985 | 0.5949786807720187 | 0.0135137049989993 |
| 0.6955712230439985 | 0.5949786807720017 | 0.0135137049989993 |
| 0.1955712230439985 | 0.9283120141050263 | 0.0135137049989993 |
| 0.6955712230439985 | 0.9283120141050035 | 0.0135137049989993 |
| 0.4484906587389972 | 0.2616449757260066 | 0.0728553503239981 |
| 0.9484906587390095 | 0.2616449757260000 | 0.0728553503239981 |
| 0.4484906587389972 | 0.5949783090590017 | 0.0728553503239981 |
| 0.9484906587390095 | 0.5949783090590187 | 0.0728553503239981 |
| 0.4484906587389972 | 0.9283116423920035 | 0.0728553503239981 |
| 0.9484906587390095 | 0.9283116423920035 | 0.0728553503239981 |
| 0.2014097329349997 | 0.2616445452179974 | 0.1321970365760023 |
| 0.7014097329350055 | 0.2616445452180059 | 0.1321970365760023 |
| 0.2014097329349997 | 0.5949778785510161 | 0.1321970365760023 |
| 0.7014097329350055 | 0.5949778785509992 | 0.1321970365760023 |
| 0.2014097329349997 | 0.9283112118840239 | 0.1321970365760023 |
| 0.7014097329350055 | 0.9283112118840009 | 0.1321970365760023 |
| 0.4435183522890011 | 0.0949778074770009 | 0.1457107178800001 |
| 0.9435183522889976 | 0.0949778074770036 | 0.1457107178800001 |
| 0.4435183522890011 | 0.4283111408100096 | 0.1457107178800001 |
| 0.9435183522889976 | 0.4283111408100099 | 0.1457107178800001 |

| | | |
|---|---|---|
| 0.4435183522890011 | 0.7616444741430204 | 0.1457107178800001 |
| 0.9435183522889976 | 0.7616444741430212 | 0.1457107178800001 |
| 0.4392793396900067 | 0.2616444789110020 | 0.1979144111660034 |
| 0.9392793396900032 | 0.2616444789110020 | 0.1979144111660034 |
| 0.4392793396900067 | 0.5949778122439966 | 0.1979144111660034 |
| 0.9392793396900032 | 0.5949778122439966 | 0.1979144111660034 |
| 0.4392793396900067 | 0.9283111455780129 | 0.1979144111660034 |
| 0.9392793396900032 | 0.9283111455780051 | 0.1979144111660034 |
| 0.1813874112409991 | 0.0949778936079966 | 0.2114279908170005 |
| 0.6813874112409977 | 0.0949778936079997 | 0.2114279908170005 |
| 0.1813874112409991 | 0.4283112269410131 | 0.2114279908170005 |
| 0.6813874112409977 | 0.4283112269409983 | 0.2114279908170005 |
| 0.1813874112409991 | 0.7616445602750019 | 0.2114279908170005 |
| 0.6813874112409977 | 0.7616445602750019 | 0.2114279908170005 |
| 0.4343069956900045 | 0.0949774386079989 | 0.2707694632339972 |
| 0.9343069956900010 | 0.0949774386079989 | 0.2707694632339972 |
| 0.4343069956900045 | 0.4283107719410115 | 0.2707694632339972 |
| 0.9343069956900010 | 0.4283107719409998 | 0.2707694632339972 |
| 0.4343069956900045 | 0.7616441052750205 | 0.2707694632339972 |
| 0.9343069956900010 | 0.7616441052750189 | 0.2707694632339972 |
| 0.1912715103119501 | 0.0954271447487794 | 0.3296739984249494 |
| 0.6916796742039949 | 0.0953080621000533 | 0.3301461696924261 |
| 0.6912939238216923 | 0.4269006851127968 | 0.3302351413574559 |
| 0.1905530347069233 | 0.4288732771626473 | 0.3303569167322007 |
| 0.1904249373535915 | 0.7628997102195463 | 0.3304046653120576 |
| 0.6909131016721638 | 0.7650790015247912 | 0.3302896239033012 |
| 0.9341249556202755 | 0.2619322323784683 | 0.3456252735871644 |
| 0.4325128906337067 | 0.2626756769340949 | 0.3455823775094977 |
| 0.4362629849753926 | 0.5956786153943101 | 0.3445363117420641 |
| 0.9293648271669774 | 0.5956859434754037 | 0.3452159181945160 |
| 0.4321029883087437 | 0.9283984104382451 | 0.3459380309350358 |
| 0.9343909893000261 | 0.9293432953223620 | 0.3457487057011362 |
| 0.4277910457744480 | 0.0957271423450463 | 0.3958273030529970 |
| 0.9316041139255455 | 0.0955964970552833 | 0.3970604441606844 |
| 0.4312697121402703 | 0.4295440585334169 | 0.3979799221448573 |
| 0.9281149953101875 | 0.4286704376980658 | 0.3968207382307693 |
| 0.9285096222062664 | 0.7627162004235538 | 0.3967559248461605 |
| 0.4311057172964867 | 0.7614086093450916 | 0.3985173647432369 |
| 0.1711136183404821 | 0.2626791858076529 | 0.4132924799974296 |
| 0.6724258117106101 | 0.2600760368920386 | 0.4124020306751391 |
| 0.1709469056146306 | 0.5947886369363322 | 0.4102893046113407 |
| 0.6733486208058161 | 0.5950522016312927 | 0.4150065317611736 |
| 0.6728442211189454 | 0.9287103439126417 | 0.4126382739973684 |
| 0.1716300806760996 | 0.9268738283819151 | 0.4140154745520005 |

| | | |
|---|---|---|
| 0.4285326988422842 | 0.2537277403831892 | 0.4712024097137553 |
| 0.9232277691424021 | 0.2643395739993712 | 0.4720396952042776 |
| 0.4028138981414485 | 0.5943063609625711 | 0.4747676352100310 |
| 0.9389885502581585 | 0.5991695205983115 | 0.4714425386858876 |
| 0.9225218582900104 | 0.9321708612010021 | 0.4722108230536437 |
| 0.4309907511750724 | 0.9477472310572049 | 0.4710856263106420 |
| 0.9070381307236195 | 0.4310336314756548 | 0.5438236440228957 |
| 0.4083520757533811 | 0.0964610926568919 | 0.5467162532070783 |
| 0.8978164954298916 | 0.0961195099385656 | 0.5436546350604445 |
| 0.3980157188193421 | 0.4249218434504238 | 0.5388235380383367 |
| 0.3838575053232249 | 0.7678490387976202 | 0.5357887085805466 |
| 0.9193756129361219 | 0.7594355394214108 | 0.5436497566321786 |
| 0.6613553937234378 | 0.2655344710422577 | 0.5406989135854408 |
| 0.1579085669567752 | 0.2602731855755756 | 0.5404002537490693 |
| 0.6620803529023382 | 0.5982527703290381 | 0.5304821202476494 |
| 0.1513864230482370 | 0.5773003781089745 | 0.5512879307257275 |
| 0.1591366503440395 | 0.9357200767300914 | 0.5397976708988925 |
| 0.6596643084378446 | 0.9211135487742893 | 0.5407955647159216 |
| 0.4686436127425475 | 0.6022518518071786 | 0.5959549547214233 |
| 0.4212494542350064 | 0.2616454108449986 | 0.0151065289700014 |
| 0.9212494542350096 | 0.2616454108449986 | 0.0151065289700014 |
| 0.4212494542350064 | 0.5949787441780003 | 0.0151065289700014 |
| 0.9212494542350096 | 0.5949787441780003 | 0.0151065289700014 |
| 0.4212494542350064 | 0.9283120775110097 | 0.0151065289700014 |
| 0.9212494542350096 | 0.9283120775110224 | 0.0151065289700014 |
| 0.4757319089200007 | 0.2616445689349973 | 0.1306041949610020 |
| 0.9757319089200046 | 0.2616445689349974 | 0.1306041949610020 |
| 0.4757319089200007 | 0.5949779022690180 | 0.1306041949610020 |
| 0.9757319089200046 | 0.5949779022690009 | 0.1306041949610020 |
| 0.4757319089200007 | 0.9283112356020061 | 0.1306041949610020 |
| 0.9757319089200046 | 0.9283112356020203 | 0.1306041949610020 |
| 0.4070655843149993 | 0.0949779281480007 | 0.2130205561419984 |
| 0.9070655843150023 | 0.0949779281479977 | 0.2130205561419984 |
| 0.4070655843149993 | 0.4283112614820013 | 0.2130205561419984 |
| 0.9070655843150023 | 0.4283112614820013 | 0.2130205561419984 |
| 0.4070655843149993 | 0.7616445948150249 | 0.2130205561419984 |
| 0.9070655843150023 | 0.7616445948150198 | 0.2130205561419984 |
| 0.9635209255221079 | 0.0956259813098936 | 0.3289004484002077 |
| 0.4635437660751225 | 0.0956423954144601 | 0.3291911986480502 |
| 0.4631224043898766 | 0.4308556630680253 | 0.3293094849879287 |
| 0.9627122796207189 | 0.4286889389820003 | 0.3289295769722274 |
| 0.4626965962741896 | 0.7601407383541637 | 0.3293544806840422 |
| 0.9625068327713814 | 0.7625463542560368 | 0.3289353514890965 |
| 0.3995378781839602 | 0.2629454207619015 | 0.4127080543839047 |

| | | |
|---|---|---|
| 0.9000372068927456 | 0.2623728079036383 | 0.4130356729407468 |
| 0.3989139456214123 | 0.5953646681995639 | 0.4183240230845175 |
| 0.9022746888358348 | 0.5960315770127987 | 0.4124179395398365 |
| 0.3999909146061236 | 0.9289212342448132 | 0.4126177340981960 |
| 0.9004682075679845 | 0.9295697440568761 | 0.4130643296355976 |
| 0.4326124112200189 | 0.2626939963889478 | 0.5289391053178989 |
| 0.9288389152864429 | 0.2640984006716858 | 0.5297574158783770 |
| 0.4151566757290731 | 0.6026451167892403 | 0.5389384619564166 |
| 0.8989954745560773 | 0.5996041961476124 | 0.5279045364836283 |
| 0.4321453712214446 | 0.9371428804058317 | 0.5285717804631246 |
| 0.9310403449965978 | 0.9332246884936812 | 0.5298330141347413 |

NbO$_x$: $CH_3OH$ adsorption on O$_{vac}$

($E$ = -1163.5018 eV, $ZPE$ = 1.445002558 eV, $S*T$ = 0.389615451 eV)

AutoCreatByScript: H C O Nb

1.00000000000000

| | | |
|---|---|---|
| 7.9332688600000001 | 0.0000006300000000 | 0.0000000000000000 |
| 0.0000000000000000 | 11.4522889699999997 | 0.0000000000000000 |
| 0.0000000000000000 | 0.0000000000000000 | 32.7181709299999994 |

| H | C | O | Nb |
|---|---|---|---|
| 4 | 1 | 90 | 36 |

Selective dynamics

Direct

| | | |
|---|---|---|
| 0.6704747490214820 | 0.5096411778830169 | 0.5712547674208370 |
| 0.6654198685167917 | 0.7556343779740589 | 0.5929274953259149 |
| 0.7793494894395001 | 0.6444916154067206 | 0.6205778090667811 |
| 0.5515193917888288 | 0.6439489820585211 | 0.6203763705931693 |
| 0.6656155723225136 | 0.6646688272791907 | 0.6028911705546524 |
| 0.4534627765720096 | 0.0949785566820060 | 0.0000000000000000 |
| 0.9534627765720401 | 0.0949785566820012 | 0.0000000000000000 |
| 0.4534627765720096 | 0.4283118900150237 | 0.0000000000000000 |
| 0.9534627765720401 | 0.4283118900150030 | 0.0000000000000000 |
| 0.4534627765720096 | 0.7616452233490510 | 0.0000000000000000 |
| 0.9534627765720401 | 0.7616452233489994 | 0.0000000000000000 |
| 0.1955712230439985 | 0.2616453474379983 | 0.0135137049989993 |
| 0.6955712230439985 | 0.2616453474379983 | 0.0135137049989993 |
| 0.1955712230439985 | 0.5949786807720306 | 0.0135137049989993 |
| 0.6955712230439985 | 0.5949786807720017 | 0.0135137049989993 |
| 0.1955712230439985 | 0.9283120141050479 | 0.0135137049989993 |
| 0.6955712230439985 | 0.9283120141050035 | 0.0135137049989993 |
| 0.4484906587389972 | 0.2616449757260132 | 0.0728553503239981 |
| 0.9484906587390211 | 0.2616449757260000 | 0.0728553503239981 |
| 0.4484906587389972 | 0.5949783090590017 | 0.0728553503239981 |
| 0.9484906587390211 | 0.5949783090590307 | 0.0728553503239981 |
| 0.4484906587389972 | 0.9283116423920035 | 0.0728553503239981 |
| 0.9484906587390211 | 0.9283116423920035 | 0.0728553503239981 |
| 0.2014097329350001 | 0.2616445452179974 | 0.1321970365760023 |
| 0.7014097329350060 | 0.2616445452180119 | 0.1321970365760023 |
| 0.2014097329350001 | 0.5949778785510281 | 0.1321970365760023 |
| 0.7014097329350060 | 0.5949778785509992 | 0.1321970365760023 |
| 0.2014097329350001 | 0.9283112118840465 | 0.1321970365760023 |
| 0.7014097329350060 | 0.9283112118840009 | 0.1321970365760023 |
| 0.4435183522890012 | 0.0949778074770009 | 0.1457107178800001 |
| 0.9435183522889974 | 0.0949778074770081 | 0.1457107178800001 |
| 0.4435183522890012 | 0.4283111408100180 | 0.1457107178800001 |
| 0.9435183522889974 | 0.4283111408100090 | 0.1457107178800001 |

| | | |
|---|---|---|
| 0.4435183522890012 | 0.7616444741430454 | 0.1457107178800001 |
| 0.9435183522889974 | 0.7616444741430477 | 0.1457107178800001 |
| 0.4392793396900074 | 0.2616444789110020 | 0.1979144111660034 |
| 0.9392793396900032 | 0.2616444789110020 | 0.1979144111660034 |
| 0.4392793396900074 | 0.5949778122439966 | 0.1979144111660034 |
| 0.9392793396900032 | 0.5949778122439966 | 0.1979144111660034 |
| 0.4392793396900074 | 0.9283111455780004 | 0.1979144111660034 |
| 0.9392793396900032 | 0.9283111455780004 | 0.1979144111660034 |
| 0.1813874112409989 | 0.0949778936079966 | 0.2114279908170005 |
| 0.6813874112409977 | 0.0949778936080025 | 0.2114279908170005 |
| 0.1813874112409989 | 0.4283112269410266 | 0.2114279908170005 |
| 0.6813874112409977 | 0.4283112269409983 | 0.2114279908170005 |
| 0.1813874112409989 | 0.7616445602750019 | 0.2114279908170005 |
| 0.6813874112409977 | 0.7616445602750019 | 0.2114279908170005 |
| 0.4343069956900041 | 0.0949774386080001 | 0.2707694632339971 |
| 0.9343069956900010 | 0.0949774386079996 | 0.2707694632339971 |
| 0.4343069956900041 | 0.4283107719410379 | 0.2707694632339971 |
| 0.9343069956900010 | 0.4283107719409998 | 0.2707694632339971 |
| 0.4343069956900041 | 0.7616441052750449 | 0.2707694632339971 |
| 0.9343069956900010 | 0.7616441052750121 | 0.2707694632339971 |
| 0.1878523446077165 | 0.0964464001524932 | 0.3306715868720819 |
| 0.6879598303504793 | 0.0961411762981955 | 0.3305124654258595 |
| 0.6877407190451780 | 0.4317378457958396 | 0.3306830444435504 |
| 0.1879967515465128 | 0.4282602494676797 | 0.3303360084424437 |
| 0.1878718894428447 | 0.7646974403774681 | 0.3303185939977127 |
| 0.6876032788223235 | 0.7615165118574926 | 0.3307748439043772 |
| 0.9294631376903799 | 0.2625326102640718 | 0.3468795215165664 |
| 0.4314791354711328 | 0.2626294888228239 | 0.3468214446669456 |
| 0.4246940252114849 | 0.5962573227802032 | 0.3465207957056131 |
| 0.9351891105113322 | 0.5962936043170052 | 0.3463272233396509 |
| 0.4306271891197642 | 0.9297409333103979 | 0.3469337399301764 |
| 0.9297656526999026 | 0.9299693394594880 | 0.3469594733347089 |
| 0.4269846449997077 | 0.0963845763967067 | 0.3980160644834737 |
| 0.9252349896342590 | 0.0964599492948276 | 0.3981718677565808 |
| 0.4228544456956386 | 0.4289940876197156 | 0.3974104762100971 |
| 0.9274461779341246 | 0.4291834462800094 | 0.3974213987910376 |
| 0.9279465636731069 | 0.7633619904601802 | 0.3976628299187557 |
| 0.4222738017765061 | 0.7633718254108439 | 0.3976363641429237 |
| 0.1698919631454780 | 0.2612494488037749 | 0.4165111135995559 |
| 0.6698881088273373 | 0.2633956271070140 | 0.4168557297169735 |
| 0.1679642431845640 | 0.5960836925281439 | 0.4197407750607827 |
| 0.6670064924959315 | 0.5963260157848114 | 0.4115062795867832 |
| 0.6693897038519800 | 0.9281421029893286 | 0.4164789371274619 |
| 0.1694856416164483 | 0.9305872806924113 | 0.4170386296048350 |

| | | |
|---|---|---|
| 0.4211308123964114 | 0.2688446712073130 | 0.4745315080029218 |
| 0.9233677435808063 | 0.2702234899891313 | 0.4745465220408877 |
| 0.4462494320922250 | 0.5954300344421727 | 0.4726049269344270 |
| 0.8891405290184136 | 0.5956975096703940 | 0.4730959364754865 |
| 0.9206091133050799 | 0.9305705199266476 | 0.4744735241321525 |
| 0.4235296935447990 | 0.9309578402754615 | 0.4744422031874376 |
| 0.8966880054947227 | 0.4410654749891812 | 0.5487192403823665 |
| 0.4114025300961144 | 0.0995344873327527 | 0.5439056206560050 |
| 0.9227694145585028 | 0.1000455882288207 | 0.5436139699722073 |
| 0.4343742977877341 | 0.4407310308517223 | 0.5483892988289343 |
| 0.4235263945829021 | 0.7561667605405958 | 0.5455799495447726 |
| 0.9100243871570595 | 0.7564847196123106 | 0.5458679963086920 |
| 0.6667097062275774 | 0.2665909320609564 | 0.5413831147422251 |
| 0.1663937858924043 | 0.2796412687396003 | 0.5417402015717208 |
| 0.6666130240811121 | 0.5946966874005551 | 0.5653265038741024 |
| 0.1675604575340358 | 0.5911304273549610 | 0.5294641203435267 |
| 0.1670015812067254 | 0.9228451598062765 | 0.5415314963174572 |
| 0.6667986737222242 | 0.9291406024951177 | 0.5427415813701585 |
| 0.4212494542350059 | 0.2616454108449986 | 0.0151065289700014 |
| 0.9212494542350090 | 0.2616454108449986 | 0.0151065289700014 |
| 0.4212494542350059 | 0.5949787441780003 | 0.0151065289700014 |
| 0.9212494542350090 | 0.5949787441780003 | 0.0151065289700014 |
| 0.4212494542350059 | 0.9283120775110023 | 0.0151065289700014 |
| 0.9212494542350090 | 0.9283120775110477 | 0.0151065289700014 |
| 0.4757319089200007 | 0.2616445689349973 | 0.1306041949610020 |
| 0.9757319089200047 | 0.2616445689349974 | 0.1306041949610020 |
| 0.4757319089200007 | 0.5949779022690319 | 0.1306041949610020 |
| 0.9757319089200047 | 0.5949779022690009 | 0.1306041949610020 |
| 0.4757319089200007 | 0.9283112356020035 | 0.1306041949610020 |
| 0.9757319089200047 | 0.9283112356020393 | 0.1306041949610020 |
| 0.4070655843149993 | 0.0949779281480025 | 0.2130205561419984 |
| 0.9070655843150027 | 0.0949779281479977 | 0.2130205561419984 |
| 0.4070655843149993 | 0.4283112614820013 | 0.2130205561419984 |
| 0.9070655843150027 | 0.4283112614820013 | 0.2130205561419984 |
| 0.4070655843149993 | 0.7616445948150534 | 0.2130205561419984 |
| 0.9070655843150027 | 0.7616445948150538 | 0.2130205561419984 |
| 0.9575666471834866 | 0.0963252771334696 | 0.3291317773699245 |
| 0.4578084599042360 | 0.0962126616187169 | 0.3291090540904684 |
| 0.4577558806679204 | 0.4288032549022136 | 0.3290591415175230 |
| 0.9581797748369048 | 0.4284751002437429 | 0.3290137441452359 |
| 0.4575040415292295 | 0.7633259678620758 | 0.3290717811171807 |
| 0.9580339260848738 | 0.7637102739641051 | 0.3290063953287823 |
| 0.4005107746434239 | 0.2627901699757454 | 0.4139299120533652 |
| 0.9006309611079508 | 0.2631508415926573 | 0.4139621421033218 |

| | | |
|---|---|---|
| 0.3983736180686995 | 0.5960849124720313 | 0.4122349430923953 |
| 0.8973887643195853 | 0.5962384978321071 | 0.4117848456850597 |
| 0.3997370765268748 | 0.9300086788610650 | 0.4137108291093666 |
| 0.8997748643109895 | 0.9298549747594531 | 0.4136592740184594 |
| 0.4265572108204158 | 0.2659024108209990 | 0.5312326949647200 |
| 0.9265395760261956 | 0.2670038874729438 | 0.5312534159313451 |
| 0.4049478289358026 | 0.5976417753891037 | 0.5280720474409663 |
| 0.9306027395397163 | 0.5981873025959938 | 0.5284054755757828 |
| 0.4238909729110156 | 0.9324305920009611 | 0.5310139790266732 |
| 0.9244522626435092 | 0.9325842877902695 | 0.5310210000735636 |

$NbO_x$: Transition state for $CH_3OH$ dissociation on $O_{vac}$

($E$ = -1163.4727 eV, $ZPE$ = 1.323233956 eV, $S*T$ = 0.342454862 eV)

AutoCreatByScript: H C O Nb

1.000000000

| | | |
|---|---|---|
| 7.93326886 | 0.00000063 | 0.00000000 |
| 0.00000000 | 11.45228897 | 0.00000000 |
| 0.00000000 | 0.00000000 | 32.71817093 |

| H | C | O | Nb |
|---|---|---|---|
| 4 | 1 | 90 | 36 |

Selective Dynamics

Direct

| | | |
|---|---|---|
| 0.5996599641273221 | 0.5017076164656207 | 0.5643906243875106 |
| 0.6743729192105058 | 0.7381858384197806 | 0.6011053069585512 |
| 0.7664265748835344 | 0.6105238424796099 | 0.6245582756969843 |
| 0.5421238994791866 | 0.6230139090231095 | 0.6202133573852564 |
| 0.6630116655343005 | 0.6438054559762516 | 0.6054621907924606 |
| 0.4534627759987453 | 0.0949785564412324 | 0.0000000000000000 |
| 0.9534627759987454 | 0.0949785568778262 | 0.0000000000000000 |
| 0.4534627759987453 | 0.4283118900656284 | 0.0000000000000000 |
| 0.9534627759987454 | 0.4283118896290343 | 0.0000000000000000 |
| 0.4534627759987453 | 0.7616452236900243 | 0.0000000000000000 |
| 0.9534627759987454 | 0.7616452232534301 | 0.0000000000000000 |
| 0.1955712225792383 | 0.2616453474619344 | 0.0135137049973227 |
| 0.6955712225792385 | 0.2616453470253403 | 0.0135137049973227 |
| 0.1955712225792383 | 0.5949786810863302 | 0.0135137049973227 |
| 0.6955712225792385 | 0.5949786806497360 | 0.0135137049973227 |
| 0.1955712225792383 | 0.9283120138375383 | 0.0135137049973227 |
| 0.6955712225792385 | 0.9283120142741320 | 0.0135137049973227 |
| 0.4484906591203063 | 0.2616449755415910 | 0.0728553504748134 |
| 0.9484906591203064 | 0.2616449759781847 | 0.0728553504748134 |
| 0.4484906591203063 | 0.5949783091659868 | 0.0728553504748134 |
| 0.9484906591203064 | 0.5949783087293927 | 0.0728553504748134 |
| 0.4484906591203063 | 0.9283116427903827 | 0.0728553504748134 |
| 0.9484906591203064 | 0.9283116423537886 | 0.0728553504748134 |
| 0.2014097326332138 | 0.2616445455542733 | 0.1321970366024981 |
| 0.7014097326332139 | 0.2616445451176792 | 0.1321970366024981 |
| 0.2014097326332138 | 0.5949778783054813 | 0.1321970366024981 |
| 0.7014097326332139 | 0.5949778787420751 | 0.1321970366024981 |
| 0.2014097326332138 | 0.9283112119298773 | 0.1321970366024981 |
| 0.7014097326332139 | 0.9283112114932831 | 0.1321970366024981 |
| 0.4435183519041861 | 0.0949778077930770 | 0.1457107177598574 |
| 0.9435183519041862 | 0.0949778073564829 | 0.1457107177598574 |
| 0.4435183519041861 | 0.4283111405442850 | 0.1457107177598574 |
| 0.9435183519041862 | 0.4283111409808788 | 0.1457107177598574 |

| | | |
|---|---|---|
| 0.4435183519041861 | 0.7616444741686811 | 0.1457107177598574 |
| 0.9435183519041862 | 0.7616444737320867 | 0.1457107177598574 |
| 0.4392793401432762 | 0.2616444792044060 | 0.1979144110425368 |
| 0.9392793401432764 | 0.2616444787678119 | 0.1979144110425368 |
| 0.4392793401432762 | 0.5949778119556141 | 0.1979144110425368 |
| 0.9392793401432764 | 0.5949778123922078 | 0.1979144110425368 |
| 0.4392793401432762 | 0.9283111455800099 | 0.1979144110425368 |
| 0.9392793401432764 | 0.9283111451434157 | 0.1979144110425368 |
| 0.1813874110904645 | 0.0949778938145262 | 0.2114279907272310 |
| 0.6813874110904646 | 0.0949778933779321 | 0.2114279907272310 |
| 0.1813874110904645 | 0.4283112265657342 | 0.2114279907272310 |
| 0.6813874110904646 | 0.4283112270023280 | 0.2114279907272310 |
| 0.1813874110904645 | 0.7616445601901303 | 0.2114279907272310 |
| 0.6813874110904646 | 0.7616445606267240 | 0.2114279907272310 |
| 0.4343069951117225 | 0.0949774389413255 | 0.2707694632121662 |
| 0.9343069951117228 | 0.0949774385047314 | 0.2707694632121662 |
| 0.4343069951117225 | 0.4283107716925336 | 0.2707694632121662 |
| 0.9343069951117228 | 0.4283107721291273 | 0.2707694632121662 |
| 0.4343069951117225 | 0.7616441053169295 | 0.2707694632121662 |
| 0.9343069951117228 | 0.7616441048803353 | 0.2707694632121662 |
| 0.1861015120064897 | 0.0969449946350809 | 0.3309363012121167 |
| 0.6863396748663831 | 0.0962466228797929 | 0.3305286292787272 |
| 0.6860326627074633 | 0.4331950050156147 | 0.3307918472323966 |
| 0.1862113179408873 | 0.4286317884176536 | 0.3304570516222313 |
| 0.1854689896391587 | 0.7649825014112035 | 0.3305374213350012 |
| 0.6853054000743902 | 0.7611691008751758 | 0.3311744548062363 |
| 0.9279633578433900 | 0.2628058891342383 | 0.3471367618409835 |
| 0.4313704653645132 | 0.2634641953360180 | 0.3470633170263225 |
| 0.4219795369446235 | 0.5969407969062878 | 0.3470287151531792 |
| 0.9332886380961507 | 0.5963722719466231 | 0.3474341565217808 |
| 0.4285084105923017 | 0.9302637130400402 | 0.3470391332172186 |
| 0.9272822476862334 | 0.9294580920631605 | 0.3472842352437701 |
| 0.4269951541765851 | 0.0970937315593295 | 0.3982855938945973 |
| 0.9233069065555408 | 0.0960033624017649 | 0.3983900248546076 |
| 0.4219558443151012 | 0.4301451742155800 | 0.3978071967362266 |
| 0.9266722645777068 | 0.4295505055000781 | 0.3981382326618953 |
| 0.9260599583410567 | 0.7627499061073919 | 0.3985608929025788 |
| 0.4193316902157783 | 0.7639600274442806 | 0.3978728993699281 |
| 0.1713402323793171 | 0.2611928759299943 | 0.4166152423117743 |
| 0.6711129389354896 | 0.2637485445146643 | 0.4173408843426444 |
| 0.1659541890276993 | 0.5955868563320804 | 0.4213378238500449 |
| 0.6653620119462335 | 0.5961709933016062 | 0.4131434024511956 |
| 0.6665327021325737 | 0.9277516327012829 | 0.4169531034967301 |
| 0.1666545774927891 | 0.9312270789659977 | 0.4174141191822424 |

| | | |
|---|---|---|
| 0.4255386032637246 | 0.2742643134607070 | 0.4749814072201254 |
| 0.9294308033826049 | 0.2673625728864741 | 0.4748533160132861 |
| 0.4409829178536124 | 0.6041807214528188 | 0.4734514463886627 |
| 0.8849319156945857 | 0.5958534010422274 | 0.4754605256902116 |
| 0.9164895767820986 | 0.9304838194815094 | 0.4751879954800395 |
| 0.4192766196001582 | 0.9333817199214216 | 0.4746234785930926 |
| 0.9143129393449048 | 0.4373104623103868 | 0.5484609970524412 |
| 0.4195367141508930 | 0.1039400174768615 | 0.5437182248989492 |
| 0.9336073642158148 | 0.1005877134994574 | 0.5452148553219872 |
| 0.4573985067234947 | 0.4494039807518879 | 0.5512457761953504 |
| 0.4305868879880621 | 0.7615128706213792 | 0.5466786813440001 |
| 0.9122777228049224 | 0.7589037753092104 | 0.5474981217111698 |
| 0.6768706361478339 | 0.2688282998827871 | 0.5410185400605462 |
| 0.1804720759205431 | 0.2832784096525108 | 0.5417923883314096 |
| 0.6692383863062472 | 0.5884526723027503 | 0.5654814537030112 |
| 0.1658973650364852 | 0.5946396256088059 | 0.5325495137634211 |
| 0.1745673825555939 | 0.9253513605693225 | 0.5420311370076946 |
| 0.6725491161533635 | 0.9339672596729849 | 0.5428205674454553 |
| 0.4212494545407353 | 0.2616454110145320 | 0.0151065290617088 |
| 0.9212494545407355 | 0.2616454105779379 | 0.0151065290617088 |
| 0.4212494545407353 | 0.5949787437657400 | 0.0151065290617088 |
| 0.9212494545407355 | 0.5949787442023338 | 0.0151065290617088 |
| 0.4212494545407353 | 0.9283120773901359 | 0.0151065290617088 |
| 0.9212494545407355 | 0.9283120778267295 | 0.0151065290617088 |
| 0.4757319090783974 | 0.2616445688838477 | 0.1306041948109597 |
| 0.9757319090783976 | 0.2616445693204415 | 0.1306041948109597 |
| 0.4757319090783974 | 0.5949779025082436 | 0.1306041948109597 |
| 0.9757319090783976 | 0.5949779020716495 | 0.1306041948109597 |
| 0.4757319090783974 | 0.9283112352594517 | 0.1306041948109597 |
| 0.9757319090783976 | 0.9283112356960453 | 0.1306041948109597 |
| 0.4070655838077825 | 0.0949779285519269 | 0.2130205562197055 |
| 0.9070655838077826 | 0.0949779281153327 | 0.2130205562197055 |
| 0.4070655838077825 | 0.4283112613031349 | 0.2130205562197055 |
| 0.9070655838077826 | 0.4283112617397287 | 0.2130205562197055 |
| 0.4070655838077825 | 0.7616445949275308 | 0.2130205562197055 |
| 0.9070655838077826 | 0.7616445944909367 | 0.2130205562197055 |
| 0.9555675338601851 | 0.0960842728361970 | 0.3293360329663148 |
| 0.4560314951937732 | 0.0969158711946260 | 0.3291926536187944 |
| 0.4557185863482762 | 0.4296212535141163 | 0.3291766707571266 |
| 0.9560329599115595 | 0.4288276693475042 | 0.3292713426141392 |
| 0.4549536532409921 | 0.7637930745803732 | 0.3291772627828862 |
| 0.9553628553060285 | 0.7632422567242813 | 0.3292530735611020 |
| 0.4022016720103951 | 0.2638144858663084 | 0.4143093533254550 |
| 0.9022060283483198 | 0.2627651152964399 | 0.4144790110368189 |

| | | | | | |
|---|---|---|---|---|---|
| 0.3960106931255575 | 0.5971105678896662 | 0.4127843744350778 | T | T | T |
| 0.8959511136497651 | 0.5959307317016462 | 0.4133639731553295 | T | T | T |
| 0.3967057723038018 | 0.9308875202155644 | 0.4138181241539226 | T | T | T |
| 0.8968436272056463 | 0.9294359619174463 | 0.4143506490324458 | T | T | T |
| 0.4366670739556910 | 0.2691880184804436 | 0.5315829481180597 | T | T | T |
| 0.9379982755305235 | 0.2664829002355397 | 0.5316546431405296 | T | T | T |
| 0.3992748671321346 | 0.6070524832780945 | 0.5288425950527302 | T | T | T |
| 0.9220234646125434 | 0.5974214078118230 | 0.5309662110136790 | T | T | T |
| 0.4113650334548224 | 0.9369086729253239 | 0.5311566177455630 | T | T | T |
| 0.9108939149202114 | 0.9329171997077915 | 0.5319257527333909 | T | T | T |

$NbO_x$: : Final state for $CH_3OH$ dissociation on $O_{vac}$
($E$ = -1164.0909 eV, $ZPE$ = 1.35506866 eV, $S*T$ = 0.235162441 eV)
AutoCreatByScript: H C O Nb
1.00000000000000
7.9332688600000001 0.0000006300000000 0.0000000000000000
0.0000000000000000 11.4522889699999997 0.0000000000000000
0.0000000000000000 0.0000000000000000 32.7181709299999994
H C O Nb
4 1 90 36
Selective dynamics
Direct
0.5707676889698230 0.4262492293923589 0.5584497411264676
0.6648643882850948 0.7617069665585002 0.5890753154870447
0.7748336758293862 0.6403902278962105 0.6116121744232494
0.5510321817491916 0.6408597029427269 0.6109190524384979
0.6641222911118119 0.6667037718208170 0.5934836631943503
0.4534627765720083 0.0949785566820056 0.0000000000000000
0.9534627765720117 0.0949785566820012 0.0000000000000000
0.4534627765720083 0.4283118900150149 0.0000000000000000
0.9534627765720117 0.4283118900150030 0.0000000000000000
0.4534627765720083 0.7616452233490336 0.0000000000000000
0.9534627765720117 0.7616452233489994 0.0000000000000000
0.1955712230439985 0.2616453474379991 0.0135137049989993
0.6955712230439985 0.2616453474379991 0.0135137049989993
0.1955712230439985 0.5949786807720229 0.0135137049989993
0.6955712230439985 0.5949786807720017 0.0135137049989993
0.1955712230439985 0.9283120141050401 0.0135137049989993
0.6955712230439985 0.9283120141050035 0.0135137049989993
0.4484906587389972 0.2616449757260097 0.0728553503239981
0.9484906587390074 0.2616449757260000 0.0728553503239981
0.4484906587389972 0.5949783090590017 0.0728553503239981
0.9484906587390074 0.5949783090590229 0.0728553503239981
0.4484906587389972 0.9283116423920035 0.0728553503239981
0.9484906587390074 0.9283116423920035 0.0728553503239981
0.2014097329349997 0.2616445452179974 0.1321970365760023
0.7014097329350059 0.2616445452180080 0.1321970365760023
0.2014097329349997 0.5949778785510204 0.1321970365760023
0.7014097329350059 0.5949778785509992 0.1321970365760023
0.2014097329349997 0.9283112118840375 0.1321970365760023
0.7014097329350059 0.9283112118840009 0.1321970365760023
0.4435183522890053 0.0949778074770009 0.1457107178800001
0.9435183522889974 0.0949778074770053 0.1457107178800001
0.4435183522890053 0.4283111408100069 0.1457107178800001
0.9435183522889974 0.4283111408100160 0.1457107178800001

| | | |
|---|---|---|
| 0.4435183522890053 | 0.7616444741430343 | 0.1457107178800001 |
| 0.9435183522889974 | 0.7616444741430317 | 0.1457107178800001 |
| 0.4392793396900066 | 0.2616444789110020 | 0.1979144111660034 |
| 0.9392793396900032 | 0.2616444789110020 | 0.1979144111660034 |
| 0.4392793396900066 | 0.5949778122439966 | 0.1979144111660034 |
| 0.9392793396900032 | 0.5949778122439966 | 0.1979144111660034 |
| 0.4392793396900066 | 0.9283111455780004 | 0.1979144111660034 |
| 0.9392793396900032 | 0.9283111455780004 | 0.1979144111660034 |
| 0.1813874112409990 | 0.0949778936079966 | 0.2114279908170005 |
| 0.6813874112409977 | 0.0949778936080010 | 0.2114279908170005 |
| 0.1813874112409990 | 0.4283112269410163 | 0.2114279908170005 |
| 0.6813874112409977 | 0.4283112269409983 | 0.2114279908170005 |
| 0.1813874112409990 | 0.7616445602750019 | 0.2114279908170005 |
| 0.6813874112409977 | 0.7616445602750019 | 0.2114279908170005 |
| 0.4343069956900049 | 0.0949774386079985 | 0.2707694632339972 |
| 0.9343069956900010 | 0.0949774386079985 | 0.2707694632339972 |
| 0.4343069956900049 | 0.4283107719410176 | 0.2707694632339972 |
| 0.9343069956900010 | 0.4283107719409998 | 0.2707694632339972 |
| 0.4343069956900049 | 0.7616441052750272 | 0.2707694632339972 |
| 0.9343069956900010 | 0.7616441052750070 | 0.2707694632339972 |
| 0.1870106637857920 | 0.0956331153259324 | 0.3306623247577736 |
| 0.6872247749906727 | 0.0952046009214761 | 0.3304115532363789 |
| 0.6867258054410040 | 0.4322606335043969 | 0.3307397833988036 |
| 0.1870673758107446 | 0.4288173519390228 | 0.3304404029279737 |
| 0.1872416059400475 | 0.7631205557491739 | 0.3305784768092490 |
| 0.6868878394938944 | 0.7603555302589183 | 0.3309137889732903 |
| 0.9276438112416531 | 0.2624397094357645 | 0.3461604629164108 |
| 0.4298130372026233 | 0.2627632606333152 | 0.3459239621571362 |
| 0.4245333820810452 | 0.5958578782432372 | 0.3472283718501593 |
| 0.9331755180652065 | 0.5958727716309361 | 0.3473060593518429 |
| 0.4297480950639195 | 0.9286489284485495 | 0.3464156857380292 |
| 0.9290185404728957 | 0.9287201076892369 | 0.3467833494658192 |
| 0.4245654886259300 | 0.0960529493200240 | 0.3969249959832616 |
| 0.9224689489413074 | 0.0958137877553795 | 0.3977818824377166 |
| 0.4213200463205403 | 0.4288238484052612 | 0.3976994547259335 |
| 0.9256146488365163 | 0.4291869826738094 | 0.3976578258811470 |
| 0.9268508887968635 | 0.7619857853692128 | 0.3981665773980778 |
| 0.4225639639125020 | 0.7624861003037651 | 0.3979835510349660 |
| 0.1655407772320576 | 0.2613223879982706 | 0.4148222717413111 |
| 0.6654810003129279 | 0.2634147755115617 | 0.4149963756132743 |
| 0.1687738108400082 | 0.5957607734742849 | 0.4208333583434323 |
| 0.6679062023616352 | 0.5957385082155134 | 0.4147686163455542 |
| 0.6676242348681132 | 0.9272281793655394 | 0.4156724380116816 |
| 0.1679567289302743 | 0.9294180748891440 | 0.4162608180723620 |

| | | |
|---|---|---|
| 0.4143001092062635 | 0.2583651188651236 | 0.4735679192747937 |
| 0.9146150348136087 | 0.2662011870543958 | 0.4739821836241951 |
| 0.4409307798231564 | 0.5939816315123594 | 0.4740293888169713 |
| 0.9011412225444688 | 0.5917531010081536 | 0.4749802326895812 |
| 0.9198006301098886 | 0.9319266280206853 | 0.4738745831409450 |
| 0.4241614389683416 | 0.9402921041645571 | 0.4730977377112073 |
| 0.9082490045187610 | 0.4358066689256554 | 0.5491089099750972 |
| 0.4184037980275161 | 0.1002076202026663 | 0.5477247002691472 |
| 0.9215339806469338 | 0.1019406502961527 | 0.5452568708794165 |
| 0.4484884483435653 | 0.4329602417101605 | 0.5533816169211786 |
| 0.4121504157780896 | 0.7643081330734025 | 0.5423180975693959 |
| 0.9302541429673541 | 0.7603871133005422 | 0.5449647707748658 |
| 0.6654005972661965 | 0.2711315139305124 | 0.5396727031220442 |
| 0.1713007248807353 | 0.2795662099448942 | 0.5396039477642001 |
| 0.6656852450024423 | 0.6090067503891092 | 0.5542322623630532 |
| 0.1760303305608636 | 0.5838322025432727 | 0.5366897316402638 |
| 0.1716640688941274 | 0.9332040967609929 | 0.5394743864267831 |
| 0.6696382408645625 | 0.9288859032197532 | 0.5408567348869471 |
| 0.4212494542350062 | 0.2616454108449986 | 0.0151065289700014 |
| 0.9212494542350097 | 0.2616454108449986 | 0.0151065289700014 |
| 0.4212494542350062 | 0.5949787441780003 | 0.0151065289700014 |
| 0.9212494542350097 | 0.5949787441780003 | 0.0151065289700014 |
| 0.4212494542350062 | 0.9283120775110023 | 0.0151065289700014 |
| 0.9212494542350097 | 0.9283120775110356 | 0.0151065289700014 |
| 0.4757319089200071 | 0.2616445689349973 | 0.1306041949610020 |
| 0.9757319089200166 | 0.2616445689349974 | 0.1306041949610020 |
| 0.4757319089200071 | 0.5949779022690229 | 0.1306041949610020 |
| 0.9757319089200166 | 0.5949779022690009 | 0.1306041949610020 |
| 0.4757319089200071 | 0.9283112356020046 | 0.1306041949610020 |
| 0.9757319089200166 | 0.9283112356020061 | 0.1306041949610020 |
| 0.4070655843149993 | 0.0949779281480010 | 0.2130205561419984 |
| 0.9070655843150012 | 0.0949779281479977 | 0.2130205561419984 |
| 0.4070655843149993 | 0.4283112614820013 | 0.2130205561419984 |
| 0.9070655843150012 | 0.4283112614820013 | 0.2130205561419984 |
| 0.4070655843149993 | 0.7616445948150374 | 0.2130205561419984 |
| 0.9070655843150012 | 0.7616445948150370 | 0.2130205561419984 |
| 0.9571103259744370 | 0.0959765060454654 | 0.3291614686335091 |
| 0.4575464499735860 | 0.0961939155216943 | 0.3289460714600520 |
| 0.4569211248652958 | 0.4284600672671196 | 0.3290029606383038 |
| 0.9572457162911848 | 0.4282115601163178 | 0.3291382983440340 |
| 0.4568850973345159 | 0.7624990601041043 | 0.3290002786142475 |
| 0.9572610551780194 | 0.7626535935406733 | 0.3291378619872052 |
| 0.3971829476059979 | 0.2618988846451641 | 0.4133866331600952 |
| 0.8973386924051765 | 0.2625067631489987 | 0.4139736298281214 |

| | | |
|---|---|---|
| 0.3977055870570871 | 0.5955155112989824 | 0.4125332131708405 |
| 0.8980359636199056 | 0.5953598379882038 | 0.4131012804254771 |
| 0.3976180849522414 | 0.9300271087335609 | 0.4126838858116144 |
| 0.8976362069271119 | 0.9292648332710836 | 0.4135207366282006 |
| 0.4039742587242403 | 0.2573210883318198 | 0.5304841723507654 |
| 0.9050896588076482 | 0.2655232020944366 | 0.5310753731587631 |
| 0.4117840903214741 | 0.6050393964893229 | 0.5293853732388648 |
| 0.9300335466539306 | 0.5957687051695147 | 0.5307251861081794 |
| 0.4236894717838843 | 0.9355256816513624 | 0.5295095230594171 |
| 0.9251737443712078 | 0.9322442343364961 | 0.5306101711295279 |

Ru: Initial state for $CH_3O$ and CO coupling

($E$ = -1129.4938 eV, $ZPE$ = 1.279792158 eV, $S*T$ = 0.660446654 eV)

AutoCreatByScript: H C O Ru

1.00000000000000

| | | |
|---|---|---|
| 15.1228015700000000 | 0.0000000000000000 | 0.0000000000000000 |
| -3.6418149199999998 | 13.0568463000000001 | 0.0000000000000000 |
| 0.0000000000000000 | 0.0000000000000000 | 21.5088567099999999 |

| | | | |
|---|---|---|---|
| H | C | O | Ru |
| 3 | 2 | 2 | 120 |

Selective dynamics

Direct

| | | |
|---|---|---|
| 0.7215403344373915 | 0.6564317110359889 | 0.3991758079813897 |
| 0.8452227829354348 | 0.6951454593967140 | 0.4012303287209217 |
| 0.7829167443339710 | 0.6074485070576909 | 0.4583515823915915 |
| 0.7742869869881482 | 0.0887647909884902 | 0.3636555369869706 |
| 0.7832739293021957 | 0.6299489671133875 | 0.4090979379918673 |
| 0.7855266642805724 | 0.5442542926261271 | 0.3709412144644978 |
| 0.7859101304036599 | 0.0725175344585698 | 0.4169463820872999 |
| 0.8073722568249989 | 0.1380862835040006 | 0.0000000000000000 |
| 0.1407055901600032 | 0.1380862835040006 | 0.0000000000000000 |
| 0.4740389234929985 | 0.1380862835040006 | 0.0000000000000000 |
| 0.1407055901590016 | 0.3380862835039939 | 0.0000000000000000 |
| 0.4740389234929993 | 0.3380862835039939 | 0.0000000000000000 |
| 0.8073722568260019 | 0.3380862835039939 | 0.0000000000000000 |
| 0.1407055901600022 | 0.5380862835049970 | 0.0000000000000000 |
| 0.4740389234929971 | 0.5380862835049970 | 0.0000000000000000 |
| 0.8073722568280033 | 0.5380862835049970 | 0.0000000000000000 |
| 0.1407055901600020 | 0.7380862835039910 | 0.0000000000000000 |
| 0.4740389234929972 | 0.7380862835039910 | 0.0000000000000000 |
| 0.8073722568259989 | 0.7380862835039910 | 0.0000000000000000 |
| 0.1407055901600021 | 0.9380862835039873 | 0.0000000000000000 |
| 0.4740389234929976 | 0.9380862835039873 | 0.0000000000000000 |
| 0.8073722568259986 | 0.9380862835039873 | 0.0000000000000000 |
| 0.3201838529240036 | 0.1920708306439960 | 0.0159535054489979 |
| 0.6535171862569991 | 0.1920708306439960 | 0.0159535054489979 |
| 0.9868505195880036 | 0.1920708306439960 | 0.0159535054489979 |
| 0.3201838529240035 | 0.3920708306439969 | 0.0159535054489979 |
| 0.6535171862569992 | 0.3920708306439969 | 0.0159535054489979 |
| 0.9868505195889982 | 0.3920708306439969 | 0.0159535054489979 |
| 0.3201838529240030 | 0.5920708306439909 | 0.0159535054489979 |
| 0.6535171862569982 | 0.5920708306439909 | 0.0159535054489979 |
| 0.9868505195900006 | 0.5920708306439909 | 0.0159535054489979 |
| 0.3201838529240035 | 0.7920708306449944 | 0.0159535054489979 |
| 0.6535171862569982 | 0.7920708306439925 | 0.0159535054489979 |

| | | |
|---|---|---|
| 0.9868505195910036 | 0.7920708306419870 | 0.0159535054489979 |
| 0.3201838529240036 | 0.9920708306439923 | 0.0159535054489979 |
| 0.6535171862569982 | 0.9920708306439923 | 0.0159535054489979 |
| 0.9868505195889979 | 0.9920708306439923 | 0.0159535054489979 |
| 0.2186901232590019 | 0.0615218340180015 | 0.0955531191689971 |
| 0.5520234565920038 | 0.0615218340180015 | 0.0955531191689971 |
| 0.8853567899250010 | 0.0615218340180015 | 0.0955531191689971 |
| 0.2186901232590018 | 0.2615218340179952 | 0.0955531191689971 |
| 0.5520234565920035 | 0.2615218340179952 | 0.0955531191689971 |
| 0.8853567899249987 | 0.2615218340179952 | 0.0955531191689971 |
| 0.2186901232590018 | 0.4615218340179955 | 0.0955531191689971 |
| 0.5520234565920035 | 0.4615218340179955 | 0.0955531191689971 |
| 0.8853567899249988 | 0.4615218340179955 | 0.0955531191689971 |
| 0.2186901232590003 | 0.6615218340179920 | 0.0955531191689971 |
| 0.5520234565920035 | 0.6615218340179920 | 0.0955531191689971 |
| 0.8853567899249971 | 0.6615218340179920 | 0.0955531191689971 |
| 0.2186901232599952 | 0.8615218340179887 | 0.0955531191689971 |
| 0.5520234565920035 | 0.8615218340179887 | 0.0955531191689971 |
| 0.8853567899249980 | 0.8615218340179887 | 0.0955531191689971 |
| 0.0648350526889985 | 0.1155063811579980 | 0.1115066246180014 |
| 0.3981683860230025 | 0.1155063811579980 | 0.1115066246180014 |
| 0.7315017193540015 | 0.1155063811579980 | 0.1115066246180014 |
| 0.0648350526889982 | 0.3155063811579973 | 0.1115066246180014 |
| 0.3981683860230022 | 0.3155063811579973 | 0.1115066246180014 |
| 0.7315017193559971 | 0.3155063811579973 | 0.1115066246180014 |
| 0.0648350526889978 | 0.5155063811589943 | 0.1115066246180014 |
| 0.3981683860230018 | 0.5155063811589943 | 0.1115066246180014 |
| 0.7315017193559975 | 0.5155063811589943 | 0.1115066246180014 |
| 0.0648350526889983 | 0.7155063811579934 | 0.1115066246180014 |
| 0.3981683860230020 | 0.7155063811579934 | 0.1115066246180014 |
| 0.7315017193559973 | 0.7155063811579934 | 0.1115066246180014 |
| 0.0648350526889974 | 0.9155063811579867 | 0.1115066246180014 |
| 0.3981683860230021 | 0.9155063811579867 | 0.1115066246180014 |
| 0.7315017193559967 | 0.9155063811579867 | 0.1115066246180014 |
| 0.9637798961069813 | 0.5854621906901840 | 0.1912891592045443 |
| 0.9630713576864377 | 0.3854308175112247 | 0.1927005816469426 |
| 0.9632251835354658 | 0.7844504419652680 | 0.1926269652157457 |
| 0.9644916987664396 | 0.1850693101837787 | 0.1918154678718250 |
| 0.6295591901239790 | 0.9852772952929431 | 0.1928271018101061 |
| 0.9639400136976222 | 0.9859846742206354 | 0.1926696343645328 |
| 0.2962927108209871 | 0.7846904549180361 | 0.1928954287454559 |
| 0.2968438536463731 | 0.9850684983300507 | 0.1929213947149394 |
| 0.2969508280410585 | 0.1852887857880574 | 0.1931048764120430 |
| 0.2967705479395595 | 0.3850447030413112 | 0.1929745125489012 |

| | | |
|---|---|---|
| 0.6304644603172632 | 0.5845350473349235 | 0.1931628480878853 |
| 0.2964627235908192 | 0.5852567614371265 | 0.1930738456983380 |
| 0.6308626239251187 | 0.7854493342376574 | 0.1933245831327886 |
| 0.6296982938145831 | 0.1854647497507263 | 0.1918391413980598 |
| 0.6309652315000405 | 0.3860434258603624 | 0.1929769166569256 |
| 0.4767470741260992 | 0.0389174844496779 | 0.2079847441560808 |
| 0.4768866142502680 | 0.4385027818337778 | 0.2080905689698682 |
| 0.1437002563218255 | 0.8388872837948178 | 0.2080387928961453 |
| 0.8096770369640922 | 0.2370217478102162 | 0.2088017847461524 |
| 0.4764200776759045 | 0.8387835957711925 | 0.2082521254241861 |
| 0.1436879092240546 | 0.4389325980807163 | 0.2082080809244968 |
| 0.8099230597330533 | 0.0390531206267683 | 0.2081924378563266 |
| 0.1438366568114903 | 0.0387097258899113 | 0.2082589648029388 |
| 0.8102089156511623 | 0.6385007424664886 | 0.2084326911581074 |
| 0.4765715487762007 | 0.6386487818198424 | 0.2082308312177099 |
| 0.8103877048995992 | 0.8393279856260654 | 0.2081978425991635 |
| 0.1434376788338836 | 0.2385518384369962 | 0.2082686565765438 |
| 0.1427682304798185 | 0.6381812714460362 | 0.2083502231325900 |
| 0.4770443631299207 | 0.2391596025087486 | 0.2085784751744860 |
| 0.8100029444454476 | 0.4393619696598717 | 0.2088013726560235 |
| 0.7039583213511200 | 0.7087944313206561 | 0.2847458403642354 |
| 0.3721714008509304 | 0.1075211191910149 | 0.2858629739751993 |
| 0.3716649551571449 | 0.5076491714276889 | 0.2856824471621059 |
| 0.3714515566542432 | 0.9072742723777025 | 0.2856522140658733 |
| 0.3720048672942288 | 0.3075132919974287 | 0.2860406929076541 |
| 0.0394926719965032 | 0.3081026301899246 | 0.2856209326814979 |
| 0.0365733422488626 | 0.5074205654762101 | 0.2854984162745965 |
| 0.0378957070985532 | 0.7071092969063572 | 0.2854307533248929 |
| 0.0391060132610361 | 0.9079610669619220 | 0.2853327425397758 |
| 0.7059350790372005 | 0.1071568784259941 | 0.2886936870966016 |
| 0.3715346822474128 | 0.7075445432121898 | 0.2858522429943048 |
| 0.7039733645509461 | 0.9062840433277819 | 0.2858865787319188 |
| 0.0393013668917775 | 0.1079362529876400 | 0.2858274381384770 |
| 0.7050016311907872 | 0.3072444404936501 | 0.2853458050303541 |
| 0.7058654852024768 | 0.5084576979430681 | 0.2894402433034594 |
| 0.8877666569157032 | 0.3611271669004314 | 0.2989179272764468 |
| 0.8880416531657448 | 0.7646319098948539 | 0.2986172474367431 |
| 0.8895258339868463 | 0.1632351127595838 | 0.3012669418444066 |
| 0.5520750969741655 | 0.7617918335352702 | 0.2991867354463877 |
| 0.8879927095458519 | 0.9621093564591702 | 0.2988299369486300 |
| 0.5516721663707147 | 0.5618883074226948 | 0.2991225122826647 |
| 0.5508642669790712 | 0.9604076354228788 | 0.2995516352332907 |
| 0.2204840781323700 | 0.1621803026598542 | 0.2994609001146462 |
| 0.2193917767282562 | 0.5618522918392208 | 0.2992477861166408 |

| | | |
|---|---|---|
| 0.2195634171072448 | 0.7619930679388337 | 0.2993276808255297 |
| 0.2200281699468064 | 0.9622001042941752 | 0.2992173549748584 |
| 0.2202312394658587 | 0.3622075557221783 | 0.2992368510264763 |
| 0.5528460532222405 | 0.1621028861066191 | 0.2989924357079397 |
| 0.5533056864628946 | 0.3623189159196010 | 0.2988977165316080 |
| 0.8886164962284457 | 0.5618949290832728 | 0.3040103462619126 |

Ru: Transition state for $CH_3O$ and CO coupling

($E$ = -1128.3366 eV, $ZPE$ = 1.23736119 eV, $S*T$ = 0.525245129 eV)

```
AutoCreatByScript: H C O Ru
   1.00000000000000
    15.1228015700000000     0.0000000000000000     0.0000000000000000
    -3.6418149199999998    13.0568463000000001     0.0000000000000000
     0.0000000000000000     0.0000000000000000    21.5088567099999999
   H    C    O    Ru
     3     2     2   120
Selective dynamics
Direct
  0.6260784738043597  0.5321345695153813  0.4200700146629057
  0.7412045548547221  0.6101751629305385  0.4322680695954081
  0.6993533527606433  0.4911967857840776  0.4745903757130893
  0.6832282034194477  0.3549230907318531  0.3766758480538424
  0.6974245886685532  0.5313304095089307  0.4305044389638595
  0.7330049399183796  0.4798547013245758  0.3814975309550824
  0.6459584215689150  0.3194440330116243  0.4240540843769676
  0.1407055901600032  0.1380862835039403  0.0000000000000000
  0.4740389234930254  0.1380862835039403  0.0000000000000000
  0.8073722568251527  0.1380862835039403  0.0000000000000000
  1.1407055901590524  0.3380862835038232  0.0000000000000000
  0.4740389234930072  0.3380862835038232  0.0000000000000000
  0.8073722568260429  0.3380862835038232  0.0000000000000000
  1.1407055901600029  0.5380862835047558  0.0000000000000000
  0.4740389234929944  0.5380862835047558  0.0000000000000000
  0.8073722568280335  0.5380862835047558  0.0000000000000000
  1.1407055901600027  0.7380862835036190  0.0000000000000000
  0.4740389234929903  0.7380862835036190  0.0000000000000000
  0.8073722568260038  0.7380862835036190  0.0000000000000000
  0.1407055901599787 -0.0619137164962383  0.0000000000000000
  0.4740389234930017 -0.0619137164962250  0.0000000000000000
  0.8073722568260133 -0.0619137164962383  0.0000000000000000
  0.3201838529240253  0.1920708306438992  0.0159535054489979
  0.6535171862570576  0.1920708306438992  0.0159535054489979
  0.9868505195880459  0.1920708306438992  0.0159535054489979
  0.3201838529240034  0.3920708306437910  0.0159535054489979
  0.6535171862570239  0.3920708306437910  0.0159535054489979
  0.9868505195890583  0.3920708306437910  0.0159535054489979
  0.3201838529239984  0.5920708306437140  0.0159535054489979
  0.6535171862569981  0.5920708306437140  0.0159535054489979
  0.9868505195900260  0.5920708306437140  0.0159535054489979
  0.3201838529240152 -0.2079291693551473  0.0159535054489979
  0.6535171862569974  0.7920708306435739  0.0159535054489979
```

| | | |
|---|---|---|
| 0.9868505195910328 | 0.7920708306415821 | 0.0159535054489979 |
| 0.3201838529239945 | -0.0079291693562827 | 0.0159535054489979 |
| 0.6535171862570184 | -0.0079291693562731 | 0.0159535054489979 |
| 0.9868505195890415 | -0.0079291693562827 | 0.0159535054489979 |
| 0.2186901232590162 | 0.0615218340179675 | 0.0955531191689971 |
| 0.5520234565921429 | 0.0615218340179675 | 0.0955531191689971 |
| 0.8853567899251421 | 0.0615218340179675 | 0.0955531191689971 |
| 0.2186901232590000 | 0.2615218340178746 | 0.0955531191689971 |
| 0.5520234565920035 | 0.2615218340178746 | 0.0955531191689971 |
| 0.8853567899250724 | 0.2615218340178746 | 0.0955531191689971 |
| 0.2186901232589941 | 0.4615218340177490 | 0.0955531191689971 |
| 0.5520234565920035 | 0.4615218340177490 | 0.0955531191689971 |
| 0.8853567899250371 | 0.4615218340177490 | 0.0955531191689971 |
| 1.2186901232590404 | 0.6615218340176605 | 0.0955531191689971 |
| 0.5520234565920034 | 0.6615218340176605 | 0.0955531191689971 |
| 0.8853567899250187 | 0.6615218340176605 | 0.0955531191689971 |
| 0.2186901232599966 | -0.1384781659821979 | 0.0955531191689971 |
| 0.5520234565920362 | -0.1384781659821979 | 0.0955531191689971 |
| 0.8853567899250195 | 0.8615218340175480 | 0.0955531191689971 |
| 1.0648350526889985 | 0.1155063811579354 | 0.1115066246180021 |
| 0.3981683860230350 | 0.1155063811579354 | 0.1115066246180021 |
| 0.7315017193541794 | 0.1155063811579354 | 0.1115066246180021 |
| 1.0648350526889983 | 0.3155063811578409 | 0.1115066246180021 |
| 0.3981683860230106 | 0.3155063811578409 | 0.1115066246180021 |
| 0.7315017193560484 | 0.3155063811578409 | 0.1115066246180021 |
| 1.0648350526889976 | 0.5155063811587531 | 0.1115066246180021 |
| 0.3981683860229997 | 0.5155063811587531 | 0.1115066246180021 |
| 0.7315017193560198 | 0.5155063811587531 | 0.1115066246180021 |
| 1.0648350526889974 | 0.7155063811576179 | 0.1115066246180021 |
| 0.3981683860229910 | 0.7155063811576179 | 0.1115066246180021 |
| 0.7315017193560153 | 0.7155063811576179 | 0.1115066246180021 |
| 1.0648350526889967 | 0.9155063811574945 | 0.1115066246180021 |
| 0.3981683860230087 | -0.0844936188422338 | 0.1115066246180021 |
| 0.7315017193560174 | -0.0844936188422357 | 0.1115066246180021 |
| 0.6304084736507563 | 0.5857379554536319 | 0.1927393063896570 |
| 0.9636407171082978 | 0.3847475050089888 | 0.1925581040365705 |
| 0.6301528171157934 | 0.3853902859552412 | 0.1936114508164705 |
| 0.6299664886794580 | 0.7849852036888845 | 0.1928336524150752 |
| 0.9636618374409234 | 0.7850182433317834 | 0.1927874349438946 |
| 0.6304164720753073 | -0.0147018799422408 | 0.1929555372104677 |
| 0.9632363821141557 | 0.5849997456277949 | 0.1929798243877321 |
| 0.9638275253570283 | 0.1851302199772448 | 0.1927449360368020 |
| 0.9635396101552917 | -0.0151287064945853 | 0.1929068257067405 |
| 0.2967908548044615 | 0.5851582222224950 | 0.1927857687518814 |

| | | |
|---|---|---|
| 0.2966473175895319 | -0.2150815368878819 | 0.1928725048539640 |
| 0.2967058134048821 | -0.0150559467319497 | 0.1928303648845178 |
| 0.6299788916313072 | 0.1847721191087778 | 0.1923362171480774 |
| 0.2964940789345508 | 0.1850923136870772 | 0.1928433192397213 |
| 0.2966336835006465 | 0.3848221568800858 | 0.1928822805910106 |
| 0.8091198332631980 | 0.2389015074667922 | 0.2082835037121988 |
| 1.1434540208169708 | 0.8384705790538409 | 0.2080372856723930 |
| 0.4766163680861934 | -0.1612553422559228 | 0.2079771967098759 |
| 0.8099900513632897 | 0.8386158911145039 | 0.2081685971672499 |
| 0.8102404657486370 | 0.0387057100856688 | 0.2084281292798948 |
| 0.1434021559992255 | 0.0384059198567651 | 0.2082854261273203 |
| 1.1433376420100552 | 0.6383958364547405 | 0.2083415737083080 |
| 1.1434634806079020 | 0.4385626327916113 | 0.2080610682072468 |
| 0.8097913651676885 | 0.4380796160717032 | 0.2087982533062967 |
| 0.4769965398244478 | 0.0393369824594099 | 0.2085586704758794 |
| 0.4767427866662864 | 0.6382971773978089 | 0.2080785338433908 |
| 0.1427447550905517 | 0.2380928047674210 | 0.2084573852280727 |
| 0.4771787286031654 | 0.2387767937571151 | 0.2081907391871118 |
| 0.8089478174389756 | 0.6374582528600172 | 0.2086332260218242 |
| 0.4771980726196628 | 0.4383830325896686 | 0.2082819690370244 |
| 0.3718242854364556 | 0.7076283051582655 | 0.2856176080375666 |
| 0.7055723518148846 | 0.1073397752982830 | 0.2858588211926913 |
| 0.7054007887199953 | 0.3097209030076912 | 0.2887234830874907 |
| 0.3714474992680664 | 0.1075374210450047 | 0.2857737854073520 |
| 1.0396054638341710 | 0.3071389532998199 | 0.2857979933289453 |
| 0.7051981364840095 | -0.0922239189995881 | 0.2857888499716245 |
| 1.0390423042821193 | 0.9072219619275317 | 0.2857448784018380 |
| 1.0383382706820996 | 0.7074447538239915 | 0.2857679402647829 |
| 1.0390897004533575 | 0.5070701903057361 | 0.2857626174377179 |
| 0.3719694287140339 | -0.0925767864612620 | 0.2857168864639016 |
| 1.0384264204365647 | 0.1070726193900800 | 0.2858454036627179 |
| 0.7056489374443260 | 0.7089239620394304 | 0.2856276886038714 |
| 0.7058299001284039 | 0.5096447934805204 | 0.2861312497671983 |
| 0.3703336372468960 | 0.3068812747002752 | 0.2853252333098870 |
| 0.3715423975944840 | 0.5073315376254613 | 0.2857764574025222 |
| 0.8887254019447531 | 0.3616637193874629 | 0.2986371651063892 |
| 0.5533872040569132 | 0.7624738101469044 | 0.2991971971599379 |
| 0.8876058259620476 | 0.5617746591713944 | 0.2993726428770911 |
| 0.5535448793691992 | -0.0382518063779137 | 0.2990204177014523 |
| 0.2203573415888274 | 0.5621688851187078 | 0.2992195785424265 |
| 0.2196612240245009 | 0.1614457834452352 | 0.2994887270961154 |
| 0.8870465319703394 | 0.1620026857485666 | 0.2990567095619088 |
| 0.8872369271413351 | 0.7626755579099532 | 0.2992707264202833 |
| 0.2199876732719797 | -0.2379375847909744 | 0.2993243440967101 |

| | | |
|---|---|---|
| 0.2205210384903220 | -0.0380249194251775 | 0.2992828403125964 |
| 0.2200864758766914 | 0.3621013113929121 | 0.2995081136640092 |
| 0.8871079884409051 | -0.0384340623149632 | 0.2993536813872186 |
| 0.5519786625201589 | 0.5638425355265927 | 0.2988951500159894 |
| 0.5518830067613022 | 0.1589161966346404 | 0.2989434471805200 |
| 0.5483207197267426 | 0.3625887389519763 | 0.2998457134188383 |

Ru: Final state for $CH_3O$ and CO coupling

($E$ = -1129.177 eV, $ZPE$ = 1.25463467 eV, $S*T$ = 0.668489 eV)

%BLOCK LATTICE_CART

| | | |
|---|---|---|
| 15.122801570000000 | 0.000000000000000 | 0.000000000000000 |
| -3.641814920000000 | 13.056846300000004 | 0.000000000000000 |
| 0.000000000000000 | 0.000000000000000 | 21.508856710000003 |

Selective dynamics

Direct

| | | | |
|---|---|---|---|
| H | 0.7859517546959999 | 0.3430194796070000 | 0.4446242190879998 |
| H | 0.6850391697539999 | 0.3858915917069999 | 0.4618291475749999 |
| H | 0.6893493391219998 | 0.2596540007950000 | 0.4846255141279998 |
| C | 0.5767946624469997 | 0.2462747162779999 | 0.3820069673429999 |
| C | 0.7112393689099998 | 0.3203705111299999 | 0.4497067678839999 |
| O | 0.6753541298219999 | 0.2781086112200000 | 0.3897989861609999 |
| O | 0.5279564679659999 | 0.2613069709170000 | 0.4241850729359999 |
| Ru | 0.1407055901600000 | 0.1380862835040000 | 0.0000000000000000 |
| Ru | 0.4740389234929999 | 0.1380862835040000 | 0.0000000000000000 |
| Ru | 0.8073722568249998 | 0.1380862835040000 | 0.0000000000000000 |
| Ru | 0.1407055901590000 | 0.3380862835039999 | 0.0000000000000000 |
| Ru | 0.4740389234929999 | 0.3380862835039999 | 0.0000000000000000 |
| Ru | 0.8073722568260010 | 0.3380862835039999 | 0.0000000000000000 |
| Ru | 0.1407055901600000 | 0.5380862835049999 | 0.0000000000000000 |
| Ru | 0.4740389234929998 | 0.5380862835049999 | 0.0000000000000000 |
| Ru | 0.8073722568279997 | 0.5380862835049999 | 0.0000000000000000 |
| Ru | 0.1407055901600000 | 0.7380862835039999 | 0.0000000000000000 |
| Ru | 0.4740389234929999 | 0.7380862835039999 | 0.0000000000000000 |
| Ru | 0.8073722568259999 | 0.7380862835039999 | 0.0000000000000000 |
| Ru | 0.1407055901600000 | 0.9380862835039999 | 0.0000000000000000 |
| Ru | 0.4740389234929999 | 0.9380862835039999 | 0.0000000000000000 |
| Ru | 0.8073722568259999 | 0.9380862835039999 | 0.0000000000000000 |
| Ru | 0.3201838529239999 | 0.1920708306440000 | 0.0159535054490000 |
| Ru | 0.6535171862569997 | 0.1920708306440000 | 0.0159535054490000 |
| Ru | 0.9868505195879999 | 0.1920708306440000 | 0.0159535054490000 |
| Ru | 0.3201838529239999 | 0.3920708306440000 | 0.0159535054490000 |
| Ru | 0.6535171862569997 | 0.3920708306440000 | 0.0159535054490000 |
| Ru | 0.9868505195889999 | 0.3920708306440000 | 0.0159535054490000 |
| Ru | 0.3201838529239999 | 0.5920708306439999 | 0.0159535054490000 |
| Ru | 0.6535171862569997 | 0.5920708306439999 | 0.0159535054490000 |
| Ru | 0.9868505195899999 | 0.5920708306439999 | 0.0159535054490000 |
| Ru | 0.3201838529239999 | 0.7920708306449998 | 0.0159535054490000 |
| Ru | 0.6535171862569997 | 0.7920708306439999 | 0.0159535054490000 |
| Ru | 0.9868505195909998 | 0.7920708306419999 | 0.0159535054490000 |
| Ru | 0.3201838529239999 | 0.9920708306439999 | 0.0159535054490000 |
| Ru | 0.6535171862569997 | 0.9920708306439999 | 0.0159535054490000 |

| | | | |
|---|---|---|---|
| Ru | 0.9868505195889999 | 0.9920708306439999 | 0.0159535054490000 |
| Ru | 0.2186901232590000 | 0.0615218340179999 | 0.0955531191690000 |
| Ru | 0.5520234565919998 | 0.0615218340179999 | 0.0955531191690000 |
| Ru | 0.8853567899249998 | 0.0615218340179999 | 0.0955531191690000 |
| Ru | 0.2186901232590000 | 0.2615218340179999 | 0.0955531191690000 |
| Ru | 0.5520234565919999 | 0.2615218340179999 | 0.0955531191690000 |
| Ru | 0.8853567899249998 | 0.2615218340179999 | 0.0955531191690000 |
| Ru | 0.2186901232590000 | 0.4615218340180000 | 0.0955531191690000 |
| Ru | 0.5520234565919999 | 0.4615218340180000 | 0.0955531191690000 |
| Ru | 0.8853567899249998 | 0.4615218340180000 | 0.0955531191690000 |
| Ru | 0.2186901232590000 | 0.6615218340179999 | 0.0955531191690000 |
| Ru | 0.5520234565919999 | 0.6615218340179999 | 0.0955531191690000 |
| Ru | 0.8853567899249999 | 0.6615218340179999 | 0.0955531191690000 |
| Ru | 0.2186901232599999 | 0.8615218340179998 | 0.0955531191690000 |
| Ru | 0.5520234565919999 | 0.8615218340179998 | 0.0955531191690000 |
| Ru | 0.8853567899249999 | 0.8615218340179998 | 0.0955531191690000 |
| Ru | 0.0648350526890003 | 0.1155063811580000 | 0.1115066246180000 |
| Ru | 0.3981683860229999 | 0.1155063811580000 | 0.1115066246180000 |
| Ru | 0.7315017193540009 | 0.1155063811580000 | 0.1115066246180000 |
| Ru | 0.0648350526890000 | 0.3155063811580000 | 0.1115066246180000 |
| Ru | 0.3981683860229999 | 0.3155063811580000 | 0.1115066246180000 |
| Ru | 0.7315017193559997 | 0.3155063811580000 | 0.1115066246180000 |
| Ru | 0.0648350526890000 | 0.5155063811589999 | 0.1115066246180000 |
| Ru | 0.3981683860229999 | 0.5155063811589999 | 0.1115066246180000 |
| Ru | 0.7315017193559997 | 0.5155063811589999 | 0.1115066246180000 |
| Ru | 0.0648350526890000 | 0.7155063811579999 | 0.1115066246180000 |
| Ru | 0.3981683860229999 | 0.7155063811579999 | 0.1115066246180000 |
| Ru | 0.7315017193559997 | 0.7155063811579999 | 0.1115066246180000 |
| Ru | 0.0648350526890000 | 0.9155063811569998 | 0.1115066246180000 |
| Ru | 0.3981683860229999 | 0.9155063811579998 | 0.1115066246180000 |
| Ru | 0.7315017193559997 | 0.9155063811579998 | 0.1115066246180000 |
| Ru | 0.2964675081359999 | 0.1847051192070000 | 0.1927997771510000 |
| Ru | 0.9633327335179998 | 0.1852154134539999 | 0.1927200849069999 |
| Ru | 0.9636890881299998 | 0.5851539320559999 | 0.1927980265580000 |
| Ru | 0.9639541430309998 | 0.3851446849819999 | 0.1925034601739999 |
| Ru | 0.6304016511879999 | 0.5851570974459999 | 0.1929919310060000 |
| Ru | 0.2967585528219999 | 0.5849936160249999 | 0.1928403392820000 |
| Ru | 0.6300473315159999 | 0.7850729630269998 | 0.1929523052029999 |
| Ru | 0.9632451393969996 | 0.9850633143499999 | 0.1928461022069999 |
| Ru | 0.2962069616110000 | 0.9851463950099999 | 0.1928046674549999 |
| Ru | 0.6301350017600000 | 0.9853368894139999 | 0.1931047237250000 |
| Ru | 0.2969371784300000 | 0.3850392607900000 | 0.1930096639049999 |
| Ru | 0.2967747222320000 | 0.7848699476799999 | 0.1929328055439999 |
| Ru | 0.9633634259739998 | 0.7850801612279998 | 0.1928828545559999 |

| | | | |
|---|---|---|---|
| Ru | 0.6299918592809998 | 0.1849082702970000 | 0.1936440368560000 |
| Ru | 0.6302771117160000 | 0.3848642812520000 | 0.1937453483709999 |
| Ru | 0.8083809416109998 | 0.2390415317470000 | 0.2088090620080000 |
| Ru | 0.4771557148049999 | 0.2388915930949999 | 0.2082462798030000 |
| Ru | 0.8088340346659989 | 0.4373733414859999 | 0.2087636984289999 |
| Ru | 0.1434416959190000 | 0.4383576433340000 | 0.2082400003740000 |
| Ru | 0.4767291392079999 | 0.6383632186639999 | 0.2080337432340000 |
| Ru | 0.4768193692020000 | 0.8385156108629998 | 0.2082164611539999 |
| Ru | 0.8097640347689998 | 0.8384516740389999 | 0.2083480660210000 |
| Ru | 0.1433077529010000 | 0.8382063936269999 | 0.2082663765539999 |
| Ru | 0.1430582585640000 | 0.0385775369360000 | 0.2082398709330000 |
| Ru | 0.8102607665709999 | 0.0385112994229999 | 0.2080702620550000 |
| Ru | 0.8102828805279999 | 0.6387478564910000 | 0.2080640175600000 |
| Ru | 0.4756455137469999 | 0.0372729039569999 | 0.2068174557109999 |
| Ru | 0.4771334346739999 | 0.4383802151679999 | 0.2081982931019999 |
| Ru | 0.1434760271110000 | 0.6385639619469999 | 0.2082277197269999 |
| Ru | 0.1432668425970000 | 0.2383699139950000 | 0.2084333332880000 |
| Ru | 0.3704613885370000 | 0.3067314568490000 | 0.2852422806000000 |
| Ru | 0.3713362788219998 | 0.1068399745700000 | 0.2854216515879999 |
| Ru | 0.3722572725369999 | 0.7074329361349999 | 0.2857147044219999 |
| Ru | 0.7051657422819999 | 0.9075665974419999 | 0.2858761409630000 |
| Ru | 0.0386568307099999 | 0.9075460826319999 | 0.2856927668089999 |
| Ru | 0.7056061152359999 | 0.1060778127720000 | 0.2857949033450000 |
| Ru | 0.0390829545800000 | 0.3071596869590000 | 0.2857630996689999 |
| Ru | 0.3720170182199999 | 0.5075441587619999 | 0.2857172863160000 |
| Ru | 0.7061166992099999 | 0.5094330712999999 | 0.2857290100000000 |
| Ru | 0.7053052178559998 | 0.7076419674779999 | 0.2859882547370000 |
| Ru | 0.3722898460660000 | 0.9078284768099999 | 0.2854530552949999 |
| Ru | 0.0382911289760002 | 0.1071238293100000 | 0.2858039003419999 |
| Ru | 0.0394583329069999 | 0.5078649863379999 | 0.2857871745869999 |
| Ru | 0.0388444831949999 | 0.7075286638779998 | 0.2859310002920000 |
| Ru | 0.7082089906110000 | 0.3083553338360000 | 0.2883364022250000 |
| Ru | 0.8870397142790009 | 0.1620740673490000 | 0.2989640296120000 |
| Ru | 0.8891852244539997 | 0.3628574881869999 | 0.2985862112799999 |
| Ru | 0.8875139006189999 | 0.5624115513729999 | 0.2990792526130000 |
| Ru | 0.5537854701499998 | 0.5626864745899999 | 0.2990614859549999 |
| Ru | 0.5533791632510000 | 0.9621103759849999 | 0.2991019323090000 |
| Ru | 0.2202680446660000 | 0.3622337875619999 | 0.2992843474219999 |
| Ru | 0.2205855344029999 | 0.5621955281799999 | 0.2993334036670000 |
| Ru | 0.5534288079619999 | 0.7625222076560000 | 0.2992700140640000 |
| Ru | 0.2205077391840000 | 0.7620212072379999 | 0.2992373042520000 |
| Ru | 0.2200367964290000 | 0.9613403549829998 | 0.2994354294570000 |
| Ru | 0.2197580103529999 | 0.1614841024960000 | 0.2992263445239999 |
| Ru | 0.8869024008309998 | 0.7617114134229999 | 0.2993129789050000 |

| | | | |
|---|---|---|---|
| Ru | 0.8866550918619996 | 0.9621376883689999 | 0.2993380672470000 |
| Ru | 0.5510777995850000 | 0.3640315762460000 | 0.2998827148209999 |
| Ru | 0.5500631914009999 | 0.1604882951000000 | 0.3009321175109999 |

Ru: Initial state for $CH_3OCO$ and styrene coupling ($ArCHCH_2(COOMe)$)
($E$ = -1233.6925 eV, $ZPE$ = 4.79911640 eV, $S*T$ = 1.2505961376379 eV)
%BLOCK LATTICE_CART

| | | |
|---|---|---|
| 15.122801570000000 | 0.000000000000000 | 0.000000000000000 |
| -3.641814920000000 | 13.056846300000004 | 0.000000000000000 |
| 0.000000000000000 | 0.000000000000000 | 21.508856710000003 |

Selective dynamics
Direct

| | | | |
|---|---|---|---|
| H | 0.6534894872510000 | 0.5736236144929998 | 0.4012925786339999 |
| H | 0.5044389581249998 | 0.5706725308209999 | 0.4032032457649998 |
| H | 0.0794673524639999 | 0.7823276246839997 | 0.4208704015199998 |
| H | 0.4465560082089999 | 0.2415413631299999 | 0.4025093016429999 |
| H | 0.2831818524959999 | 0.2285903175640000 | 0.4046616421249998 |
| H | 0.3438752047299999 | 0.5563082475379999 | 0.4087869471609998 |
| H | 0.7309723363070001 | 0.4947061402449999 | 0.4083871473409999 |
| H | 0.2332981377030000 | 0.3869711425060000 | 0.4196319600689998 |
| H | 0.9950401497629998 | 0.8438674732489998 | 0.4474080752459999 |
| H | 0.6029880772179999 | 0.3431059717689999 | 0.4214291972599998 |
| H | 0.0184696840760000 | 0.7411271625019998 | 0.4924266026899998 |
| C | 0.9635495167779999 | 0.6337698162369999 | 0.3708356324429997 |
| C | 0.4605371686339999 | 0.4997182589049998 | 0.3826194661009998 |
| C | 0.3625621626250000 | 0.4913796522289999 | 0.3877068608499999 |
| C | 0.3283832571660000 | 0.3008998028650000 | 0.3873199821729998 |
| C | 0.6660148402849999 | 0.5001203098129999 | 0.3879144588549999 |
| C | 0.4253421719749999 | 0.3089730979890000 | 0.3882937755089999 |
| C | 0.4939429834369999 | 0.4072766741320000 | 0.3889824421219998 |
| C | 0.2938751969000000 | 0.3917393090590000 | 0.3902700581999999 |
| C | 0.5893339572959999 | 0.4084099768510000 | 0.3972190430659999 |
| C | 0.0141291246790001 | 0.7714986385469999 | 0.4456317337799998 |
| O | 0.0437445188460000 | 0.6152544206679999 | 0.3763980170199998 |
| O | 0.9399893574449998 | 0.7006463347109999 | 0.4113199711259998 |
| Ru | 0.1407055901600000 | 0.1380862835039999 | 0.0000000000000000 |
| Ru | 0.4740389234930003 | 0.1380862835039999 | 0.0000000000000000 |
| Ru | 0.8073722568259998 | 0.1380862835039999 | 0.0000000000000000 |
| Ru | 0.1407055901600000 | 0.3380862835039999 | 0.0000000000000000 |
| Ru | 0.4740389234929999 | 0.3380862835039999 | 0.0000000000000000 |
| Ru | 0.8073722568260009 | 0.3380862835039999 | 0.0000000000000000 |
| Ru | 0.1407055901600000 | 0.5380862835039999 | 0.0000000000000000 |
| Ru | 0.4740389234929999 | 0.5380862835039999 | 0.0000000000000000 |
| Ru | 0.8073722568259999 | 0.5380862835039999 | 0.0000000000000000 |
| Ru | 0.1407055901600000 | 0.7380862835039999 | 0.0000000000000000 |
| Ru | 0.4740389234930000 | 0.7380862835039999 | 0.0000000000000000 |
| Ru | 0.8073722568259999 | 0.7380862835039999 | 0.0000000000000000 |
| Ru | 0.1407055901599998 | 0.9380862835039996 | 0.0000000000000000 |

| | | | |
|---|---|---|---|
| Ru | 0.4740389234929998 | 0.9380862835039996 | 0.0000000000000000 |
| Ru | 0.8073722568259998 | 0.9380862835039996 | 0.0000000000000000 |
| Ru | 0.3201838529239999 | 0.1920708306440000 | 0.0159535054490000 |
| Ru | 0.6535171862569995 | 0.1920708306440000 | 0.0159535054490000 |
| Ru | 0.9868505195899998 | 0.1920708306440000 | 0.0159535054490000 |
| Ru | 0.3201838529239999 | 0.3920708306440000 | 0.0159535054490000 |
| Ru | 0.6535171862569995 | 0.3920708306440000 | 0.0159535054490000 |
| Ru | 0.9868505195899998 | 0.3920708306440000 | 0.0159535054490000 |
| Ru | 0.3201838529239999 | 0.5920708306439999 | 0.0159535054490000 |
| Ru | 0.6535171862569997 | 0.5920708306439999 | 0.0159535054490000 |
| Ru | 0.9868505195899999 | 0.5920708306439999 | 0.0159535054490000 |
| Ru | 0.3201838529239999 | 0.7920708306439999 | 0.0159535054490000 |
| Ru | 0.6535171862569997 | 0.7920708306439999 | 0.0159535054490000 |
| Ru | 0.9868505195900000 | 0.7920708306439999 | 0.0159535054490000 |
| Ru | 0.3201838529239999 | 0.9920708306439996 | 0.0159535054490000 |
| Ru | 0.6535171862569995 | 0.9920708306439996 | 0.0159535054490000 |
| Ru | 0.9868505195899999 | 0.9920708306439996 | 0.0159535054490000 |
| Ru | 0.2186901232589999 | 0.0615218340179999 | 0.0955531191690000 |
| Ru | 0.5520234565919998 | 0.0615218340179999 | 0.0955531191690000 |
| Ru | 0.8853567899249998 | 0.0615218340179999 | 0.0955531191690000 |
| Ru | 0.2186901232590000 | 0.2615218340179999 | 0.0955531191690000 |
| Ru | 0.5520234565919999 | 0.2615218340179999 | 0.0955531191690000 |
| Ru | 0.8853567899249998 | 0.2615218340179999 | 0.0955531191690000 |
| Ru | 0.2186901232590000 | 0.4615218340180000 | 0.0955531191690000 |
| Ru | 0.5520234565919999 | 0.4615218340180000 | 0.0955531191690000 |
| Ru | 0.8853567899249997 | 0.4615218340180000 | 0.0955531191690000 |
| Ru | 0.2186901232589999 | 0.6615218340179999 | 0.0955531191690000 |
| Ru | 0.5520234565919999 | 0.6615218340179999 | 0.0955531191690000 |
| Ru | 0.8853567899249999 | 0.6615218340179999 | 0.0955531191690000 |
| Ru | 0.2186901232589999 | 0.8615218340179998 | 0.0955531191690000 |
| Ru | 0.5520234565919999 | 0.8615218340179998 | 0.0955531191690000 |
| Ru | 0.8853567899250000 | 0.8615218340179998 | 0.0955531191690000 |
| Ru | 0.0648350526890002 | 0.1155063811579999 | 0.1115066246180000 |
| Ru | 0.3981683860229999 | 0.1155063811579999 | 0.1115066246180000 |
| Ru | 0.7315017193559996 | 0.1155063811579999 | 0.1115066246180000 |
| Ru | 0.0648350526890000 | 0.3155063811580000 | 0.1115066246180000 |
| Ru | 0.3981683860229999 | 0.3155063811580000 | 0.1115066246180000 |
| Ru | 0.7315017193559996 | 0.3155063811580000 | 0.1115066246180000 |
| Ru | 0.0648350526890000 | 0.5155063811579999 | 0.1115066246180000 |
| Ru | 0.3981683860229999 | 0.5155063811579999 | 0.1115066246180000 |
| Ru | 0.7315017193559995 | 0.5155063811579999 | 0.1115066246180000 |
| Ru | 0.0648350526890000 | 0.7155063811579999 | 0.1115066246180000 |
| Ru | 0.3981683860229999 | 0.7155063811579999 | 0.1115066246180000 |
| Ru | 0.7315017193559995 | 0.7155063811579999 | 0.1115066246180000 |

| | | | |
|---|---|---|---|
| Ru | 0.0648350526890000 | 0.9155063811579998 | 0.1115066246180000 |
| Ru | 0.3981683860229999 | 0.9155063811579998 | 0.1115066246180000 |
| Ru | 0.7315017193559996 | 0.9155063811579998 | 0.1115066246180000 |
| Ru | 0.6309615284999999 | 0.5842235810920000 | 0.1917984884509999 |
| Ru | 0.6304473129929999 | 0.1853314624539998 | 0.1925689265709999 |
| Ru | 0.6311904204410002 | 0.7858928048559999 | 0.1926227586449999 |
| Ru | 0.6298595863260004 | 0.3850878493720000 | 0.1925338692729999 |
| Ru | 0.9634526574120002 | 0.1857356186309997 | 0.1929241372879999 |
| Ru | 0.9644323567289999 | 0.7854237458919998 | 0.1933883998519999 |
| Ru | 0.9638884700549999 | 0.3859766977099999 | 0.1932553027530000 |
| Ru | 0.9629144248359998 | 0.5841651778870000 | 0.1936174983740000 |
| Ru | 0.2975072529369999 | 0.7851422650639999 | 0.1923768827269999 |
| Ru | 0.6305935346350002 | 0.9852124394929998 | 0.1927357361359999 |
| Ru | 0.2975893674359998 | 0.5850930212729999 | 0.1927594891399999 |
| Ru | 0.9637861385290000 | 0.9852924745499998 | 0.1930392237659999 |
| Ru | 0.2975945210249997 | 0.9853070767479998 | 0.1928909581140000 |
| Ru | 0.2964313298540000 | 0.1846294008400000 | 0.1933143875150000 |
| Ru | 0.2963909145120000 | 0.3855228707309998 | 0.1941729539509999 |
| Ru | 0.4772309570639999 | 0.8387028382459998 | 0.2078893813149999 |
| Ru | 0.8106455898159999 | 0.0387560941359997 | 0.2078856102219999 |
| Ru | 0.4774091794769999 | 0.0385116097709999 | 0.2080559371950000 |
| Ru | 0.4753127409769999 | 0.4378061532069998 | 0.2081057181829999 |
| Ru | 0.1428519072419998 | 0.8385456485839997 | 0.2078747890619999 |
| Ru | 0.8101979669629997 | 0.2388247372839999 | 0.2085595983830000 |
| Ru | 0.1437564336840000 | 0.2397162282920000 | 0.2084620878839999 |
| Ru | 0.4765356344039998 | 0.2393678763380000 | 0.2080496774890000 |
| Ru | 0.1438213654049998 | 0.6373828175309999 | 0.2084950890309999 |
| Ru | 0.8112017697249998 | 0.8380381519629997 | 0.2091702040050000 |
| Ru | 0.1422861382360000 | 0.4398558529399999 | 0.2094145603460000 |
| Ru | 0.8094267928569998 | 0.6385910246069999 | 0.2083537786649999 |
| Ru | 0.1436917162110000 | 0.0392126252279998 | 0.2086789589459999 |
| Ru | 0.4774868219599999 | 0.6368995034539999 | 0.2087382058409999 |
| Ru | 0.8083448157039999 | 0.4367866357759999 | 0.2068999249629998 |
| Ru | 0.3731320574720000 | 0.1051979645870000 | 0.2847733410880000 |
| Ru | 0.3734851930610000 | 0.7097006194419999 | 0.2854858074530000 |
| Ru | 0.7056000041139999 | 0.7104091149289998 | 0.2854934859399999 |
| Ru | 0.0384206959910000 | 0.3068906083740000 | 0.2857408266099998 |
| Ru | 0.7060201637159999 | 0.1080075262669999 | 0.2857803186589999 |
| Ru | 0.7058445020469998 | 0.3059053900179999 | 0.2854918658699999 |
| Ru | 0.3723752555039999 | 0.9073512866589999 | 0.2856942052909999 |
| Ru | 0.0381223583840000 | 0.1092875246999998 | 0.2859756577209998 |
| Ru | 0.0386230308089999 | 0.5073534435039999 | 0.2894095410719999 |
| Ru | 0.0413338826239999 | 0.7087616201119996 | 0.2855996982410000 |
| Ru | 0.7061522376130003 | 0.9085148612349998 | 0.2858699172119998 |

| | | | |
|---|---|---|---|
| Ru | 0.0393851069190001 | 0.9095645984989996 | 0.2859555272729998 |
| Ru | 0.3735083096660000 | 0.5096220389439999 | 0.2877828128000000 |
| Ru | 0.3679785213460000 | 0.3018689180479999 | 0.2871709872199999 |
| Ru | 0.7050215031209999 | 0.5076551832379997 | 0.2885287617859999 |
| Ru | 0.5551691531289997 | 0.1599793815499999 | 0.2983609061009999 |
| Ru | 0.5545732492900003 | 0.7652155591239997 | 0.2986491331029998 |
| Ru | 0.8825264694919996 | 0.5612703805789999 | 0.3007314430149999 |
| Ru | 0.2204491168409999 | 0.5666827565349999 | 0.2980378287659999 |
| Ru | 0.8851659574859999 | 0.3612451231559999 | 0.2990896412230000 |
| Ru | 0.8856255272559999 | 0.7648862608499998 | 0.2998076124130000 |
| Ru | 0.2226980512110000 | 0.7637285973129998 | 0.2978677150279999 |
| Ru | 0.2203955572709998 | 0.9609169355319995 | 0.2990633137689999 |
| Ru | 0.5542573062469998 | 0.9627505310309995 | 0.2992860578009999 |
| Ru | 0.8878531861309998 | 0.9637283281019999 | 0.2994239784459999 |
| Ru | 0.8866267905799996 | 0.1627627544650000 | 0.2995726708119999 |
| Ru | 0.2172221842279999 | 0.1546158717610000 | 0.2999837435150000 |
| Ru | 0.5557662497849999 | 0.5694122668049999 | 0.2998301792629999 |
| Ru | 0.5512698240469999 | 0.3585426761480000 | 0.3024291264159999 |
| Ru | 0.2161853228640000 | 0.3639145971050000 | 0.3026405199250000 |

Ru: Transition state for $CH_3OCO$ and styrene coupling ($ArCHCH_2(COOMe)$)
($E$ = -1232.5635 eV, $ZPE$ = 4.75357629249999 eV, $S*T$ = 1.37493981256008 eV)
%BLOCK LATTICE_CART

| | | |
|---|---|---|
| 15.122801570000000 | 0.000000000000000 | 0.000000000000000 |
| -3.641814920000000 | 13.056846300000004 | 0.000000000000000 |
| 0.000000000000000 | 0.000000000000000 | 21.508856710000003 |

Selective dynamics
Direct

| | | | |
|---|---|---|---|
| H | 0.4976854605669999 | 0.5566786847319999 | 0.3997197298649999 |
| H | 0.4182028031130000 | 0.2257177761090000 | 0.4017339132579999 |
| H | 0.2567436795570000 | 0.2247048153470000 | 0.4038817967119999 |
| H | 0.3385956740259999 | 0.5548154391679999 | 0.4095281053999999 |
| H | 0.7582665057919997 | 0.8016384366609999 | 0.4338851136809999 |
| H | 0.8545686537859998 | 0.7506044436459999 | 0.4180567843690000 |
| H | 0.2192685332390000 | 0.3902937435909999 | 0.4220970487919999 |
| H | 0.5706864160379999 | 0.3077983401950000 | 0.4167769409350000 |
| H | 0.6222812429280000 | 0.5341514581799999 | 0.4436999745429998 |
| H | 0.7765919460119999 | 0.6966239034099999 | 0.4797902178559999 |
| H | 0.6917708126379998 | 0.4434826430459999 | 0.4534307156419998 |
| C | 0.7472642732019998 | 0.5704385697899999 | 0.3750639301439999 |
| C | 0.4480122336219999 | 0.4871769202449999 | 0.3811219403199999 |
| C | 0.3061487460619999 | 0.2960332876619999 | 0.3884975174279999 |
| C | 0.4008952376700000 | 0.2957727041889999 | 0.3880062319279999 |
| C | 0.3521050918609999 | 0.4869085073099999 | 0.3885737505429999 |
| C | 0.4745984360750000 | 0.3901651532079999 | 0.3871256095219999 |
| C | 0.2787292086870000 | 0.3906790241940000 | 0.3929232779259999 |
| C | 0.5666455272959999 | 0.3842010431469999 | 0.4028172051159999 |
| C | 0.7825104867679998 | 0.7325478480900000 | 0.4336326234049999 |
| C | 0.6410838426709999 | 0.4674404301949999 | 0.4252512099069999 |
| O | 0.7224532012039999 | 0.6631979975789999 | 0.3903800238619999 |
| O | 0.8259457985309998 | 0.5656997499249999 | 0.3959132838519999 |
| Ru | 0.1407055901600000 | 0.1380862835020000 | 0.0000000000000000 |
| Ru | 0.4740389234929999 | 0.1380862835040000 | 0.0000000000000000 |
| Ru | 0.8073722568269998 | 0.1380862835040000 | 0.0000000000000000 |
| Ru | 0.1407055901590000 | 0.3380862835039999 | 0.0000000000000000 |
| Ru | 0.4740389234929999 | 0.3380862835039999 | 0.0000000000000000 |
| Ru | 0.8073722568250000 | 0.3380862835039999 | 0.0000000000000000 |
| Ru | 0.1407055901600000 | 0.5380862835039999 | 0.0000000000000000 |
| Ru | 0.4740389234929999 | 0.5380862835039999 | 0.0000000000000000 |
| Ru | 0.8073722568259999 | 0.5380862835039999 | 0.0000000000000000 |
| Ru | 0.1407055901600000 | 0.7380862835039999 | 0.0000000000000000 |
| Ru | 0.4740389234919999 | 0.7380862835039999 | 0.0000000000000000 |
| Ru | 0.8073722568259999 | 0.7380862835039999 | 0.0000000000000000 |
| Ru | 0.1407055901600000 | 0.9380862835029999 | 0.0000000000000000 |

| | | | |
|---|---|---|---|
| Ru | 0.4740389234929999 | 0.9380862835029999 | 0.0000000000000000 |
| Ru | 0.8073722568249999 | 0.9380862835029999 | 0.0000000000000000 |
| Ru | 0.3201838529239999 | 0.1920708306440000 | 0.0159535054490000 |
| Ru | 0.6535171862559999 | 0.1920708306440000 | 0.0159535054490000 |
| Ru | 0.9868505195909998 | 0.1920708306440000 | 0.0159535054490000 |
| Ru | 0.3201838529239999 | 0.3920708306440000 | 0.0159535054490000 |
| Ru | 0.6535171862569997 | 0.3920708306440000 | 0.0159535054490000 |
| Ru | 0.9868505195899998 | 0.3920708306440000 | 0.0159535054490000 |
| Ru | 0.3201838529239999 | 0.5920708306439999 | 0.0159535054490000 |
| Ru | 0.6535171862569997 | 0.5920708306439999 | 0.0159535054490000 |
| Ru | 0.9868505195899999 | 0.5920708306439999 | 0.0159535054490000 |
| Ru | 0.3201838529239999 | 0.7920708306439999 | 0.0159535054490000 |
| Ru | 0.6535171862569997 | 0.7920708306439999 | 0.0159535054490000 |
| Ru | 0.9868505195899999 | 0.7920708306439999 | 0.0159535054490000 |
| Ru | 0.3201838529239999 | 0.9920708306439999 | 0.0159535054490000 |
| Ru | 0.6535171862569997 | 0.9920708306429998 | 0.0159535054490000 |
| Ru | 0.9868505195889999 | 0.9920708306429998 | 0.0159535054490000 |
| Ru | 0.2186901232590000 | 0.0615218340180000 | 0.0955531191690000 |
| Ru | 0.5520234565929999 | 0.0615218340180000 | 0.0955531191690000 |
| Ru | 0.8853567899239998 | 0.0615218340180000 | 0.0955531191690000 |
| Ru | 0.2186901232590000 | 0.2615218340179999 | 0.0955531191690000 |
| Ru | 0.5520234565919999 | 0.2615218340179999 | 0.0955531191690000 |
| Ru | 0.8853567899249998 | 0.2615218340179999 | 0.0955531191690000 |
| Ru | 0.2186901232590000 | 0.4615218340180000 | 0.0955531191690000 |
| Ru | 0.5520234565919999 | 0.4615218340180000 | 0.0955531191690000 |
| Ru | 0.8853567899249998 | 0.4615218340180000 | 0.0955531191690000 |
| Ru | 0.2186901232600000 | 0.6615218340179999 | 0.0955531191690000 |
| Ru | 0.5520234565919999 | 0.6615218340179999 | 0.0955531191690000 |
| Ru | 0.8853567899249999 | 0.6615218340179999 | 0.0955531191690000 |
| Ru | 0.2186901232589999 | 0.8615218340179998 | 0.0955531191690000 |
| Ru | 0.5520234565919999 | 0.8615218340179998 | 0.0955531191690000 |
| Ru | 0.8853567899249999 | 0.8615218340179998 | 0.0955531191690000 |
| Ru | 0.0648350526890000 | 0.1155063811580000 | 0.1115066246180000 |
| Ru | 0.3981683860229999 | 0.1155063811580000 | 0.1115066246180000 |
| Ru | 0.7315017193559998 | 0.1155063811580000 | 0.1115066246180000 |
| Ru | 0.0648350526890000 | 0.3155063811580000 | 0.1115066246180000 |
| Ru | 0.3981683860229999 | 0.3155063811580000 | 0.1115066246180000 |
| Ru | 0.7315017193549998 | 0.3155063811580000 | 0.1115066246180000 |
| Ru | 0.0648350526890000 | 0.5155063811579999 | 0.1115066246180000 |
| Ru | 0.3981683860229999 | 0.5155063811579999 | 0.1115066246180000 |
| Ru | 0.7315017193549999 | 0.5155063811579999 | 0.1115066246180000 |
| Ru | 0.0648350526890000 | 0.7155063811590000 | 0.1115066246180000 |
| Ru | 0.3981683860229999 | 0.7155063811590000 | 0.1115066246180000 |
| Ru | 0.7315017193559997 | 0.7155063811590000 | 0.1115066246180000 |

| | | | |
|---|---|---|---|
| Ru | 0.0648350526890000 | 0.9155063811579998 | 0.1115066246180000 |
| Ru | 0.3981683860229999 | 0.9155063811579998 | 0.1115066246180000 |
| Ru | 0.7315017193559997 | 0.9155063811579998 | 0.1115066246180000 |
| Ru | 0.9629918920629998 | 0.5843235054819999 | 0.1918752951150000 |
| Ru | 0.9635482541999998 | 0.7844032472009999 | 0.1923283223310000 |
| Ru | 0.9630166385819999 | 0.1850844101240000 | 0.1926674019940000 |
| Ru | 0.9626786273089998 | 0.3854903180770000 | 0.1928351265590000 |
| Ru | 0.6301397740069998 | 0.3844765382440000 | 0.1907967363710000 |
| Ru | 0.9638047877309998 | 0.9849372194999999 | 0.1927148259210000 |
| Ru | 0.6304750745539999 | 0.1853365478869999 | 0.1924148207030000 |
| Ru | 0.6305624858779999 | 0.5844405159719999 | 0.1924003703799999 |
| Ru | 0.2965735721020000 | 0.5852126796209999 | 0.1932434364610000 |
| Ru | 0.2968470116109999 | 0.7844969890529999 | 0.1929760354600000 |
| Ru | 0.2973314038509999 | 0.9850507627729999 | 0.1930408811909999 |
| Ru | 0.6310222997419999 | 0.7849724673169999 | 0.1932259676170000 |
| Ru | 0.6303759967279998 | 0.9845584048159998 | 0.1930247189169999 |
| Ru | 0.2971110192859999 | 0.1848917991080000 | 0.1937720733319999 |
| Ru | 0.2962662936570000 | 0.3851296436440000 | 0.1937260967810000 |
| Ru | 0.1433927412650000 | 0.8386353169029998 | 0.2078019665209999 |
| Ru | 0.4770066724869999 | 0.0379459827120000 | 0.2083113278129999 |
| Ru | 0.8104700060669998 | 0.0383495420340000 | 0.2080310282220000 |
| Ru | 0.1437299374690000 | 0.2386464713989999 | 0.2079135582340000 |
| Ru | 0.4769728375699999 | 0.8378992784139999 | 0.2080305101079999 |
| Ru | 0.8097624897449999 | 0.2381812639090000 | 0.2082966277259999 |
| Ru | 0.8087695898049999 | 0.4396959321519999 | 0.2097425744320000 |
| Ru | 0.1425519945150000 | 0.6376606604639999 | 0.2083875171260000 |
| Ru | 0.8098957823829998 | 0.6374579308189999 | 0.2097340396160000 |
| Ru | 0.1442445893809999 | 0.4386072573769999 | 0.2080762149079999 |
| Ru | 0.4763558352520000 | 0.6366736010609999 | 0.2086377734909999 |
| Ru | 0.1438892542740000 | 0.0390467646870000 | 0.2085005110110000 |
| Ru | 0.4767511457490000 | 0.2389750573510000 | 0.2085505938470000 |
| Ru | 0.8090538571139999 | 0.8371867911039997 | 0.2087779562360000 |
| Ru | 0.4753680006679999 | 0.4375167692759999 | 0.2086645876469999 |
| Ru | 0.3733182332139999 | 0.1054083694180000 | 0.2850778595930000 |
| Ru | 0.0385584708410000 | 0.3078031655110000 | 0.2851819716649999 |
| Ru | 0.7060496665259998 | 0.3059580155170000 | 0.2855527744970000 |
| Ru | 0.0348318097950000 | 0.5063401978390000 | 0.2855969375460000 |
| Ru | 0.3715516493459999 | 0.7087844605909999 | 0.2856996442190000 |
| Ru | 0.7055786834689999 | 0.1075669039090000 | 0.2857907512610000 |
| Ru | 0.0379514219430000 | 0.7065006858229999 | 0.2854359756650000 |
| Ru | 0.3717365180829999 | 0.9067394485399999 | 0.2857795711530000 |
| Ru | 0.0397927025789999 | 0.9079452028269998 | 0.2857032747280000 |
| Ru | 0.0381779554290000 | 0.1081460238060000 | 0.2857429041180000 |
| Ru | 0.7060671350479999 | 0.9085382111489998 | 0.2859127490960000 |

| | | | |
|---|---|---|---|
| Ru | 0.7046368388129998 | 0.5083742535029999 | 0.2901076452470000 |
| Ru | 0.3704253879289999 | 0.3026351742939999 | 0.2875058076859999 |
| Ru | 0.7055875697050000 | 0.7094023836319999 | 0.2881439385439999 |
| Ru | 0.3711094827229999 | 0.5085530504719998 | 0.2886494810949999 |
| Ru | 0.8900904092669998 | 0.7659700021299999 | 0.2979954584299999 |
| Ru | 0.5525641019479999 | 0.7640696751369999 | 0.2987056300209999 |
| Ru | 0.5549621726139999 | 0.1597803296660000 | 0.2984615558140000 |
| Ru | 0.2162452128039999 | 0.5653570873200000 | 0.2988094301870000 |
| Ru | 0.8881518281580000 | 0.9629833277659999 | 0.2989642535579999 |
| Ru | 0.2194770959310000 | 0.7627206331279999 | 0.2989976638760000 |
| Ru | 0.2203178530139999 | 0.9615728050099998 | 0.2989407804709999 |
| Ru | 0.8870421399379997 | 0.1619475934490000 | 0.2991662198030000 |
| Ru | 0.8864835628719999 | 0.3594974367150000 | 0.2996461619490000 |
| Ru | 0.5520627405699999 | 0.5668080045209999 | 0.2995195502660000 |
| Ru | 0.5539465101319999 | 0.9622335074069999 | 0.2993609023730000 |
| Ru | 0.2165662732830000 | 0.1549871810140000 | 0.3000366058139999 |
| Ru | 0.2147738409690000 | 0.3623918480059999 | 0.3019255343039999 |
| Ru | 0.8864187692479999 | 0.5614093607390001 | 0.3061961995060000 |
| Ru | 0.5536421480359999 | 0.3611207909349999 | 0.3012220327210000 |

Ru: Final state for $CH_3OCO$ and styrene coupling ($ArCHCH_2(COOMe)$)
($E$ = -1233.3688 eV, $ZPE$ = 4.82974809549999 eV, $S*T$ = 1.31179512432938 eV)
%BLOCK LATTICE_CART

| | | |
|---|---|---|
| 15.122801570000000 | 0.000000000000000 | 0.000000000000000 |
| -3.641814920000000 | 13.056846300000004 | 0.000000000000000 |
| 0.000000000000000 | 0.000000000000000 | 21.508856710000003 |

Selective dynamics
Direct

| | | | |
|---|---|---|---|
| H | 0.8672660413499999 | 0.7123294936919999 | 0.3909338863569999 |
| H | 0.4920467039129999 | 0.5607492965249999 | 0.3997887434890000 |
| H | 0.4265832059179999 | 0.2298413362150000 | 0.4017344666950000 |
| H | 0.8021114965059999 | 0.8009037073829999 | 0.4167180828859999 |
| H | 0.2633783329420000 | 0.2202884487719999 | 0.4026122623299999 |
| H | 0.3317886646239999 | 0.5502533470289999 | 0.4093188131659999 |
| H | 0.2186997211610000 | 0.3813401790479999 | 0.4220643937569999 |
| H | 0.5768177233920000 | 0.3242089746569999 | 0.4238614036119998 |
| H | 0.8110882955419999 | 0.6929190910769999 | 0.4639446487759999 |
| H | 0.6043000295079999 | 0.5455175750729999 | 0.4457080277969999 |
| H | 0.6777168409739998 | 0.4691523390959999 | 0.4663357168059999 |
| C | 0.7118086038449999 | 0.5504122054960000 | 0.3808322473969999 |
| C | 0.4454908798659999 | 0.4892467248599999 | 0.3815086623339999 |
| C | 0.3104909127880000 | 0.2934022875269999 | 0.3873830585939999 |
| C | 0.4064393934349999 | 0.2981228027810000 | 0.3878629872059999 |
| C | 0.3484718085519999 | 0.4840517065719999 | 0.3883605975409999 |
| C | 0.4768943790410000 | 0.3953581645390000 | 0.3885344920799999 |
| C | 0.2784286103140000 | 0.3853706573660000 | 0.3923302999350000 |
| C | 0.8049133583399999 | 0.7211090267529999 | 0.4166236299599999 |
| C | 0.5704462991329999 | 0.3956100700790000 | 0.4030192398290000 |
| C | 0.6399126461310000 | 0.4910409607549999 | 0.4271811416210000 |
| O | 0.7213642587839999 | 0.6628731484769999 | 0.3877493152429999 |
| O | 0.7910671080109999 | 0.5254447202639999 | 0.3790011900510000 |
| Ru | 0.1407055901600000 | 0.1380862835040000 | 0.0000000000000000 |
| Ru | 0.4740389234929999 | 0.1380862835040000 | 0.0000000000000000 |
| Ru | 0.8073722568259998 | 0.1380862835040000 | 0.0000000000000000 |
| Ru | 0.1407055901600000 | 0.3380862835039999 | 0.0000000000000000 |
| Ru | 0.4740389234929999 | 0.3380862835039999 | 0.0000000000000000 |
| Ru | 0.8073722568259999 | 0.3380862835039999 | 0.0000000000000000 |
| Ru | 0.1407055901600000 | 0.5380862835039999 | 0.0000000000000000 |
| Ru | 0.4740389234929999 | 0.5380862835039999 | 0.0000000000000000 |
| Ru | 0.8073722568259999 | 0.5380862835039999 | 0.0000000000000000 |
| Ru | 0.1407055901600000 | 0.7380862835039999 | 0.0000000000000000 |
| Ru | 0.4740389234929999 | 0.7380862835039999 | 0.0000000000000000 |
| Ru | 0.8073722568259999 | 0.7380862835039999 | 0.0000000000000000 |
| Ru | 0.1407055901600000 | 0.9380862835039999 | 0.0000000000000000 |

| | | | |
|---|---|---|---|
| Ru | 0.4740389234929999 | 0.9380862835039999 | 0.0000000000000000 |
| Ru | 0.8073722568259999 | 0.9380862835039999 | 0.0000000000000000 |
| Ru | 0.3201838529239999 | 0.1920708306440000 | 0.0159535054490000 |
| Ru | 0.6535171862569997 | 0.1920708306440000 | 0.0159535054490000 |
| Ru | 0.9868505195899998 | 0.1920708306440000 | 0.0159535054490000 |
| Ru | 0.3201838529239999 | 0.3920708306440000 | 0.0159535054490000 |
| Ru | 0.6535171862569997 | 0.3920708306440000 | 0.0159535054490000 |
| Ru | 0.9868505195899998 | 0.3920708306440000 | 0.0159535054490000 |
| Ru | 0.3201838529239999 | 0.5920708306439999 | 0.0159535054490000 |
| Ru | 0.6535171862569997 | 0.5920708306439999 | 0.0159535054490000 |
| Ru | 0.9868505195899999 | 0.5920708306439999 | 0.0159535054490000 |
| Ru | 0.3201838529239999 | 0.7920708306439999 | 0.0159535054490000 |
| Ru | 0.6535171862569997 | 0.7920708306439999 | 0.0159535054490000 |
| Ru | 0.9868505195899999 | 0.7920708306439999 | 0.0159535054490000 |
| Ru | 0.3201838529239999 | 0.9920708306439999 | 0.0159535054490000 |
| Ru | 0.6535171862569997 | 0.9920708306439999 | 0.0159535054490000 |
| Ru | 0.9868505195899999 | 0.9920708306439999 | 0.0159535054490000 |
| Ru | 0.2186901232590000 | 0.0615218340180000 | 0.0955531191690000 |
| Ru | 0.5520234565919998 | 0.0615218340180000 | 0.0955531191690000 |
| Ru | 0.8853567899249998 | 0.0615218340180000 | 0.0955531191690000 |
| Ru | 0.2186901232590000 | 0.2615218340179999 | 0.0955531191690000 |
| Ru | 0.5520234565919999 | 0.2615218340179999 | 0.0955531191690000 |
| Ru | 0.8853567899249998 | 0.2615218340179999 | 0.0955531191690000 |
| Ru | 0.2186901232590000 | 0.4615218340180000 | 0.0955531191690000 |
| Ru | 0.5520234565919999 | 0.4615218340180000 | 0.0955531191690000 |
| Ru | 0.8853567899249998 | 0.4615218340180000 | 0.0955531191690000 |
| Ru | 0.2186901232590000 | 0.6615218340179999 | 0.0955531191690000 |
| Ru | 0.5520234565919999 | 0.6615218340179999 | 0.0955531191690000 |
| Ru | 0.8853567899249999 | 0.6615218340179999 | 0.0955531191690000 |
| Ru | 0.2186901232589999 | 0.8615218340179998 | 0.0955531191690000 |
| Ru | 0.5520234565919999 | 0.8615218340179998 | 0.0955531191690000 |
| Ru | 0.8853567899249999 | 0.8615218340179998 | 0.0955531191690000 |
| Ru | 0.0648350526890000 | 0.1155063811580000 | 0.1115066246180000 |
| Ru | 0.3981683860229999 | 0.1155063811580000 | 0.1115066246180000 |
| Ru | 0.7315017193559998 | 0.1155063811580000 | 0.1115066246180000 |
| Ru | 0.0648350526890000 | 0.3155063811580000 | 0.1115066246180000 |
| Ru | 0.3981683860229999 | 0.3155063811580000 | 0.1115066246180000 |
| Ru | 0.7315017193559997 | 0.3155063811580000 | 0.1115066246180000 |
| Ru | 0.0648350526890000 | 0.5155063811579999 | 0.1115066246180000 |
| Ru | 0.3981683860229999 | 0.5155063811579999 | 0.1115066246180000 |
| Ru | 0.7315017193559997 | 0.5155063811579999 | 0.1115066246180000 |
| Ru | 0.0648350526890000 | 0.7155063811579999 | 0.1115066246180000 |
| Ru | 0.3981683860229999 | 0.7155063811579999 | 0.1115066246180000 |
| Ru | 0.7315017193559997 | 0.7155063811579999 | 0.1115066246180000 |

| | | | |
|---|---|---|---|
| Ru | 0.0648350526890000 | 0.9155063811579998 | 0.1115066246180000 |
| Ru | 0.3981683860229999 | 0.9155063811579998 | 0.1115066246180000 |
| Ru | 0.7315017193559997 | 0.9155063811579998 | 0.1115066246180000 |
| Ru | 0.9630914751089997 | 0.5845889784419999 | 0.1918129469190000 |
| Ru | 0.9633322800709998 | 0.7848265802049999 | 0.1921888467229999 |
| Ru | 0.9630998845699998 | 0.1847925152630000 | 0.1926411118799999 |
| Ru | 0.9634638788629999 | 0.3852260734310000 | 0.1925938551069999 |
| Ru | 0.6303255673879999 | 0.3849607896550000 | 0.1924191744390000 |
| Ru | 0.9639465498029998 | 0.9849105143959998 | 0.1927218652719999 |
| Ru | 0.6303935749119998 | 0.1859241388050000 | 0.1927931327350000 |
| Ru | 0.6308560565859998 | 0.5847251676710000 | 0.1928463572159999 |
| Ru | 0.2965804649099999 | 0.5850891664329999 | 0.1929527068090000 |
| Ru | 0.2968231439510000 | 0.7846764645069999 | 0.1929246434640000 |
| Ru | 0.2969718985479999 | 0.9847936336779999 | 0.1929526117990000 |
| Ru | 0.6306348885119999 | 0.7848480562239999 | 0.1931306314300000 |
| Ru | 0.6302586477520000 | 0.9847128348439999 | 0.1930215219950000 |
| Ru | 0.2966603448430000 | 0.1846718323040000 | 0.1936076847260000 |
| Ru | 0.2962486670119999 | 0.3851608712060000 | 0.1936912134930000 |
| Ru | 0.1430387631510000 | 0.8384221897059999 | 0.2079220606889999 |
| Ru | 0.4769374959259999 | 0.0380985402230000 | 0.2080724200630000 |
| Ru | 0.8103007205089999 | 0.0380461255930000 | 0.2081103269990000 |
| Ru | 0.1435473562420000 | 0.2386814388450000 | 0.2079671229939999 |
| Ru | 0.4769446660239999 | 0.8381803728069999 | 0.2081717137510000 |
| Ru | 0.8101468544789998 | 0.2380852026010000 | 0.2081775787319999 |
| Ru | 0.8097814416909999 | 0.4384621698919999 | 0.2083645162080000 |
| Ru | 0.1429559586840000 | 0.6377957906739999 | 0.2083047032680000 |
| Ru | 0.8105308613209999 | 0.6385768155070000 | 0.2081570598820000 |
| Ru | 0.1434215857960000 | 0.4386328759220000 | 0.2084843524789999 |
| Ru | 0.4761897539840000 | 0.6367873777979999 | 0.2085747555610000 |
| Ru | 0.1435804617450000 | 0.0388340109350000 | 0.2085477918649999 |
| Ru | 0.4764590503880000 | 0.2395709643600000 | 0.2085510386049999 |
| Ru | 0.8086856852759999 | 0.8374334214319998 | 0.2088790791010000 |
| Ru | 0.4753383332059999 | 0.4374219572629999 | 0.2090594367580000 |
| Ru | 0.3730834771250000 | 0.1050856317240000 | 0.2848862315979999 |
| Ru | 0.0382736990510000 | 0.3071884849029999 | 0.2853835060550000 |
| Ru | 0.7049870411039998 | 0.3057657561179999 | 0.2856130916509999 |
| Ru | 0.0367641401060000 | 0.5060169632680001 | 0.2854924533170000 |
| Ru | 0.3715011820119999 | 0.7088225785069999 | 0.2856164572600000 |
| Ru | 0.7052875623260000 | 0.1073921206870000 | 0.2856268335230000 |
| Ru | 0.0377788306920000 | 0.7061550751089999 | 0.2854990587890000 |
| Ru | 0.3716800123580000 | 0.9064846429729999 | 0.2857736523850000 |
| Ru | 0.0396589883180000 | 0.9078706512409999 | 0.2858011121249999 |
| Ru | 0.0380484781450000 | 0.1075503982410000 | 0.2858001521390000 |
| Ru | 0.7053500237399999 | 0.9083128353739999 | 0.2861089938990000 |

| | | | |
|---|---|---|---|
| Ru | 0.7043164606259998 | 0.5092217851929999 | 0.2860803095680000 |
| Ru | 0.3690399951769999 | 0.3019143458210000 | 0.2873665482750000 |
| Ru | 0.7056106397219999 | 0.7079716424709999 | 0.2876852622500000 |
| Ru | 0.3704083552219999 | 0.5081461629279999 | 0.2886120310309999 |
| Ru | 0.8890454106989999 | 0.7650461132879999 | 0.2982544155619999 |
| Ru | 0.5523806735159998 | 0.7639781309020000 | 0.2986482482660000 |
| Ru | 0.5544637777509999 | 0.1602415109050000 | 0.2987125239160000 |
| Ru | 0.2167436166280000 | 0.5654819539659999 | 0.2988102209119999 |
| Ru | 0.8877280706119999 | 0.9632244584149999 | 0.2987950253680000 |
| Ru | 0.2194641091480000 | 0.7625170030080000 | 0.2991030048459999 |
| Ru | 0.2202463947220000 | 0.9609616635869999 | 0.2990565819850000 |
| Ru | 0.8867309640489998 | 0.1614599722230000 | 0.2991260140840000 |
| Ru | 0.8861307873929999 | 0.3595433576020000 | 0.2990568395789999 |
| Ru | 0.5508552590349999 | 0.5663785166639999 | 0.2992955292680000 |
| Ru | 0.5536545046910000 | 0.9626580078919999 | 0.2994827948400000 |
| Ru | 0.2167107642530000 | 0.1549284210250000 | 0.2999746400020000 |
| Ru | 0.2142982486030000 | 0.3617221826059999 | 0.3016841145099999 |
| Ru | 0.8887304095879999 | 0.5628104411759999 | 0.3034076021900000 |
| Ru | 0.5530499474249999 | 0.3612449547790000 | 0.3035560225980000 |

Ru: Transition state for $CH_3OCO$ and styrene coupling (ArCH(COOMe)$CH_2$)

($E$ = -1232.2169 eV, $ZPE$ = 4.723855364 eV, $S*T$ = 1.20922192450227 eV)

%BLOCK LATTICE_CART

| | | |
|---|---|---|
| 15.122801570000000 | 0.000000000000000 | 0.000000000000000 |
| -3.641814920000000 | 13.056846300000004 | 0.000000000000000 |
| 0.000000000000000 | 0.000000000000000 | 21.508856710000003 |

Selective dynamics

Direct

| | | | |
|---|---|---|---|
| H | 0.7674982532439999 | 0.1594109702380000 | 0.3897159908860000 |
| H | 0.7701682778489999 | 0.4821863344299999 | 0.4037721012789999 |
| H | 0.4204144690720000 | 0.6143471147760000 | 0.4040817334119999 |
| H | 0.8558992661419999 | 0.2547668824720000 | 0.4285492236169999 |
| H | 0.2873762704640000 | 0.4658747205629999 | 0.4073111996109999 |
| H | 0.7007233807460001 | 0.5704575559100000 | 0.4066701151959999 |
| H | 0.4593148854649999 | 0.2634029184610000 | 0.4104763374549999 |
| H | 0.3068064923659999 | 0.2913606575139999 | 0.4107794111999999 |
| H | 0.5714983822229999 | 0.5864631249519999 | 0.4230379039869999 |
| H | 0.6302732056050000 | 0.3673513405439999 | 0.4479470990589999 |
| H | 0.7428606198819999 | 0.2110122131980000 | 0.4626190831859998 |
| C | 0.6405554691649998 | 0.2807884157139999 | 0.3700877791769999 |
| C | 0.3502537916049999 | 0.4543290141479999 | 0.3869720975920000 |
| C | 0.7056422338179998 | 0.4965016626209999 | 0.3883805562209999 |
| C | 0.3612356409499999 | 0.3497657809190000 | 0.3879779100499999 |
| C | 0.4303527396910000 | 0.5408073985509999 | 0.3900353801109999 |
| C | 0.4529850366620000 | 0.3364380636820000 | 0.3926375725299999 |
| C | 0.5326059767379999 | 0.4247431252719999 | 0.3958166121610000 |
| C | 0.5218399673569999 | 0.5285467869310000 | 0.3963604921319999 |
| C | 0.6250847316209999 | 0.4097958806299999 | 0.4051352448849999 |
| C | 0.7817144476549999 | 0.2299888219450000 | 0.4190209593719999 |
| O | 0.7583785314289999 | 0.3126408470390000 | 0.3877257532809999 |
| O | 0.5970120500409999 | 0.2015789598500000 | 0.3987486005829999 |
| Ru | 0.1407055901600000 | 0.1380862835040000 | 0.0000000000000000 |
| Ru | 0.4740389234929999 | 0.1380862835040000 | 0.0000000000000000 |
| Ru | 0.8073722568239999 | 0.1380862835040000 | 0.0000000000000000 |
| Ru | 0.1407055901580000 | 0.3380862835039999 | 0.0000000000000000 |
| Ru | 0.4740389234929999 | 0.3380862835039999 | 0.0000000000000000 |
| Ru | 0.8073722568250009 | 0.3380862835039999 | 0.0000000000000000 |
| Ru | 0.1407055901600000 | 0.5380862835049999 | 0.0000000000000000 |
| Ru | 0.4740389234929998 | 0.5380862835049999 | 0.0000000000000000 |
| Ru | 0.8073722568279997 | 0.5380862835049999 | 0.0000000000000000 |
| Ru | 0.1407055901600000 | 0.7380862835039999 | 0.0000000000000000 |
| Ru | 0.4740389234929999 | 0.7380862835039999 | 0.0000000000000000 |
| Ru | 0.8073722568259999 | 0.7380862835039999 | 0.0000000000000000 |
| Ru | 0.1407055901600000 | 0.9380862835039999 | 0.0000000000000000 |

| | | | |
|---|---|---|---|
| Ru | 0.4740389234929999 | 0.9380862835039999 | 0.0000000000000000 |
| Ru | 0.8073722568259999 | 0.9380862835039999 | 0.0000000000000000 |
| Ru | 0.3201838529239999 | 0.1920708306440000 | 0.0159535054490000 |
| Ru | 0.6535171862569997 | 0.1920708306440000 | 0.0159535054490000 |
| Ru | 0.9868505195869998 | 0.1920708306440000 | 0.0159535054490000 |
| Ru | 0.3201838529239999 | 0.3920708306440000 | 0.0159535054490000 |
| Ru | 0.6535171862569997 | 0.3920708306440000 | 0.0159535054490000 |
| Ru | 0.9868505195889999 | 0.3920708306440000 | 0.0159535054490000 |
| Ru | 0.3201838529239999 | 0.5920708306439999 | 0.0159535054490000 |
| Ru | 0.6535171862569997 | 0.5920708306439999 | 0.0159535054490000 |
| Ru | 0.9868505195899999 | 0.5920708306439999 | 0.0159535054490000 |
| Ru | 0.3201838529239999 | 0.7920708306449998 | 0.0159535054490000 |
| Ru | 0.6535171862569997 | 0.7920708306439999 | 0.0159535054490000 |
| Ru | 0.9868505195919998 | 0.7920708306419999 | 0.0159535054490000 |
| Ru | 0.3201838529239999 | 0.9920708306439999 | 0.0159535054490000 |
| Ru | 0.6535171862569997 | 0.9920708306439999 | 0.0159535054490000 |
| Ru | 0.9868505195889999 | 0.9920708306439999 | 0.0159535054490000 |
| Ru | 0.2186901232590000 | 0.0615218340179999 | 0.0955531191690000 |
| Ru | 0.5520234565919998 | 0.0615218340179999 | 0.0955531191690000 |
| Ru | 0.8853567899249998 | 0.0615218340179999 | 0.0955531191690000 |
| Ru | 0.2186901232590000 | 0.2615218340179999 | 0.0955531191690000 |
| Ru | 0.5520234565919999 | 0.2615218340179999 | 0.0955531191690000 |
| Ru | 0.8853567899249998 | 0.2615218340179999 | 0.0955531191690000 |
| Ru | 0.2186901232590000 | 0.4615218340180000 | 0.0955531191690000 |
| Ru | 0.5520234565919999 | 0.4615218340180000 | 0.0955531191690000 |
| Ru | 0.8853567899249998 | 0.4615218340180000 | 0.0955531191690000 |
| Ru | 0.2186901232590000 | 0.6615218340169999 | 0.0955531191690000 |
| Ru | 0.5520234565919999 | 0.6615218340169999 | 0.0955531191690000 |
| Ru | 0.8853567899240009 | 0.6615218340169999 | 0.0955531191690000 |
| Ru | 0.2186901232599999 | 0.8615218340179998 | 0.0955531191690000 |
| Ru | 0.5520234565919999 | 0.8615218340179998 | 0.0955531191690000 |
| Ru | 0.8853567899249999 | 0.8615218340179998 | 0.0955531191690000 |
| Ru | 0.0648350526890003 | 0.1155063811580000 | 0.1115066246180000 |
| Ru | 0.3981683860229999 | 0.1155063811580000 | 0.1115066246180000 |
| Ru | 0.7315017193540009 | 0.1155063811580000 | 0.1115066246180000 |
| Ru | 0.0648350526890000 | 0.3155063811580000 | 0.1115066246180000 |
| Ru | 0.3981683860229999 | 0.3155063811580000 | 0.1115066246180000 |
| Ru | 0.7315017193549998 | 0.3155063811580000 | 0.1115066246180000 |
| Ru | 0.0648350526890000 | 0.5155063811589999 | 0.1115066246180000 |
| Ru | 0.3981683860229999 | 0.5155063811589999 | 0.1115066246180000 |
| Ru | 0.7315017193559997 | 0.5155063811589999 | 0.1115066246180000 |
| Ru | 0.0648350526890000 | 0.7155063811579999 | 0.1115066246180000 |
| Ru | 0.3981683860229999 | 0.7155063811579999 | 0.1115066246180000 |
| Ru | 0.7315017193559997 | 0.7155063811579999 | 0.1115066246180000 |

| | | | |
|---|---|---|---|
| Ru | 0.0648350526890000 | 0.9155063811579998 | 0.1115066246180000 |
| Ru | 0.3981683860229999 | 0.9155063811579998 | 0.1115066246180000 |
| Ru | 0.7315017193559997 | 0.9155063811579998 | 0.1115066246180000 |
| Ru | 0.6306717783939999 | 0.5840246117299999 | 0.1917546969270000 |
| Ru | 0.9626506622139988 | 0.3847103608310000 | 0.1924115238030000 |
| Ru | 0.6310329452019998 | 0.3853622020979999 | 0.1933404874749999 |
| Ru | 0.6308194823189999 | 0.7844848189509998 | 0.1926012244580000 |
| Ru | 0.9636587571209998 | 0.7849933753809999 | 0.1926423539100000 |
| Ru | 0.6299660691679998 | 0.9845462998989999 | 0.1924608833000000 |
| Ru | 0.9637745929619999 | 0.5851846075949999 | 0.1927770299050000 |
| Ru | 0.9638639603469998 | 0.1849224932690000 | 0.1927180500440000 |
| Ru | 0.9639442848829999 | 0.9848517717449998 | 0.1928473952950000 |
| Ru | 0.2968020300599999 | 0.5850268800819999 | 0.1931041596840000 |
| Ru | 0.2967121448680000 | 0.7850078332839999 | 0.1931245634490000 |
| Ru | 0.2967345444439999 | 0.9850558970689999 | 0.1930940013670000 |
| Ru | 0.6304341470759998 | 0.1842617330270000 | 0.1930572225330000 |
| Ru | 0.2975863865669999 | 0.1858034957449999 | 0.1932342148329999 |
| Ru | 0.2976359273640000 | 0.3850149555540000 | 0.1937831071199999 |
| Ru | 0.8108442832509998 | 0.2376283873970000 | 0.2072574110030000 |
| Ru | 0.1429986802459999 | 0.8386960416349999 | 0.2079779112580000 |
| Ru | 0.4764768615039999 | 0.8384384787649999 | 0.2077790311319999 |
| Ru | 0.8103505873709997 | 0.8385154747969998 | 0.2082284168290000 |
| Ru | 0.8104385745959998 | 0.0373422278110001 | 0.2078430807500000 |
| Ru | 0.1438388349180000 | 0.0398460529419997 | 0.2084568778710000 |
| Ru | 0.1431848808060000 | 0.6380300075549998 | 0.2081091300400000 |
| Ru | 0.1437445135670000 | 0.4379436260530000 | 0.2083857482020000 |
| Ru | 0.8088920124109998 | 0.4389587518220000 | 0.2084641573200000 |
| Ru | 0.4770712927729999 | 0.0389430312910000 | 0.2086462221680000 |
| Ru | 0.4760453769169999 | 0.6371918141819999 | 0.2084470432680000 |
| Ru | 0.1438322038950000 | 0.2393838935270000 | 0.2088184176719999 |
| Ru | 0.4771479355779999 | 0.2390105332100000 | 0.2093840022119999 |
| Ru | 0.8086921919669999 | 0.6369727047049999 | 0.2086873296610000 |
| Ru | 0.4776563407939999 | 0.4386973951790000 | 0.2098331523929999 |
| Ru | 0.3715957688529999 | 0.7091847086199999 | 0.2852014633919999 |
| Ru | 0.7055486238609998 | 0.1034122823850000 | 0.2839301887590000 |
| Ru | 0.7043882770080009 | 0.3026770104729999 | 0.2853844053430000 |
| Ru | 0.3707056327789999 | 0.1059983739860000 | 0.2857244195060000 |
| Ru | 0.0370635585279999 | 0.3065897573099999 | 0.2855296060529999 |
| Ru | 0.7058034815459999 | 0.9061552255719999 | 0.2855685009560000 |
| Ru | 0.0386192746400000 | 0.9074173564019998 | 0.2858099919870000 |
| Ru | 0.0391359949240002 | 0.7080250097180000 | 0.2858090464129999 |
| Ru | 0.0384096762170001 | 0.5067984018579999 | 0.2857146115549999 |
| Ru | 0.3711147040599999 | 0.9074231250709998 | 0.2858041946939999 |
| Ru | 0.0383509666530000 | 0.1073322252510000 | 0.2861451827159999 |

| | | | |
|---|---|---|---|
| Ru | 0.7058288481669999 | 0.7087502887540000 | 0.2858922551820000 |
| Ru | 0.7077877633410000 | 0.5100449991180001 | 0.2882448932680000 |
| Ru | 0.3717650966229999 | 0.3078875712069999 | 0.2890388424270000 |
| Ru | 0.3689461458300000 | 0.5073239362629999 | 0.2886717058300000 |
| Ru | 0.8880240454889998 | 0.3624541255059999 | 0.2992388089009999 |
| Ru | 0.5545480602499999 | 0.7638110043039998 | 0.2981310709029999 |
| Ru | 0.8886382633799999 | 0.5628711163639999 | 0.2986212737969999 |
| Ru | 0.5534207367199999 | 0.9589291894249999 | 0.2986869905149999 |
| Ru | 0.2169937771000000 | 0.5656098259839999 | 0.2990005742649999 |
| Ru | 0.2182307485470000 | 0.1584995855660000 | 0.2990477300159999 |
| Ru | 0.8869698224499998 | 0.1613169362580000 | 0.2985417828970000 |
| Ru | 0.8872474605679998 | 0.7621517274170000 | 0.2989982628310000 |
| Ru | 0.2198145751610000 | 0.7629577008989998 | 0.2993599357849999 |
| Ru | 0.2198437992750000 | 0.9614532387089998 | 0.2993485515879999 |
| Ru | 0.2148152731540000 | 0.3600963660009999 | 0.2996041542960000 |
| Ru | 0.8872916949020010 | 0.9616075576729999 | 0.2993361504209999 |
| Ru | 0.5507906736019999 | 0.5652853469689999 | 0.3002188003569999 |
| Ru | 0.5503060460759998 | 0.1534212872510000 | 0.3009474161160000 |
| Ru | 0.5553681210099999 | 0.3612030094059999 | 0.3073344234350000 |

Ru: Final state for $CH_3OCO$ and styrene coupling (ArCH(COOMe)$CH_2$)

($E$ = -1233.1157 eV, $ZPE$ = 4.837496119 eV, $S*T$ = 1.212619089 eV)

%BLOCK LATTICE_CART

| | | |
|---|---|---|
| 15.122801570000000 | 0.000000000000000 | 0.000000000000000 |
| -3.641814920000000 | 13.056846300000004 | 0.000000000000000 |
| 0.000000000000000 | 0.000000000000000 | 21.508856710000003 |

Selective dynamics

Direct

| | | | |
|---|---|---|---|
| H | 0.7642111501679998 | 0.1526705153350000 | 0.3928854871540000 |
| H | 0.7884317254579999 | 0.4847883960470000 | 0.3987092787670000 |
| H | 0.4410738192719999 | 0.6046189678269999 | 0.4065400991429999 |
| H | 0.4716122900409999 | 0.2506629967810000 | 0.4049948840579999 |
| H | 0.8542655704909998 | 0.2387913437610000 | 0.4362507172189999 |
| H | 0.3051121848930000 | 0.4594174994449999 | 0.4093616700520000 |
| H | 0.7131667818599999 | 0.5635398255509999 | 0.4079647392210000 |
| H | 0.3219449043879999 | 0.2841803700339999 | 0.4108916295969999 |
| H | 0.5894318850359999 | 0.5726058378459999 | 0.4214981631870000 |
| H | 0.7403776206829997 | 0.1929644914960000 | 0.4693593908849999 |
| H | 0.6512186953279999 | 0.3915071924819999 | 0.4618341506409999 |
| C | 0.6674027865769999 | 0.3003024181730000 | 0.3828946789809999 |
| C | 0.3663312177529999 | 0.4469081056809999 | 0.3872107552860000 |
| C | 0.7196911374299999 | 0.4923487609230000 | 0.3866543380469999 |
| C | 0.3763991824839999 | 0.3423618591700000 | 0.3879569077529999 |
| C | 0.4486605779469999 | 0.5310991807780000 | 0.3904979654560000 |
| C | 0.4677827179899999 | 0.3265113651770000 | 0.3910142339409999 |
| C | 0.5504833446039998 | 0.4099324629200000 | 0.3936262574189999 |
| C | 0.5387680539789999 | 0.5143729603659999 | 0.3958029153389999 |
| C | 0.7802879636929999 | 0.2171705850639999 | 0.4267472540779999 |
| C | 0.6458972610719999 | 0.3979346554920000 | 0.4104300190960000 |
| O | 0.7617001191989999 | 0.3072653255100000 | 0.4013975911989999 |
| O | 0.6081114879440001 | 0.2083301045190000 | 0.3913076075060000 |
| Ru | 0.1407055901600000 | 0.1380862835040000 | 0.0000000000000000 |
| Ru | 0.4740389234929999 | 0.1380862835040000 | 0.0000000000000000 |
| Ru | 0.8073722568259998 | 0.1380862835040000 | 0.0000000000000000 |
| Ru | 0.1407055901600000 | 0.3380862835039999 | 0.0000000000000000 |
| Ru | 0.4740389234929999 | 0.3380862835039999 | 0.0000000000000000 |
| Ru | 0.8073722568260010 | 0.3380862835039999 | 0.0000000000000000 |
| Ru | 0.1407055901600000 | 0.5380862835039999 | 0.0000000000000000 |
| Ru | 0.4740389234929999 | 0.5380862835039999 | 0.0000000000000000 |
| Ru | 0.8073722568259999 | 0.5380862835039999 | 0.0000000000000000 |
| Ru | 0.1407055901600000 | 0.7380862835039999 | 0.0000000000000000 |
| Ru | 0.4740389234929999 | 0.7380862835039999 | 0.0000000000000000 |
| Ru | 0.8073722568259999 | 0.7380862835039999 | 0.0000000000000000 |
| Ru | 0.1407055901600000 | 0.9380862835039999 | 0.0000000000000000 |

| | | | |
|---|---|---|---|
| Ru | 0.4740389234929999 | 0.9380862835039999 | 0.0000000000000000 |
| Ru | 0.8073722568259999 | 0.9380862835039999 | 0.0000000000000000 |
| Ru | 0.3201838529239999 | 0.1920708306440000 | 0.0159535054490000 |
| Ru | 0.6535171862569997 | 0.1920708306440000 | 0.0159535054490000 |
| Ru | 0.9868505195899998 | 0.1920708306440000 | 0.0159535054490000 |
| Ru | 0.3201838529239999 | 0.3920708306440000 | 0.0159535054490000 |
| Ru | 0.6535171862569997 | 0.3920708306440000 | 0.0159535054490000 |
| Ru | 0.9868505195899998 | 0.3920708306440000 | 0.0159535054490000 |
| Ru | 0.3201838529239999 | 0.5920708306439999 | 0.0159535054490000 |
| Ru | 0.6535171862569997 | 0.5920708306439999 | 0.0159535054490000 |
| Ru | 0.9868505195899999 | 0.5920708306439999 | 0.0159535054490000 |
| Ru | 0.3201838529239999 | 0.7920708306439999 | 0.0159535054490000 |
| Ru | 0.6535171862569997 | 0.7920708306439999 | 0.0159535054490000 |
| Ru | 0.9868505195899999 | 0.7920708306439999 | 0.0159535054490000 |
| Ru | 0.3201838529239999 | 0.9920708306439999 | 0.0159535054490000 |
| Ru | 0.6535171862569997 | 0.9920708306439999 | 0.0159535054490000 |
| Ru | 0.9868505195899999 | 0.9920708306439999 | 0.0159535054490000 |
| Ru | 0.2186901232590000 | 0.0615218340179999 | 0.0955531191690000 |
| Ru | 0.5520234565919998 | 0.0615218340179999 | 0.0955531191690000 |
| Ru | 0.8853567899249998 | 0.0615218340179999 | 0.0955531191690000 |
| Ru | 0.2186901232590000 | 0.2615218340179999 | 0.0955531191690000 |
| Ru | 0.5520234565919999 | 0.2615218340179999 | 0.0955531191690000 |
| Ru | 0.8853567899249998 | 0.2615218340179999 | 0.0955531191690000 |
| Ru | 0.2186901232590000 | 0.4615218340180000 | 0.0955531191690000 |
| Ru | 0.5520234565919999 | 0.4615218340180000 | 0.0955531191690000 |
| Ru | 0.8853567899249998 | 0.4615218340180000 | 0.0955531191690000 |
| Ru | 0.2186901232590000 | 0.6615218340179999 | 0.0955531191690000 |
| Ru | 0.5520234565919999 | 0.6615218340179999 | 0.0955531191690000 |
| Ru | 0.8853567899249999 | 0.6615218340179999 | 0.0955531191690000 |
| Ru | 0.2186901232589999 | 0.8615218340179998 | 0.0955531191690000 |
| Ru | 0.5520234565919999 | 0.8615218340179998 | 0.0955531191690000 |
| Ru | 0.8853567899249999 | 0.8615218340179998 | 0.0955531191690000 |
| Ru | 0.0648350526890003 | 0.1155063811580000 | 0.1115066246180000 |
| Ru | 0.3981683860229999 | 0.1155063811580000 | 0.1115066246180000 |
| Ru | 0.7315017193559998 | 0.1155063811580000 | 0.1115066246180000 |
| Ru | 0.0648350526890000 | 0.3155063811580000 | 0.1115066246180000 |
| Ru | 0.3981683860229999 | 0.3155063811580000 | 0.1115066246180000 |
| Ru | 0.7315017193559997 | 0.3155063811580000 | 0.1115066246180000 |
| Ru | 0.0648350526890000 | 0.5155063811579999 | 0.1115066246180000 |
| Ru | 0.3981683860229999 | 0.5155063811579999 | 0.1115066246180000 |
| Ru | 0.7315017193559997 | 0.5155063811579999 | 0.1115066246180000 |
| Ru | 0.0648350526890000 | 0.7155063811579999 | 0.1115066246180000 |
| Ru | 0.3981683860229999 | 0.7155063811579999 | 0.1115066246180000 |
| Ru | 0.7315017193559997 | 0.7155063811579999 | 0.1115066246180000 |

| | | | |
|---|---|---|---|
| Ru | 0.0648350526890000 | 0.9155063811579998 | 0.1115066246180000 |
| Ru | 0.3981683860229999 | 0.9155063811579998 | 0.1115066246180000 |
| Ru | 0.7315017193559997 | 0.9155063811579998 | 0.1115066246180000 |
| Ru | 0.6305296428339999 | 0.5860802886089999 | 0.1916543026039999 |
| Ru | 0.6307293947479999 | 0.3860504162210000 | 0.1916151727809999 |
| Ru | 0.9626414673119998 | 0.3850544378490000 | 0.1927487495209999 |
| Ru | 0.9636045703569998 | 0.1852512512480000 | 0.1926965429310000 |
| Ru | 0.9635928919569998 | 0.7852078411399999 | 0.1926987534730000 |
| Ru | 0.9636036823200009 | 0.5853790765619998 | 0.1929174986780000 |
| Ru | 0.6306100216009999 | 0.7848934754949999 | 0.1927460794099999 |
| Ru | 0.2965566102209999 | 0.1848769427040000 | 0.1927171689630000 |
| Ru | 0.9633884338269998 | 0.9848899962179998 | 0.1927896893590000 |
| Ru | 0.6300134310269989 | 0.9853720291309999 | 0.1927168608450000 |
| Ru | 0.2968537831120000 | 0.5849123883109999 | 0.1931245940740000 |
| Ru | 0.2968019717100000 | 0.7849674746599999 | 0.1930474393319999 |
| Ru | 0.2967608907009999 | 0.9851038114999998 | 0.1930667271879999 |
| Ru | 0.6299034146819998 | 0.1848730072940000 | 0.1939366509670000 |
| Ru | 0.2970740887350000 | 0.3852287612399999 | 0.1939732224260000 |
| Ru | 0.8095270200879999 | 0.2387092395220000 | 0.2080152043019999 |
| Ru | 0.1430427840660000 | 0.8386469904499998 | 0.2079134136570000 |
| Ru | 0.4769452915419999 | 0.4397141564699999 | 0.2082276642160000 |
| Ru | 0.8099805158359998 | 0.8386363836629999 | 0.2082405100399999 |
| Ru | 0.4766249290089999 | 0.8388648547029999 | 0.2082126476589999 |
| Ru | 0.1434371935760000 | 0.0393799771009999 | 0.2082789113279999 |
| Ru | 0.8099446117259999 | 0.0383821917000000 | 0.2081519593639999 |
| Ru | 0.4757626952569999 | 0.2381925401580000 | 0.2083842604690000 |
| Ru | 0.1435152378320000 | 0.4381794324789999 | 0.2083616814900000 |
| Ru | 0.1430748394420000 | 0.6377818803939999 | 0.2084545193769999 |
| Ru | 0.4756419270489999 | 0.6381638557629999 | 0.2081944112309999 |
| Ru | 0.4774607704449999 | 0.0396486955440001 | 0.2087690543080000 |
| Ru | 0.1436975059610000 | 0.2390721437760000 | 0.2086391411480000 |
| Ru | 0.8078091973849999 | 0.4384699374959999 | 0.2086370196500000 |
| Ru | 0.8084408346640000 | 0.6375454380469999 | 0.2089274395809999 |
| Ru | 0.0376185521270001 | 0.3067911432129999 | 0.2853734475479999 |
| Ru | 0.3716536922829999 | 0.7087273515460000 | 0.2856876382119999 |
| Ru | 0.3716217288190000 | 0.1052051926120000 | 0.2854995746469999 |
| Ru | 0.0388198453720001 | 0.9078506290019998 | 0.2857992129699999 |
| Ru | 0.7061074870389998 | 0.9074957202839998 | 0.2856636905350000 |
| Ru | 0.7045872558929999 | 0.1051541382070000 | 0.2853756416420000 |
| Ru | 0.0391554773549998 | 0.7080167358019999 | 0.2858585996899999 |
| Ru | 0.3717998621990000 | 0.9081241031549998 | 0.2858112690719999 |
| Ru | 0.0381542529460002 | 0.1074039479850000 | 0.2858729704910000 |
| Ru | 0.0385796855879998 | 0.5070844232979999 | 0.2859732954570000 |
| Ru | 0.7033220765820007 | 0.3098848639899999 | 0.2859436756429999 |

| | | | |
|---|---|---|---|
| Ru | 0.7058733867789998 | 0.7093248966039999 | 0.2862915636969999 |
| Ru | 0.3712432859939999 | 0.3065604639100000 | 0.2878654876899999 |
| Ru | 0.7086059787189999 | 0.5091326356949999 | 0.2877675535289999 |
| Ru | 0.3706214618199999 | 0.5063274561569999 | 0.2891890204659999 |
| Ru | 0.8871937415499998 | 0.3617712774299999 | 0.2991722854609999 |
| Ru | 0.5542417560959999 | 0.7645177973129999 | 0.2984271072540000 |
| Ru | 0.5536086133739999 | 0.9601542507119999 | 0.2989667236409999 |
| Ru | 0.2180344991440000 | 0.1580427703360000 | 0.2991972768589999 |
| Ru | 0.8866982048219999 | 0.1618682829930000 | 0.2983718143469999 |
| Ru | 0.8885144273849998 | 0.5622199406899999 | 0.2991643228699999 |
| Ru | 0.2183225105100000 | 0.5644687630209999 | 0.2992066243839999 |
| Ru | 0.8872659033819997 | 0.7626805653319999 | 0.2990975787280000 |
| Ru | 0.2202387714169999 | 0.7629786447789999 | 0.2991909648900000 |
| Ru | 0.2199194050829999 | 0.9610742156199997 | 0.2995224554209999 |
| Ru | 0.8871637553399998 | 0.9616196970399998 | 0.2994100319090000 |
| Ru | 0.2148256372030000 | 0.3598269837790000 | 0.2997377392719999 |
| Ru | 0.5495467777599998 | 0.3655518402859999 | 0.2972859994590000 |
| Ru | 0.5529461530389999 | 0.5655083189469999 | 0.3004718384460000 |
| Ru | 0.5519373803959999 | 0.1613382666690000 | 0.3038606979330000 |

Ru: Initial state for $ArCHCH_2(COOMe)$ and H coupling
(*E* =-1237.4959 eV, *ZPE* = 4.9937064535 eV, *S**T* = 1.33609087640458 eV)
AutoCreatByScript: H C O Ru
1.00000000000000
15.1228015700000000 0.0000000000000000 0.0000000000
-3.6418149199999998 13.0568463000000001 0.0000000000
0.0000000000000000 0.0000000000000000 21.5088567099
H C O Ru
12 10 2 120
Selective dynamics
Direct
0.6666138265156273 0.1951151045249037 0.3429124964540304
0.8674100082840033 0.7126795561022290 0.3920684668034318
0.4920844822971226 0.5607785913970814 0.3997483654015048
0.4265048176836859 0.2298154590889580 0.4016690107701538
0.2632277178532448 0.2203145425729222 0.4027263080670028
0.3317815831438304 0.5503449460564598 0.4093529930037179
0.8017536807472015 0.8005812818898945 0.4177844634850186
0.2185721029920077 0.3814440807209499 0.4220764173095368
0.5772912910587640 0.3243974232160673 0.4230107932014514
0.6044824315259725 0.5457085629323349 0.4461103783377282
0.8100140283596654 0.6921514283749175 0.4648274648473900
0.6777751002847776 0.4689472178213874 0.4662197254065907
0.7116213920024690 0.5508579923494671 0.3809504879605313
0.4453868662603959 0.4893014621857896 0.3815504727635518
0.3103857711340058 0.2934453876503130 0.3875918812874669
0.4063053957003127 0.2981293168559744 0.3879125745749268
0.3483979478414682 0.4841153800320493 0.3883968276626970
0.4767512562360353 0.3954029856043291 0.3885131292139260
0.2783654768033740 0.3854240703547488 0.3924252674620434
0.5704324477358310 0.3960078000135012 0.4027365995394079
0.8047592457621701 0.7208428714452465 0.4175148962587667
0.6398543335242431 0.4912469919713737 0.4272520012606923
0.7904384193375502 0.5248740212350794 0.3784798535792607
0.7216125934694804 0.6629249564421957 0.3879329381601258
0.1407055901600032 0.1380862835040010 0.0000000000000000
0.4740389234929978 0.1380862835040010 0.0000000000000000
0.8073722568259991 0.1380862835040010 0.0000000000000000
0.1407055901600029 0.3380862835039946 0.0000000000000000
0.4740389234929973 0.3380862835039946 0.0000000000000000
0.8073722568259990 0.3380862835039946 0.0000000000000000
0.1407055901600027 0.5380862835039967 0.0000000000000000
0.4740389234929969 0.5380862835039967 0.0000000000000000
0.8073722568259998 0.5380862835039967 0.0000000000000000

| | | |
|---|---|---|
| 0.1407055901600026 | 0.7380862835039964 | 0.0000000000000000 |
| 0.4740389234929977 | 0.7380862835039964 | 0.0000000000000000 |
| 0.8073722568259982 | 0.7380862835039964 | 0.0000000000000000 |
| 0.1407055901600026 | 0.9380862835039901 | 0.0000000000000000 |
| 0.4740389234929977 | 0.9380862835039901 | 0.0000000000000000 |
| 0.8073722568259990 | 0.9380862835039901 | 0.0000000000000000 |
| 0.3201838529240041 | 0.1920708306439974 | 0.0159535054489979 |
| 0.6535171862569986 | 0.1920708306439974 | 0.0159535054489979 |
| 0.9868505195900004 | 0.1920708306439974 | 0.0159535054489979 |
| 0.3201838529240035 | 0.3920708306439984 | 0.0159535054489979 |
| 0.6535171862569973 | 0.3920708306439984 | 0.0159535054489979 |
| 0.9868505195900017 | 0.3920708306439984 | 0.0159535054489979 |
| 0.3201838529240035 | 0.5920708306439935 | 0.0159535054489979 |
| 0.6535171862569977 | 0.5920708306439935 | 0.0159535054489979 |
| 0.9868505195899993 | 0.5920708306439935 | 0.0159535054489979 |
| 0.3201838529240035 | 0.7920708306439946 | 0.0159535054489979 |
| 0.6535171862569981 | 0.7920708306439946 | 0.0159535054489979 |
| 0.9868505195900001 | 0.7920708306439946 | 0.0159535054489979 |
| 0.3201838529240032 | 0.9920708306439946 | 0.0159535054489979 |
| 0.6535171862569977 | 0.9920708306439946 | 0.0159535054489979 |
| 0.9868505195900000 | 0.9920708306439946 | 0.0159535054489979 |
| 0.2186901232590020 | 0.0615218340180016 | 0.0955531191689971 |
| 0.5520234565920040 | 0.0615218340180016 | 0.0955531191689971 |
| 0.8853567899249992 | 0.0615218340180016 | 0.0955531191689971 |
| 0.2186901232590018 | 0.2615218340179959 | 0.0955531191689971 |
| 0.5520234565920045 | 0.2615218340179959 | 0.0955531191689971 |
| 0.8853567899249983 | 0.2615218340179959 | 0.0955531191689971 |
| 0.2186901232590017 | 0.4615218340179966 | 0.0955531191689971 |
| 0.5520234565920039 | 0.4615218340179966 | 0.0955531191689971 |
| 0.8853567899250000 | 0.4615218340179966 | 0.0955531191689971 |
| 0.2186901232590010 | 0.6615218340179970 | 0.0955531191689971 |
| 0.5520234565920039 | 0.6615218340179970 | 0.0955531191689971 |
| 0.8853567899250003 | 0.6615218340179970 | 0.0955531191689971 |
| 0.2186901232590009 | 0.8615218340179911 | 0.0955531191689971 |
| 0.5520234565920039 | 0.8615218340179911 | 0.0955531191689971 |
| 0.8853567899249986 | 0.8615218340179911 | 0.0955531191689971 |
| 0.0648350526889985 | 0.1155063811579982 | 0.1115066246180016 |
| 0.3981683860230019 | 0.1155063811579982 | 0.1115066246180016 |
| 0.7315017193559981 | 0.1155063811579982 | 0.1115066246180016 |
| 0.0648350526889982 | 0.3155063811579995 | 0.1115066246180016 |
| 0.3981683860230019 | 0.3155063811579995 | 0.1115066246180016 |
| 0.7315017193559979 | 0.3155063811579995 | 0.1115066246180016 |
| 0.0648350526889982 | 0.5155063811579940 | 0.1115066246180016 |
| 0.3981683860230019 | 0.5155063811579940 | 0.1115066246180016 |

| | | |
|---|---|---|
| 0.7315017193559971 | 0.5155063811579940 | 0.1115066246180016 |
| 0.0648350526889978 | 0.7155063811579943 | 0.1115066246180016 |
| 0.3981683860230019 | 0.7155063811579943 | 0.1115066246180016 |
| 0.7315017193559968 | 0.7155063811579943 | 0.1115066246180016 |
| 0.0648350526889976 | 0.9155063811579885 | 0.1115066246180016 |
| 0.3981683860230009 | 0.9155063811579885 | 0.1115066246180016 |
| 0.7315017193559959 | 0.9155063811579885 | 0.1115066246180016 |
| 0.9633383026806764 | 0.5849205790910771 | 0.1917083007590078 |
| 0.9634090484160741 | 0.7848114258773894 | 0.1922493918871445 |
| 0.6305504586947970 | 0.3854362567718035 | 0.1920382611536611 |
| 0.9633043971947727 | 0.3851924237580305 | 0.1924391346911017 |
| 0.9630806770554362 | 0.1848626453902284 | 0.1924631482681282 |
| 0.6298062131966504 | 0.1857177126365582 | 0.1932548779174980 |
| 0.9641073212232558 | 0.9848847777385493 | 0.1926820878466662 |
| 0.6308598553706524 | 0.5847027298574416 | 0.1928352962393535 |
| 0.2967526162143280 | 0.5851786608693735 | 0.1928862091314150 |
| 0.2968266803838903 | 0.7846207854504079 | 0.1930086548047384 |
| 0.2966316004086901 | 0.9847119455735602 | 0.1928829776770371 |
| 0.6304245000694744 | 0.9846465325552047 | 0.1926051803009920 |
| 0.6308767118335439 | 0.7847837782799607 | 0.1930276425534856 |
| 0.2963861953940267 | 0.1847298977290862 | 0.1935652139808259 |
| 0.2964232701678706 | 0.3851770475892164 | 0.1935814670307564 |
| 0.1435191939725641 | 0.2390068983526496 | 0.2079741586289805 |
| 0.1430987141905126 | 0.8382949823087481 | 0.2078571818846118 |
| 0.4764644299060048 | 0.0378095960985430 | 0.2076329917336973 |
| 0.8099891238265786 | 0.0380195226688676 | 0.2082009490749239 |
| 0.8098734425072303 | 0.2381275745322968 | 0.2079922536690664 |
| 0.8104215738920962 | 0.6384863484798232 | 0.2082536434449689 |
| 0.4771767078091521 | 0.8384729685520870 | 0.2082053836894718 |
| 0.8096637924403011 | 0.4385572852135657 | 0.2083245192177926 |
| 0.1428931234449093 | 0.6377627363082795 | 0.2083767541344115 |
| 0.1436022242206811 | 0.4386389370508531 | 0.2085242380136746 |
| 0.1434623377043419 | 0.0388394376985347 | 0.2085740835551832 |
| 0.4763551403184009 | 0.2398235655194509 | 0.2086800108773963 |
| 0.4763329500429769 | 0.6369568589715124 | 0.2085418133010977 |
| 0.8090204174994369 | 0.8376135708396546 | 0.2089673035428881 |
| 0.4754653269203905 | 0.4372859534154037 | 0.2091369076180447 |
| 0.3725154757103395 | 0.1049769409768907 | 0.2842633689868945 |
| 0.0386458460873616 | 0.3074786022581669 | 0.2854133551288340 |
| 0.0369561408074045 | 0.5063230560807589 | 0.2853545692292339 |
| 0.0379152583172153 | 0.7062344767903230 | 0.2855711173494573 |
| 0.7059103868276128 | 0.1070478838302824 | 0.2859608762271273 |
| 0.7048686384616892 | 0.3060281294863304 | 0.2859033873158374 |
| 0.3714830848086934 | 0.7088110328488211 | 0.2856671409597541 |

| | | |
|---|---|---|
| 0.3718103509600824 | 0.9070436579620805 | 0.2856763628068029 |
| 0.0380277788345451 | 0.1074698095963405 | 0.2857231004223204 |
| 0.0397465513923916 | 0.9079602950038694 | 0.2858171036581518 |
| 0.7044418890248947 | 0.5093340612560042 | 0.2862993344292155 |
| 0.7053852904440829 | 0.9080986897295252 | 0.2858198315901846 |
| 0.3691142654629102 | 0.3018731095287959 | 0.2873974262581364 |
| 0.7057183582976153 | 0.7078681374362688 | 0.2876959117352518 |
| 0.3704786660828727 | 0.5082243580726973 | 0.2885707675193971 |
| 0.8892864210767391 | 0.7651138961479599 | 0.2983144547070580 |
| 0.5524908031594451 | 0.7635781331161062 | 0.2984504102220449 |
| 0.5522601031553588 | 0.1592641536636517 | 0.2996407248434939 |
| 0.8876836606088845 | 0.9631508321665430 | 0.2987928892761443 |
| 0.2168868266876997 | 0.5656236722112264 | 0.2988682935759010 |
| 0.8869495040784869 | 0.3596094277661892 | 0.2987709177645568 |
| 0.2200292656283134 | 0.9609471934112072 | 0.2992592655194731 |
| 0.8877611246650461 | 0.1622043478939905 | 0.2988235886313283 |
| 0.2196613169082074 | 0.7627244913760406 | 0.2992013104116399 |
| 0.5509904239206560 | 0.5663799230276103 | 0.2992258844814841 |
| 0.5532111600962357 | 0.9607775340461159 | 0.2989262830901594 |
| 0.2164356072207259 | 0.1550919482542644 | 0.3001069633813336 |
| 0.2146327856344689 | 0.3618131897898660 | 0.3016086025242248 |
| 0.8889235026712827 | 0.5630989671236116 | 0.3032368498522860 |
| 0.5527921050890148 | 0.3626390727644229 | 0.3031771182805014 |

Ru: Transition state state for ArCHCH$_2$(COOMe) and H coupling

($E$ = -1237.004 eV, $ZPE$ = 5.0258615655 eV, $S*T$ = 1.28862685461328 eV)

AutoCreatByScript: H C O Ru

1.00000000000000

| | | |
|---|---|---|
| 15.1228015700000000 | 0.0000000000000000 | 0.0000000000000000 |
| -3.6418149199999998 | 13.0568463000000001 | 0.0000000000000000 |
| 0.0000000000000000 | 0.0000000000000000 | 21.5088567099999999 |

| H | C | O | Ru |
|---|---|---|---|
| 12 | 10 | 2 | 120 |

Selective dynamics

Direct

| | | |
|---|---|---|
| 0.6393097619770505 | 0.3455794858785999 | 0.3600149103892133 |
| 0.8578294415668877 | 0.7390253588494737 | 0.4026593080875795 |
| 0.4117029513341333 | 0.6121286651524416 | 0.4040830010171985 |
| 0.2736957242100588 | 0.4695183554524599 | 0.4049608730943285 |
| 0.4348659621339569 | 0.2552625125845871 | 0.4073480206111054 |
| 0.2854895774824384 | 0.2908132254533235 | 0.4083829136999939 |
| 0.7668927476365945 | 0.7932954979057749 | 0.4273882701509037 |
| 0.5601004238813656 | 0.5760847623370380 | 0.4221127531334937 |
| 0.5885420220730255 | 0.3177147634910638 | 0.4372578324692220 |
| 0.6611011890028943 | 0.5163162236146466 | 0.4685800928631159 |
| 0.7944579537209330 | 0.6908032181373694 | 0.4711513507216247 |
| 0.7319345674442059 | 0.4347993085442019 | 0.4520916839117884 |
| 0.7375679581857406 | 0.5453888213868842 | 0.3815958170938739 |
| 0.3359042140141947 | 0.4545762379766207 | 0.3855007787324828 |
| 0.3418284644539963 | 0.3478926019856082 | 0.3862035200560235 |
| 0.4187287354129436 | 0.5374684061072862 | 0.3897168180828467 |
| 0.4315237410376769 | 0.3294936146541553 | 0.3896291393109710 |
| 0.5141378914448027 | 0.4141047184126441 | 0.3952845492744582 |
| 0.5083420224390849 | 0.5198301848194788 | 0.3957861719141526 |
| 0.5996444040400788 | 0.3881795602107480 | 0.4097867816984068 |
| 0.7887290361771335 | 0.7227044301410457 | 0.4247531115747649 |
| 0.6825799753215326 | 0.4716761301651624 | 0.4314038369587564 |
| 0.8281017624296567 | 0.5544880867168194 | 0.3889118810533980 |
| 0.7200058442767923 | 0.6515094342657024 | 0.3884415798314610 |
| 0.1407055901599493 | 0.1380862835038905 | 0.0000000000000000 |
| 0.4740389234927057 | 0.1380862835038905 | 0.0000000000000000 |
| 0.8073722568246217 | 0.1380862835038905 | 0.0000000000000000 |
| 0.1407055901600364 | 0.3380862835038482 | 0.0000000000000000 |
| 0.4740389234928832 | 0.3380862835038482 | 0.0000000000000000 |
| 0.8073722568260202 | 0.3380862835038482 | 0.0000000000000000 |
| 1.1407055901609171 | 0.5380862835045566 | 0.0000000000000000 |
| 0.4740389234930708 | 0.5380862835045566 | 0.0000000000000000 |
| 0.8073722568283758 | 0.5380862835045566 | 0.0000000000000000 |

| | | | |
|---|---|---|---|
| 1.1407055901603187 | 0.7380862835032720 | 0.0000000000000000 | |
| 0.4740389234930861 | 0.7380862835032720 | 0.0000000000000000 | |
| 0.8073722568258841 | 0.7380862835032720 | 0.0000000000000000 | |
| 0.1407055901599792 | -0.0619137164963516 | 0.0000000000000000 | F |
| 0.4740389234930552 | -0.0619137164963516 | 0.0000000000000000 | F |
| 0.8073722568258999 | -0.0619137164963196 | 0.0000000000000000 | F |
| 0.3201838529240049 | 0.1920708306439443 | 0.0159535054489979 | |
| 0.6535171862570304 | 0.1920708306439443 | 0.0159535054489979 | |
| 0.9868505195887651 | 0.1920708306439443 | 0.0159535054489979 | |
| 0.3201838529241401 | 0.3920708306436292 | 0.0159535054489979 | |
| 0.6535171862571127 | 0.3920708306436292 | 0.0159535054489979 | |
| 0.9868505195889512 | 0.3920708306436292 | 0.0159535054489979 | |
| 0.3201838529240035 | 0.5920708306437813 | 0.0159535054489979 | |
| 0.6535171862568361 | 0.5920708306437813 | 0.0159535054489979 | |
| 0.9868505195898771 | 0.5920708306437813 | 0.0159535054489979 | |
| 0.3201838529239317 | -0.2079291693551725 | 0.0159535054489979 | F |
| 0.6535171862567104 | 0.7920708306429167 | 0.0159535054489979 | |
| 0.9868505195921142 | 0.7920708306415856 | 0.0159535054489979 | |
| 0.3201838529239891 | -0.0079291693564165 | 0.0159535054489979 | F |
| 0.6535171862566677 | -0.0079291693564658 | 0.0159535054489979 | F |
| 0.9868505195890367 | -0.0079291693561607 | 0.0159535054489979 | F |
| 0.2186901232590077 | 0.0615218340179612 | 0.0955531191689971 | |
| 0.5520234565916763 | 0.0615218340179612 | 0.0955531191689971 | |
| 0.8853567899250292 | 0.0615218340179612 | 0.0955531191689971 | |
| 0.2186901232588825 | 0.2615218340177750 | 0.0955531191689971 | |
| 0.5520234565919072 | 0.2615218340177750 | 0.0955531191689971 | |
| 0.8853567899247908 | 0.2615218340177750 | 0.0955531191689971 | |
| 0.2186901232590446 | 0.4615218340176939 | 0.0955531191689971 | |
| 0.5520234565919944 | 0.4615218340176939 | 0.0955531191689971 | |
| 0.8853567899249822 | 0.4615218340176939 | 0.0955531191689971 | |
| 0.2186901232600037 | 0.6615218340174791 | 0.0955531191689971 | |
| 0.5520234565921416 | 0.6615218340174791 | 0.0955531191689971 | |
| 0.8853567899252685 | 0.6615218340174791 | 0.0955531191689971 | |
| 0.2186901232599892 | -0.1384781659822680 | 0.0955531191689971 | F |
| 0.5520234565921862 | -0.1384781659820181 | 0.0955531191689971 | F |
| 0.8853567899249095 | 0.8615218340178467 | 0.0955531191689971 | |
| 1.0648350526883459 | 0.1155063811579341 | 0.1115066246180021 | |
| 0.3981683860228895 | 0.1155063811579341 | 0.1115066246180021 | |
| 0.7315017193538185 | 0.1155063811579341 | 0.1115066246180021 | |
| 1.0648350526888104 | 0.3155063811579493 | 0.1115066246180021 | |
| 0.3981683860229905 | 0.3155063811579493 | 0.1115066246180021 | |
| 0.7315017193549292 | 0.3155063811579493 | 0.1115066246180021 | |
| 1.0648350526898436 | 0.5155063811585539 | 0.1115066246180021 | |
| 0.3981683860230105 | 0.5155063811585539 | 0.1115066246180021 | |

| | | | |
|---|---|---|---|
| 0.7315017193558839 | 0.5155063811585539 | 0.1115066246180021 | |
| 1.0648350526902575 | 0.7155063811575281 | 0.1115066246180021 | |
| 0.3981683860229837 | 0.7155063811575281 | 0.1115066246180021 | |
| 0.7315017193556290 | 0.7155063811575281 | 0.1115066246180021 | |
| 1.0648350526889514 | 0.9155063811572408 | 0.1115066246180021 | |
| 0.3981683860227124 | -0.0844936188422264 | 0.1115066246180021 | F |
| 0.7315017193556038 | -0.0844936188422264 | 0.1115066246180021 | F |
| 0.6306960871703050 | 0.5852880216135939 | 0.1921480629324552 | |
| 0.6303983447612054 | 0.1853570111591773 | 0.1924931466094191 | |
| 0.9628761505104722 | 0.3854343639916520 | 0.1924398886597596 | |
| 0.9635641470745577 | 0.7846198323318371 | 0.1922272788887951 | |
| 0.6311408865691959 | 0.7841419341854443 | 0.1933733129775871 | |
| 0.6302677306848115 | -0.0155347703725145 | 0.1928975057633548 | T |
| 0.9639243278194976 | 0.5847767100854456 | 0.1902071136355034 | |
| 0.9637948678476915 | -0.0150258756133087 | 0.1925549453830350 | T |
| 0.9633576528223483 | 0.1845654059981083 | 0.1927630520070495 | |
| 0.6302818831491961 | 0.3846952645689807 | 0.1928799151165917 | |
| 0.2962689875958119 | 0.5856137066988879 | 0.1931399578631726 | |
| 0.2966868790682471 | -0.2151261751430397 | 0.1932774644907534 | T |
| 0.2974060780515255 | 0.1853867996255478 | 0.1931125151336550 | |
| 0.2972218098841796 | -0.0145220227849763 | 0.1931873211445132 | T |
| 0.2971230495492616 | 0.3849062592829776 | 0.1933697943162486 | |
| 0.8101825287678157 | 0.2373447505850108 | 0.2077702467421748 | |
| 0.4770474626983281 | -0.1620307748499675 | 0.2080120335416955 | T |
| 0.1437332915179911 | -0.1609473191506723 | 0.2076691262734544 | T |
| 0.8101725562313694 | 0.0378344915193236 | 0.2082093042185573 | |
| 1.1423816955399340 | 0.6378890980841209 | 0.2079392784352264 | |
| 0.8089570025108201 | 0.8369800670337517 | 0.2089941409577751 | |
| 0.4770035615363003 | 0.0391705155963892 | 0.2083668617391007 | |
| 0.1436019327986945 | 0.0398061212611153 | 0.2081609328031498 | |
| 1.1438246180220877 | 0.4383722683794238 | 0.2084086487053572 | |
| 0.8096895766780668 | 0.4393816382990858 | 0.2085759267333578 | |
| 0.4760786820329945 | 0.6377664056214432 | 0.2084835724648034 | |
| 0.8095714097660421 | 0.6379941405476405 | 0.2089682514027657 | |
| 0.1436610110138121 | 0.2395633206648729 | 0.2084808582134686 | |
| 0.4769638291264567 | 0.2401582293610277 | 0.2093280933790447 | |
| 0.4773152857045396 | 0.4392058444393003 | 0.2102183343845543 | |
| 0.7066917764535920 | 0.3050086204208651 | 0.2852688895212684 | |
| 0.3704931692836700 | 0.7091090397707063 | 0.2853946770253907 | |
| 0.7053590316690584 | 0.1064554938677736 | 0.2856024259775930 | |
| 1.0371919681261161 | 0.3071644980247569 | 0.2852711765607558 | |
| 1.0398785452972144 | 0.9083686722580352 | 0.2854177179926642 | |
| 0.3720620958733878 | 0.1066550252440982 | 0.2854956458886649 | |
| 1.0346433607643395 | 0.5064300664642054 | 0.2850111315810418 | |

| | | | |
|---|---|---|---|
| 1.0368965826703236 | 0.7060385904560486 | 0.2846697363613455 | |
| 0.7054959517373263 | 0.7053244586433830 | 0.2895765286272147 | |
| 0.7055856691465008 | -0.0922979337428177 | 0.2861718086327334 | T |
| 0.3717009217735232 | -0.0924306918477063 | 0.2857448867589737 | T |
| 1.0381319063839312 | 0.1073824688717343 | 0.2858253820584383 | |
| 0.7080523252520212 | 0.5099780123432928 | 0.2854822035134171 | |
| 0.3670480181932553 | 0.5081472930629136 | 0.2879984945907061 | |
| 0.3690653073822288 | 0.3067548714607118 | 0.2889685680505633 | |
| 0.5532319443595968 | 0.7642021867553324 | 0.2984126139907572 | |
| 0.5542694502571057 | 0.1578733775641010 | 0.2986273646859914 | |
| 0.8864555853771197 | 0.3591621097738715 | 0.2991009622904500 | |
| 0.8869520787093232 | 0.5626912217295255 | 0.3054650264934874 | |
| 0.2148736683600896 | 0.5660233923843636 | 0.2988823437995823 | |
| 0.8893182770558801 | 0.7659224589821124 | 0.2981708239025295 | |
| 0.5539554825247237 | -0.0384712478896051 | 0.2991430058784192 | T |
| 0.2176728654788450 | 0.1569838321701551 | 0.2989840584725746 | |
| 0.2187371074726122 | -0.2366767030689431 | 0.2991142489944718 | T |
| 0.8877567293020965 | -0.0373246986655485 | 0.2988298644429387 | T |
| 0.2200576763179076 | -0.0384092227859144 | 0.2993324704350945 | T |
| 0.8866289943052335 | 0.1618825965661646 | 0.2992501934189594 | |
| 0.2143896591084110 | 0.3600096813461536 | 0.2993562455463313 | |
| 0.5475304549512598 | 0.5639071754924136 | 0.3010218092293143 | |
| 0.5532165885010942 | 0.3597013665699707 | 0.3069090388678960 | |

Ru: Final state state for $ArCHCH_2(COOMe)$ and H coupling
($E$ = -1237.0545 eV, $ZPE$ = 5.09127748449999 eV, $S*T$ = 1.34305989312597 eV)

```
AutoCreatByScript: H C O Ru
   1.00000000000000
    15.1228015700000000    0.0000000000000000    0.0000000000000000
    -3.6418149199999998   13.0568463000000001    0.0000000000000000
     0.0000000000000000    0.0000000000000000   21.5088567099999999
   H    C    O    Ru
    12    10     2   120
Selective dynamics
Direct
  0.6081270519746418  0.3505095033746178  0.3849864470378817
  0.8661942620550864  0.7121191881081281  0.3935329330269666
  0.4927167834949224  0.5645857234333767  0.3994556170134839
  0.4280933217860673  0.2335604703478419  0.4018573335739388
  0.2656003525501275  0.2228147004809659  0.4025726179533635
  0.3319897249026914  0.5523133673596795  0.4090815504183398
  0.8001366414043374  0.7993469190134294  0.4192100360385057
  0.2198782397701460  0.3831365813696712  0.4221672638429747
  0.6064514976180664  0.5536156858908167  0.4514221386289752
  0.5555500297710371  0.3457768302019587  0.4562719189515862
  0.8061118092275138  0.6893469658836506  0.4655815338349479
  0.6830449639484271  0.4807603141006503  0.4716935822223457
  0.7076949485851842  0.5508656284809756  0.3822546751943277
  0.4466985841064234  0.4926666360142785  0.3812945447688754
  0.3119821963815840  0.2959715781526186  0.3868147287030623
  0.4083648887171919  0.3017372538913271  0.3872046804767589
  0.3491791204476409  0.4863845644845112  0.3880955008916336
  0.4790839656749829  0.3993362678410793  0.3866723153991256
  0.2794401653356548  0.3875625644533383  0.3921791931516074
  0.5699747085427540  0.3978347336803904  0.4159130742115664
  0.8030484321513294  0.7195979420590960  0.4184148406276897
  0.6409101046959074  0.4978419518995133  0.4334280649823858
  0.7853267740689169  0.5219386748268758  0.3775933638288957
  0.7205742162328695  0.6631094044901379  0.3872646791926840
  0.1407055901600031  0.1380862835040000  0.0000000000000000
  0.4740389234929976  0.1380862835040000  0.0000000000000000
  0.8073722568259991  0.1380862835040000  0.0000000000000000
  0.1407055901600030  0.3380862835039917  0.0000000000000000
  0.4740389234929968  0.3380862835039917  0.0000000000000000
  0.8073722568259991  0.3380862835039917  0.0000000000000000
  0.1407055901600029  0.5380862835039926  0.0000000000000000
  0.4740389234929976  0.5380862835039926  0.0000000000000000
  0.8073722568259993  0.5380862835039926  0.0000000000000000
```

| | | |
|---|---|---|
| 0.1407055901600020 | 0.7380862835039907 | 0.0000000000000000 |
| 0.4740389234929979 | 0.7380862835039907 | 0.0000000000000000 |
| 0.8073722568259991 | 0.7380862835039907 | 0.0000000000000000 |
| 0.1407055901600017 | 0.9380862835039834 | 0.0000000000000000 |
| 0.4740389234929964 | 0.9380862835039834 | 0.0000000000000000 |
| 0.8073722568259988 | 0.9380862835039834 | 0.0000000000000000 |
| 0.3201838529240039 | 0.1920708306439959 | 0.0159535054489979 |
| 0.6535171862569976 | 0.1920708306439959 | 0.0159535054489979 |
| 0.9868505195899991 | 0.1920708306439959 | 0.0159535054489979 |
| 0.3201838529240035 | 0.3920708306439959 | 0.0159535054489979 |
| 0.6535171862569981 | 0.3920708306439959 | 0.0159535054489979 |
| 0.9868505195900008 | 0.3920708306439959 | 0.0159535054489979 |
| 0.3201838529240035 | 0.5920708306439887 | 0.0159535054489979 |
| 0.6535171862569977 | 0.5920708306439887 | 0.0159535054489979 |
| 0.9868505195899988 | 0.5920708306439887 | 0.0159535054489979 |
| 0.3201838529240035 | 0.7920708306439878 | 0.0159535054489979 |
| 0.6535171862569977 | 0.7920708306439878 | 0.0159535054489979 |
| 0.9868505195900009 | 0.7920708306439878 | 0.0159535054489979 |
| 0.3201838529240028 | 0.9920708306439862 | 0.0159535054489979 |
| 0.6535171862569978 | 0.9920708306439862 | 0.0159535054489979 |
| 0.9868505195900009 | 0.9920708306439862 | 0.0159535054489979 |
| 0.2186901232590020 | 0.0615218340180011 | 0.0955531191689971 |
| 0.5520234565920040 | 0.0615218340180011 | 0.0955531191689971 |
| 0.8853567899249992 | 0.0615218340180011 | 0.0955531191689971 |
| 0.2186901232590017 | 0.2615218340179939 | 0.0955531191689971 |
| 0.5520234565920039 | 0.2615218340179939 | 0.0955531191689971 |
| 0.8853567899249992 | 0.2615218340179939 | 0.0955531191689971 |
| 0.2186901232590017 | 0.4615218340179942 | 0.0955531191689971 |
| 0.5520234565920040 | 0.4615218340179942 | 0.0955531191689971 |
| 0.8853567899249994 | 0.4615218340179942 | 0.0955531191689971 |
| 0.2186901232590009 | 0.6615218340179913 | 0.0955531191689971 |
| 0.5520234565920039 | 0.6615218340179913 | 0.0955531191689971 |
| 0.8853567899249991 | 0.6615218340179913 | 0.0955531191689971 |
| 0.2186901232590008 | 0.8615218340179863 | 0.0955531191689971 |
| 0.5520234565920035 | 0.8615218340179863 | 0.0955531191689971 |
| 0.8853567899249959 | 0.8615218340179863 | 0.0955531191689971 |
| 0.0648350526889984 | 0.1155063811579976 | 0.1115066246180021 |
| 0.3981683860230021 | 0.1155063811579976 | 0.1115066246180021 |
| 0.7315017193559983 | 0.1155063811579976 | 0.1115066246180021 |
| 0.0648350526889978 | 0.3155063811579962 | 0.1115066246180021 |
| 0.3981683860230020 | 0.3155063811579962 | 0.1115066246180021 |
| 0.7315017193559979 | 0.3155063811579962 | 0.1115066246180021 |
| 0.0648350526889977 | 0.5155063811579899 | 0.1115066246180021 |
| 0.3981683860230019 | 0.5155063811579899 | 0.1115066246180021 |

| | | |
|---|---|---|
| 0.7315017193559954 | 0.5155063811579899 | 0.1115066246180021 |
| 0.0648350526889973 | 0.7155063811579874 | 0.1115066246180021 |
| 0.3981683860230018 | 0.7155063811579874 | 0.1115066246180021 |
| 0.7315017193559961 | 0.7155063811579874 | 0.1115066246180021 |
| 0.0648350526889969 | 0.9155063811579812 | 0.1115066246180021 |
| 0.3981683860230018 | 0.9155063811579812 | 0.1115066246180021 |
| 0.7315017193559956 | 0.9155063811579812 | 0.1115066246180021 |
| 0.9630059972156535 | 0.5844663602720731 | 0.1919697254881338 |
| 0.9632531614147863 | 0.7847133107167844 | 0.1922316948278515 |
| 0.6303743986707857 | 0.3854040100488663 | 0.1926331186904188 |
| 0.9634391887734779 | 0.3853167940638696 | 0.1926395303448984 |
| 0.9631572247025129 | 0.1847506594918687 | 0.1926258085359761 |
| 0.6306202249434690 | 0.1855763234466113 | 0.1924949002495389 |
| 0.9639406586182804 | 0.9848778197691896 | 0.1927172007067399 |
| 0.6308648926428864 | 0.5841792875234905 | 0.1928146762313848 |
| 0.2966043039859975 | 0.5851171783464509 | 0.1930494449617061 |
| 0.2968366421874627 | 0.7847118514447158 | 0.1928872860804677 |
| 0.2970241879274089 | 0.9849334826760373 | 0.1929894571856383 |
| 0.6302300933325530 | 0.9844946819725218 | 0.1929396261974872 |
| 0.6307740083149134 | 0.7847117016012712 | 0.1930052540798657 |
| 0.2968663026027313 | 0.1847168063730345 | 0.1934578699648607 |
| 0.2962683396353537 | 0.3852415858044251 | 0.1936435840589323 |
| 0.1436118949910796 | 0.2386100227803515 | 0.2080325189922415 |
| 0.1429942554397087 | 0.8385245213883263 | 0.2079233608479724 |
| 0.4769353065367021 | 0.0381505218750677 | 0.2079636410936149 |
| 0.8105045183755004 | 0.0380062019792155 | 0.2081194138567377 |
| 0.8102909427356956 | 0.2378755877955703 | 0.2081849879171866 |
| 0.8104248104684297 | 0.6379622288336092 | 0.2084210728308577 |
| 0.4769763774714498 | 0.8379576722958224 | 0.2081098608677723 |
| 0.8099299159222507 | 0.4384121352878961 | 0.2083966817364442 |
| 0.1431920576355918 | 0.6380439744885866 | 0.2082659049159413 |
| 0.1432449803824098 | 0.4385447833900807 | 0.2084460869482334 |
| 0.1436321852649654 | 0.0389415060853653 | 0.2085536445712279 |
| 0.4773777605506826 | 0.2397635689673707 | 0.2092996976656656 |
| 0.4764363664789987 | 0.6368099526470502 | 0.2085713888683637 |
| 0.8087820940246495 | 0.8372085076566143 | 0.2088477581769249 |
| 0.4756099445636692 | 0.4374548313024135 | 0.2090595620286307 |
| 0.3724914359970181 | 0.1050464319851586 | 0.2851052703435375 |
| 0.0382390161841331 | 0.3072760612527470 | 0.2853513911254103 |
| 0.0368783228781847 | 0.5060786033843284 | 0.2856181788603169 |
| 0.0377693839032094 | 0.7061080302260654 | 0.2856013970586472 |
| 0.7052891762991506 | 0.1067448686961361 | 0.2854699295306329 |
| 0.7046758406484099 | 0.3045809257947202 | 0.2847199237515345 |
| 0.3715406167125622 | 0.7089563914186511 | 0.2855726386974809 |

| | | |
|---|---|---|
| 0.3718405738652394 | 0.9067532926206695 | 0.2856303756693785 |
| 0.0379656849829329 | 0.1074472171442862 | 0.2858321240777840 |
| 0.0396456546456768 | 0.9078128601705309 | 0.2857998349241027 |
| 0.7032266263536797 | 0.5079606841017628 | 0.2874353164824673 |
| 0.7054126492988839 | 0.9079048516128524 | 0.2860572607457834 |
| 0.3681476436003241 | 0.3020908470973728 | 0.2872595724280556 |
| 0.7058106547902550 | 0.7080609633026762 | 0.2875248775572788 |
| 0.3704625403609493 | 0.5086637453622203 | 0.2883277734186719 |
| 0.8892378306625766 | 0.7648844106721450 | 0.2981755358724524 |
| 0.5525026795100983 | 0.7635984303424893 | 0.2984568764538498 |
| 0.5542254032982743 | 0.1585914073708022 | 0.2985663865957631 |
| 0.8876982715481359 | 0.9630774939886569 | 0.2989026759591547 |
| 0.2166970803018434 | 0.5657475746923391 | 0.2988752974614935 |
| 0.8865502482915356 | 0.3598296284784163 | 0.2991393536662285 |
| 0.2200638386915456 | 0.9607027995709946 | 0.2991403897177925 |
| 0.8867779841287906 | 0.1614593359202510 | 0.2992744682390847 |
| 0.2195336454854422 | 0.7627354959583471 | 0.2990743064496603 |
| 0.5511077986364152 | 0.5659372973733837 | 0.2996490784885767 |
| 0.5537241408836330 | 0.9618173582379539 | 0.2994234863219685 |
| 0.2167400822377322 | 0.1552327228283158 | 0.2998221441103499 |
| 0.2143121383297426 | 0.3623252676107231 | 0.3017103018476909 |
| 0.8888771616854652 | 0.5629351363514113 | 0.3029278349417504 |
| 0.5510697570906099 | 0.3577581488735638 | 0.3050423409491831 |

Ru: Initial state state for ArCH(COOMe)$CH_2$ and H coupling

(*E* -1236.7738 eV, *ZPE* = 4.9261704885 eV, *S***T* = 1.38575945360879 eV)

AutoCreatByScript: H C O Ru
1.00000000000000
15.1228015700000000 0.0000000000000000 0.0000000000000000
-3.6418149199999998 13.0568463000000001 0.0000000000000000
0.0000000000000000 0.0000000000000000 21.5088567099999999
H C O Ru
12 10 2 120
Selective dynamics
Direct
0.7990144712016302 0.6351523013640409 0.3238557790921574
0.4759096376109092 0.5462312942130567 0.4027038635777714
0.6860557669081382 0.4833249901008155 0.3968001492509544
0.3866086988797925 0.2185758306815772 0.4041734054347191
0.3218762299178182 0.5563965996254282 0.4098515117415188
0.2265499483052785 0.2278234914066216 0.4117260998271420
0.1946783493372354 0.3978455820624315 0.4228320700323842
0.6030086445629108 0.5517530448558624 0.3969663600604055
0.6885503271801461 0.1670254075403162 0.4249526394937086
0.7834962318712346 0.2773733251606171 0.4397451548402171
0.5464853491891102 0.3863931788170200 0.4581708052285503
0.6783262083487228 0.2585281340404356 0.4835538521633130
0.4250893102632717 0.4801132384860465 0.3834523886399012
0.4472072244809463 0.3801142835698677 0.3843168444416548
0.3313600360457056 0.4853439410444726 0.3905489021896915
0.3716267332949449 0.2893351384502211 0.3893549324147179
0.5783747015689239 0.2892779187101822 0.3849467041558815
0.6166665723096759 0.4801973643324771 0.3794485229447452
0.2773453256351718 0.2958159447940792 0.3941519956278658
0.2562992959331996 0.3926150316453645 0.3981743756353912
0.5456837740903884 0.3835629664706275 0.4065320017576851
0.7085902402683438 0.2481796027514797 0.4383763164756480
0.6787557283336421 0.3105916755687345 0.3922818254775154
0.5333646123931122 0.1948724498180522 0.3971141747483283
0.1407055901600970 0.1380862835040199 0.0000000000000000
0.4740389234927777 0.1380862835040199 0.0000000000000000
0.8073722568255945 0.1380862835040199 0.0000000000000000
0.1407055901609501 0.3380862835039297 0.0000000000000000
0.4740389234927747 0.3380862835039297 0.0000000000000000
0.8073722568259011 0.3380862835039297 0.0000000000000000
0.1407055901599990 0.5380862835040792 0.0000000000000000
0.4740389234928012 0.5380862835040792 0.0000000000000000
0.8073722568260924 0.5380862835040792 0.0000000000000000

| | | |
|---|---|---|
| 0.1407055901599334 | 0.7380862835032062 | 0.0000000000000000 |
| 0.4740389234930277 | 0.7380862835032062 | 0.0000000000000000 |
| 0.8073722568252162 | 0.7380862835032062 | 0.0000000000000000 |
| 0.1407055901597954 | 0.9380862835041772 | 0.0000000000000000 |
| 0.4740389234925578 | 0.9380862835041772 | 0.0000000000000000 |
| 0.8073722568266777 | 0.9380862835041772 | 0.0000000000000000 |
| 0.3201838529237411 | 0.1920708306439943 | 0.0159535054489979 |
| 0.6535171862582497 | 0.1920708306439943 | 0.0159535054489979 |
| 0.9868505195887536 | 0.1920708306439943 | 0.0159535054489979 |
| 0.3201838529240688 | 0.3920708306438155 | 0.0159535054489979 |
| 0.6535171862571523 | 0.3920708306438155 | 0.0159535054489979 |
| 0.9868505195900884 | 0.3920708306438155 | 0.0159535054489979 |
| 0.3201838529240035 | 0.5920708306435506 | 0.0159535054489979 |
| 0.6535171862569916 | 0.5920708306435506 | 0.0159535054489979 |
| 0.9868505195897481 | 0.5920708306435506 | 0.0159535054489979 |
| 0.3201838529237477 | 0.7920708306439772 | 0.0159535054489979 |
| 0.6535171862566179 | 0.7920708306439772 | 0.0159535054489979 |
| 0.9868505195900010 | 0.7920708306439772 | 0.0159535054489979 |
| 0.3201838529241964 | 0.9920708306452186 | 0.0159535054489979 |
| 0.6535171862564080 | 0.9920708306434328 | 0.0159535054489979 |
| 0.9868505195906592 | 0.9920708306434328 | 0.0159535054489979 |
| 0.2186901232588694 | 0.0615218340179681 | 0.0955531191689971 |
| 0.5520234565910362 | 0.0615218340179681 | 0.0955531191689971 |
| 0.8853567899250356 | 0.0615218340179681 | 0.0955531191689971 |
| 0.2186901232588606 | 0.2615218340180336 | 0.0955531191689971 |
| 0.5520234565923413 | 0.2615218340180336 | 0.0955531191689971 |
| 0.8853567899257324 | 0.2615218340180336 | 0.0955531191689971 |
| 0.2186901232591009 | 0.4615218340178255 | 0.0955531191689971 |
| 0.5520234565911807 | 0.4615218340178255 | 0.0955531191689971 |
| 0.8853567899255904 | 0.4615218340178255 | 0.0955531191689971 |
| 0.2186901232588392 | 0.6615218340178596 | 0.0955531191689971 |
| 0.5520234565908002 | 0.6615218340178596 | 0.0955531191689971 |
| 0.8853567899254560 | 0.6615218340178596 | 0.0955531191689971 |
| 0.2186901232580557 | 0.8615218340176555 | 0.0955531191689971 |
| 0.5520234565915989 | 0.8615218340176555 | 0.0955531191689971 |
| 0.8853567899250854 | 0.8615218340176555 | 0.0955531191689971 |
| 0.0648350526889985 | 0.1155063811578972 | 0.1115066246180021 |
| 0.3981683860227253 | 0.1155063811578972 | 0.1115066246180021 |
| 0.7315017193560439 | 0.1155063811578972 | 0.1115066246180021 |
| 0.0648350526889702 | 0.3155063811584176 | 0.1115066246180021 |
| 0.3981683860229169 | 0.3155063811584176 | 0.1115066246180021 |
| 0.7315017193558551 | 0.3155063811584176 | 0.1115066246180021 |
| 0.0648350526890769 | 0.5155063811580765 | 0.1115066246180021 |
| 0.3981683860232990 | 0.5155063811580765 | 0.1115066246180021 |

| | | |
|---|---|---|
| 0.7315017193555857 | 0.5155063811580765 | 0.1115066246180021 |
| 0.0648350526888646 | 0.7155063811574039 | 0.1115066246180021 |
| 0.3981683860229656 | 0.7155063811574039 | 0.1115066246180021 |
| 0.7315017193561566 | 0.7155063811574039 | 0.1115066246180021 |
| 0.0648350526891310 | 0.9155063811577384 | 0.1115066246180021 |
| 0.3981683860229881 | 0.9155063811577384 | 0.1115066246180021 |
| 0.7315017193561940 | 0.9155063811577384 | 0.1115066246180021 |
| 0.9637651479324418 | 0.3847841647886946 | 0.1924534117430367 |
| 0.9635415697408207 | 0.7848404754751528 | 0.1925342029281902 |
| 0.9642049237619827 | 0.5850279785344327 | 0.1923529225857087 |
| 0.6307537977667985 | 0.3850128639662764 | 0.1919473301058054 |
| 0.9630369743387702 | 0.1851631603034423 | 0.1928493411822828 |
| 0.6310166159068886 | 0.5852498364389537 | 0.1925950275237984 |
| 0.6303876532616587 | 0.7853819105746673 | 0.1926472412557189 |
| 0.6300329761986656 | 0.9851822823472484 | 0.1929513601872074 |
| 0.9632068315643727 | 0.9847943373430136 | 0.1928905387978921 |
| 0.2971153447200174 | 0.5846050751834780 | 0.1931001327145845 |
| 0.2969453992115337 | 0.7850361401323840 | 0.1929145373546253 |
| 0.2969294267963530 | 0.9846680959152324 | 0.1930288695899235 |
| 0.6306470963107492 | 0.1854235769485670 | 0.1932601975150628 |
| 0.2969525105471105 | 0.3847446495079229 | 0.1936780969569005 |
| 0.2974163427230661 | 0.1852240297107141 | 0.1937431065916328 |
| 0.8099156573375288 | 0.6375038517042472 | 0.2071979398892488 |
| 0.1429135608165985 | 0.8381827184863864 | 0.2081288676134792 |
| 0.4764937004949588 | 0.8387993309547743 | 0.2081584542876479 |
| 0.8100844778668401 | 0.8386552233672947 | 0.2084242810100160 |
| 0.8100280929579328 | 0.0383044699195506 | 0.2084028429161983 |
| 0.4776400563956356 | 0.0399953257061272 | 0.2088682300137616 |
| 0.1439063232364728 | 0.2381030485070098 | 0.2081694550882603 |
| 0.4741573210601113 | 0.4394174889181409 | 0.2076230351699528 |
| 0.1440825292147216 | 0.6375148919765140 | 0.2085697560873594 |
| 0.8084458041739656 | 0.2391396691720948 | 0.2091216329432216 |
| 0.1445263110471486 | 0.4376000460302911 | 0.2086935854450858 |
| 0.1437471324754927 | 0.0391604779082714 | 0.2086709292309145 |
| 0.4765388481735525 | 0.6371599246089990 | 0.2089069861598999 |
| 0.4772925805440102 | 0.2393755855945309 | 0.2089900089442578 |
| 0.8080178808343046 | 0.4370569713819504 | 0.2095437435597287 |
| 0.3697752689067280 | 0.1041928657509069 | 0.2852141068899305 |
| 0.7041890064970749 | 0.1057754466921071 | 0.2855476718576352 |
| 0.3725149746498539 | 0.7095548091510998 | 0.2855488754246971 |
| 0.0388281745668699 | 0.5072234308214927 | 0.2855973547004461 |
| 0.7060771753473245 | 0.7103223724629231 | 0.2860036860090344 |
| 0.0366750905774147 | 0.1071921369110804 | 0.2856720415101324 |
| 0.3710875687826113 | 0.9068529047758147 | 0.2857264450153001 |

| | | |
|---|---|---|
| 0.0378047396923698 | 0.9069925622014500 | 0.2858229211023409 |
| 0.0397907487532743 | 0.7079749194232827 | 0.2859182199983804 |
| 0.7055866530569943 | 0.9079059620081973 | 0.2859156255501693 |
| 0.0378786722982904 | 0.3064420656230964 | 0.2858574815247031 |
| 0.7067336293971032 | 0.5096993564692485 | 0.2877229825551521 |
| 0.3738852424589597 | 0.3050560525091010 | 0.2881499052304028 |
| 0.7082978864034637 | 0.3059385603639396 | 0.2890545349423701 |
| 0.3716677538741405 | 0.5082764190084782 | 0.2900031835770175 |
| 0.8890508230384092 | 0.3628153722875191 | 0.2985876477052493 |
| 0.2185654211595809 | 0.5651367651873497 | 0.2986885888064401 |
| 0.5539777197166869 | 0.7631364620213962 | 0.2985779317908613 |
| 0.5535119670127276 | 0.9601320062214219 | 0.2992666454714173 |
| 0.8860337049363288 | 0.1616137260952723 | 0.2992888260335952 |
| 0.2193783816823980 | 0.9607743979866502 | 0.2992167988808015 |
| 0.8867528398198489 | 0.9621521410506041 | 0.2993908534882996 |
| 0.2164016675276775 | 0.1570267684549915 | 0.2994298125811111 |
| 0.2200209481187609 | 0.7621033265443743 | 0.2994995617270563 |
| 0.5560650371506605 | 0.5666431313672312 | 0.2992262817535273 |
| 0.8896597721766001 | 0.5619642566455456 | 0.2992478135263747 |
| 0.5564757733622123 | 0.3630347162170647 | 0.2985954222774541 |
| 0.8880856940238296 | 0.7629459809542619 | 0.3001612937826714 |
| 0.5474399042287106 | 0.1577481800044263 | 0.3019332869905579 |
| 0.2150661570517867 | 0.3594223970426653 | 0.3023031379805345 |

Ru: Transition state state for ArCH(COOMe)$CH_2$ and H coupling

($E$ = -1235.7104 eV, $ZPE$ = 4.92177565499999 eV, $S*T$ = 1.37727084756014 eV)

```
AutoCreatByScript: H C O Ru
   1.00000000000000
    15.1228015700000000    0.0000000000000000    0.0000000000000000
    -3.6418149199999998   13.0568463000000001    0.0000000000000000
     0.0000000000000000    0.0000000000000000   21.5088567099999999
   H    C    O    Ru
    12   10    2  120
Selective dynamics
Direct
  0.6526957111158911  0.5746510980279479  0.3500282722142252
  0.4803119677632395  0.5547777778208467  0.3997140633736603
  0.6906599395262820  0.4774002746887330  0.4051128960513469
  0.3918536308318931  0.2268053162249209  0.4044552184565812
  0.3247786183042595  0.5629453694574631  0.4079371839243189
  0.2314986347207312  0.2342171309055850  0.4122899769449489
  0.1988553670884362  0.4040288416041374  0.4227198609895612
  0.6163374208885033  0.5511869775172281  0.4296496103972187
  0.6884081618754628  0.1671216533268513  0.4299626303282197
  0.7800784822537365  0.2785971065988515  0.4495678243827466
  0.5463923785248741  0.3664813862715836  0.4525744104972403
  0.6692449832992645  0.2571520550983111  0.4864574018437015
  0.4290987361925073  0.4879983620748380  0.3807777018629783
  0.4511910101689635  0.3885674643942025  0.3836411219674582
  0.3345403206088096  0.4920952595578720  0.3885100815055084
  0.3759829519914361  0.2967881795584102  0.3890345048707640
  0.5848519268123730  0.2858128739041449  0.3799623986422433
  0.6236366695446489  0.4921319827306019  0.3965706292422480
  0.2816822015557067  0.3023527160380994  0.3943744528610341
  0.2600940245162998  0.3987519626949844  0.3976162077120606
  0.5480088527537856  0.3911926979204151  0.4035165344649487
  0.7058800158691191  0.2480535364879695  0.4435990441280892
  0.6813130646078251  0.3103479220716505  0.3949721737867256
  0.5361002032179162  0.1965979275525165  0.3981931696746457
  0.1407055901600567  0.1380862835039474  0.0000000000000000
  0.4740389234929795  0.1380862835039474  0.0000000000000000
  0.8073722568262085  0.1380862835039474  0.0000000000000000
  0.1407055901609773  0.3380862835039840  0.0000000000000000
  0.4740389234930552  0.3380862835039840  0.0000000000000000
  0.8073722568261485  0.3380862835039840  0.0000000000000000
  1.1407055901601297  0.5380862835037752  0.0000000000000000
  0.4740389234930317  0.5380862835037752  0.0000000000000000
  0.8073722568260653  0.5380862835037752  0.0000000000000000
```

| | | | |
|---|---|---|---|
| 1.1407055901599403 | 0.7380862835037176 | 0.0000000000000000 | |
| 0.4740389234930995 | 0.7380862835037176 | 0.0000000000000000 | |
| 0.8073722568260457 | 0.7380862835037176 | 0.0000000000000000 | |
| 1.1407055901594552 | 0.9380862835037282 | 0.0000000000000000 | |
| 0.4740389234931193 | 0.9380862835037282 | 0.0000000000000000 | |
| 0.8073722568258801 | 0.9380862835037282 | 0.0000000000000000 | |
| 0.3201838529240384 | 0.1920708306439835 | 0.0159535054489979 | |
| 0.6535171862580356 | 0.1920708306439835 | 0.0159535054489979 | |
| 0.9868505195891355 | 0.1920708306439835 | 0.0159535054489979 | |
| 0.3201838529239434 | 0.3920708306439518 | 0.0159535054489979 | |
| 0.6535171862572209 | 0.3920708306439518 | 0.0159535054489979 | |
| 0.9868505195899140 | 0.3920708306439518 | 0.0159535054489979 | |
| 0.3201838529240035 | 0.5920708306438688 | 0.0159535054489979 | |
| 0.6535171862570449 | 0.5920708306438688 | 0.0159535054489979 | |
| 0.9868505195900116 | 0.5920708306438688 | 0.0159535054489979 | |
| 0.3201838529240059 | 0.7920708306437820 | 0.0159535054489979 | |
| 0.6535171862573990 | 0.7920708306437820 | 0.0159535054489979 | |
| 0.9868505195902231 | 0.7920708306437820 | 0.0159535054489979 | |
| 0.3201838529240065 | -0.0079291693561326 | 0.0159535054489979 | F |
| 0.6535171862571099 | 0.9920708306429984 | 0.0159535054489979 | |
| 0.9868505195900468 | 0.9920708306429984 | 0.0159535054489979 | |
| 0.2186901232590252 | 0.0615218340180142 | 0.0955531191689971 | |
| 0.5520234565909872 | 0.0615218340180142 | 0.0955531191689971 | |
| 0.8853567899256268 | 1.0615218340185235 | 0.0955531191689971 | |
| 0.2186901232590331 | 0.2615218340178888 | 0.0955531191689971 | |
| 0.5520234565920610 | 0.2615218340178888 | 0.0955531191689971 | |
| 0.8853567899248875 | 0.2615218340178888 | 0.0955531191689971 | |
| 0.2186901232589631 | 0.4615218340180085 | 0.0955531191689971 | |
| 0.5520234565919568 | 0.4615218340180085 | 0.0955531191689971 | |
| 0.8853567899248784 | 0.4615218340180085 | 0.0955531191689971 | |
| 0.2186901232589959 | 0.6615218340179048 | 0.0955531191689971 | |
| 0.5520234565910407 | 0.6615218340179048 | 0.0955531191689971 | |
| 0.8853567899250798 | 0.6615218340179048 | 0.0955531191689971 | |
| 1.2186901232581095 | 0.8615218340178284 | 0.0955531191689971 | |
| 0.5520234565920541 | 0.8615218340178284 | 0.0955531191689971 | |
| 0.8853567899240649 | 0.8615218340178284 | 0.0955531191689971 | |
| 1.0648350526893799 | 1.1155063811585295 | 0.1115066246180021 | |
| 0.3981683860230169 | 0.1155063811579831 | 0.1115066246180021 | |
| 0.7315017193561762 | 0.1155063811579831 | 0.1115066246180021 | |
| 1.0648350526890160 | 0.3155063811578807 | 0.1115066246180021 | |
| 0.3981683860228455 | 0.3155063811578807 | 0.1115066246180021 | |
| 0.7315017193561942 | 0.3155063811578807 | 0.1115066246180021 | |
| 1.0648350526889976 | 0.5155063811577725 | 0.1115066246180021 | |
| 0.3981683860230213 | 0.5155063811577725 | 0.1115066246180021 | |

| | | | |
|---|---|---|---|
| 0.7315017193560065 | 0.5155063811577725 | 0.1115066246180021 | |
| 1.0648350526886174 | 0.7155063811581660 | 0.1115066246180021 | |
| 0.3981683860230540 | 0.7155063811581660 | 0.1115066246180021 | |
| 0.7315017193559734 | 0.7155063811581660 | 0.1115066246180021 | |
| 1.0648350526892405 | 0.9155063811570047 | 0.1115066246180021 | |
| 0.3981683860230048 | 0.9155063811570047 | 0.1115066246180021 | |
| 0.7315017193558611 | 0.9155063811570047 | 0.1115066246180021 | |
| 0.9636253431828834 | 0.3848081998876397 | 0.1923093989479739 | |
| 0.9631046641543595 | 0.7845118098970758 | 0.1927388443834738 | |
| 0.9636404875456255 | 0.5849355724818043 | 0.1926215774236919 | |
| 0.6304928423731140 | 0.3846902349567925 | 0.1921330265641658 | |
| 0.9629333012523809 | 0.1851524379826501 | 0.1927095107507009 | |
| 0.6303793595862177 | 0.5843459646646481 | 0.1929067529869710 | |
| 0.6303204406415572 | 0.7850251682731686 | 0.1925237891301397 | |
| 0.6299319108173214 | 0.9849457846941444 | 0.1927743724818419 | |
| 0.9630661138795080 | 0.9848023821780656 | 0.1928553832270875 | |
| 0.2967990883686553 | 0.5848635845236830 | 0.1929364120537703 | |
| 0.2970531880372997 | 0.7849389524330728 | 0.1928730507001284 | |
| 0.2968658363745540 | -0.0152237359852764 | 0.1929224084572417 | T |
| 0.6305735928565810 | 0.1848152001700271 | 0.1932499523978632 | |
| 0.2963715712624290 | 0.3845636689437025 | 0.1934884100284480 | |
| 0.2972273930059570 | 0.1851785456154801 | 0.1935982700600689 | |
| 0.8093298191047061 | 0.6375834890675823 | 0.2084039639219517 | |
| 1.1428469241483268 | 0.8381437091337476 | 0.2080410221376709 | |
| 0.4766440592093161 | 0.8386528731654265 | 0.2080686929037188 | |
| 0.8097065824338562 | 0.8382489445425330 | 0.2083509108526734 | |
| 0.8099689845368816 | 1.0381651853263856 | 0.2083468521603464 | |
| 0.4773281025415498 | 0.0393529276638176 | 0.2085458798283012 | |
| 0.1436856022272826 | 0.2380300379686212 | 0.2080635725412449 | |
| 0.4740332805271753 | 0.4382889303770877 | 0.2074694669170998 | |
| 1.1436964203858115 | 0.6378408353608375 | 0.2083652129077832 | |
| 0.8087912184650081 | 0.2388782707564948 | 0.2087731600110980 | |
| 0.1440024040458518 | 0.4378685075789961 | 0.2085773995178669 | |
| 1.1435110843731646 | 1.0391771695087735 | 0.2087175832361951 | |
| 0.4766227097486976 | 0.6366267478538675 | 0.2085243783574630 | |
| 0.4771738847149169 | 0.2395544246714776 | 0.2090254240806399 | |
| 0.8075392627135626 | 0.4369153093280333 | 0.2095342194578504 | |
| 0.3697959882021467 | 0.1043308064508582 | 0.2851485076693683 | |
| 0.7049063269578876 | 0.1054515407982685 | 0.2853988642222290 | |
| 0.3729513375903352 | 0.7096202961783069 | 0.2852446978522938 | |
| 1.0384874703295801 | 0.5070370706896714 | 0.2855449446053709 | |
| 0.7061590072721349 | 0.7100162845672690 | 0.2860397744678621 | |
| 1.0367739338170323 | 1.1074590963423645 | 0.2856735677619974 | |
| 0.3708897861948425 | 0.9068049977673108 | 0.2856957612606632 | |

| | | |
|---|---|---|
| 1.0380124976675504 | 0.9074088824450335 | 0.2857882938950118 |
| 1.0392120758389773 | 0.7077632143185536 | 0.2858645581060358 |
| 0.7052598533375400 | 0.9069321044687630 | 0.2858784135597038 |
| 1.0380479990081171 | 0.3067061997582589 | 0.2857895455248236 |
| 0.7079116824490088 | 0.5086956558327473 | 0.2879021559763503 |
| 0.3730550442300202 | 0.3052701896757813 | 0.2878102371477421 |
| 0.7090302050572344 | 0.3053848202211462 | 0.2885832386869996 |
| 0.3700156710128697 | 0.5085398221147615 | 0.2884970160817603 |
| 0.8888932254207234 | 0.3629073693080334 | 0.2986963961265796 |
| 0.2177710072654619 | 0.5657583621803021 | 0.2986588812904218 |
| 0.5537817214101810 | 0.7639409871441833 | 0.2986433976248288 |
| 0.5535679151778559 | 0.9600928794218453 | 0.2990536832893494 |
| 0.8861799657604145 | 0.1616812570909491 | 0.2992070639664219 |
| 1.2192819687111496 | 0.9608806673623661 | 0.2991248553118999 |
| 0.8865715939275900 | 0.9621422124023251 | 0.2993924492384852 |
| 0.2161524126370575 | 0.1568763619560001 | 0.2993692912482138 |
| 0.2199640626957914 | 0.7621136324944496 | 0.2992094719917411 |
| 0.5530897424511947 | 0.5654702435684832 | 0.2999775226331976 |
| 0.8887784898695794 | 0.5631020295905442 | 0.2988779241880716 |
| 0.5547630444906704 | 0.3589711616678310 | 0.2997813864544698 |
| 0.8872970866005407 | 0.7621450415988480 | 0.2993240367570819 |
| 0.5484042780595072 | 0.1554890551994159 | 0.3015989330121849 |
| 0.2147229962793112 | 0.3598798944102256 | 0.3025259867550623 |

Ru: Final state state for ArCH(COOMe)$CH_2$ and H coupling
($E$ = -1236.865 eV, $ZPE$ = 5.1493929735 eV, $S*T$ = 1.48245648273949 eV)
AutoCreatByScript: H C O Ru
1.00000000000000
15.1228015700000000 0.0000000000000000 0.0000000000000000
-3.6418149199999998 13.0568463000000001 0.0000000000000000
0.0000000000000000 0.0000000000000000 21.5088567099999999
H C O Ru
12 10 2 120
Selective dynamics
Direct
0.4356934705325813 0.6049308383362487 0.4052646644915771
0.7571221472380379 0.1539818089389538 0.4075275011548428
0.2971244330821840 0.4632566051937868 0.4088392013978738
0.4559285227695382 0.2470083622021752 0.4033138809467479
0.3086366841842201 0.2852710660660466 0.4095516187058446
0.7207942749069991 0.5231244971019460 0.3938890525503220
0.5816477127674314 0.5682626344328059 0.4215335444130500
0.7623240163368307 0.4576609426201596 0.4538150966989470
0.8474771030986850 0.2543550529875962 0.4412368807738519
0.6022897802868238 0.3423704994177061 0.4702732289367770
0.7358254574524977 0.2165233846279862 0.4777402956244603
0.6801295666426038 0.5260186803968109 0.4712608109060614
0.6536160914950901 0.2974565386782851 0.3863187074607226
0.3572814746681496 0.4484583195255743 0.3864030549498348
0.3639680033655474 0.3424684017610882 0.3866385549387253
0.4414180075722305 0.5304936695327823 0.3895726836198496
0.4541078779450580 0.3234938992483244 0.3890093600738020
0.5382270224698982 0.4047247718353648 0.3959472699318907
0.5301791410412904 0.5103544862027943 0.3963275902761547
0.6229991831662414 0.3803592573991075 0.4248419956850975
0.7732688722353248 0.2268962297905147 0.4333848183331254
0.7007176482256290 0.4766214503163501 0.4368606974002823
0.5986556165866899 0.2022890211342784 0.3921036351102477
0.7500593988843351 0.3072061415430891 0.3980851269363072
0.1407055901600090 0.1380862835039923 0.0000000000000000
0.4740389234930064 0.1380862835039923 0.0000000000000000
0.8073722568240146 0.1380862835039923 0.0000000000000000
0.1407055901579997 0.3380862835039966 0.0000000000000000
0.4740389234929924 0.3380862835039966 0.0000000000000000
0.8073722568249713 0.3380862835039966 0.0000000000000000
0.1407055901600086 0.5380862835049637 0.0000000000000000
0.4740389234930137 0.5380862835049637 0.0000000000000000
0.8073722568280020 0.5380862835049637 0.0000000000000000

| | | |
|---|---|---|
| 0.1407055901600049 | 0.7380862835040013 | 0.0000000000000000 |
| 0.4740389234930325 | 0.7380862835040013 | 0.0000000000000000 |
| 0.8073722568259869 | 0.7380862835040013 | 0.0000000000000000 |
| 0.1407055901599948 | 0.9380862835039349 | 0.0000000000000000 |
| 0.4740389234930014 | 0.9380862835039349 | 0.0000000000000000 |
| 0.8073722568259926 | 0.9380862835039349 | 0.0000000000000000 |
| 0.3201838529240124 | 0.1920708306439962 | 0.0159535054489979 |
| 0.6535171862569937 | 0.1920708306439962 | 0.0159535054489979 |
| 0.9868505195870019 | 0.1920708306439962 | 0.0159535054489979 |
| 0.3201838529240029 | 0.3920708306440009 | 0.0159535054489979 |
| 0.6535171862569908 | 0.3920708306440009 | 0.0159535054489979 |
| 0.9868505195890283 | 0.3920708306440009 | 0.0159535054489979 |
| 0.3201838529240035 | 0.5920708306439728 | 0.0159535054489979 |
| 0.6535171862569755 | 0.5920708306439728 | 0.0159535054489979 |
| 0.9868505195900175 | 0.5920708306439728 | 0.0159535054489979 |
| 0.3201838529240111 | 0.7920708306449945 | 0.0159535054489979 |
| 0.6535171862569871 | 0.7920708306439583 | 0.0159535054489979 |
| 0.9868505195919874 | 0.7920708306419231 | 0.0159535054489979 |
| 0.3201838529240119 | 0.9920708306439144 | 0.0159535054489979 |
| 0.6535171862569817 | 0.9920708306439144 | 0.0159535054489979 |
| 0.9868505195890059 | 0.9920708306439144 | 0.0159535054489979 |
| 0.2186901232590025 | 0.0615218340179963 | 0.0955531191689971 |
| 0.5520234565919805 | 0.0615218340179963 | 0.0955531191689971 |
| 0.8853567899250226 | 0.0615218340179963 | 0.0955531191689971 |
| 0.2186901232590013 | 0.2615218340179785 | 0.0955531191689971 |
| 0.5520234565919716 | 0.2615218340179785 | 0.0955531191689971 |
| 0.8853567899249952 | 0.2615218340179785 | 0.0955531191689971 |
| 0.2186901232590018 | 0.4615218340179602 | 0.0955531191689971 |
| 0.5520234565919724 | 0.4615218340179602 | 0.0955531191689971 |
| 0.8853567899249488 | 0.4615218340179602 | 0.0955531191689971 |
| 0.2186901232589849 | 0.6615218340169950 | 0.0955531191689971 |
| 0.5520234565920040 | 0.6615218340169950 | 0.0955531191689971 |
| 0.8853567899240151 | 0.6615218340169950 | 0.0955531191689971 |
| 0.2186901232599897 | 0.8615218340179431 | 0.0955531191689971 |
| 0.5520234565920161 | 0.8615218340179431 | 0.0955531191689971 |
| 0.8853567899250021 | 0.8615218340179431 | 0.0955531191689971 |
| 0.0648350526890062 | 0.1155063811579869 | 0.1115066246180021 |
| 0.3981683860230023 | 0.1155063811579869 | 0.1115066246180021 |
| 0.7315017193540072 | 0.1155063811579869 | 0.1115066246180021 |
| 0.0648350526889974 | 0.3155063811579679 | 0.1115066246180021 |
| 0.3981683860230064 | 0.3155063811579679 | 0.1115066246180021 |
| 0.7315017193550013 | 0.3155063811579679 | 0.1115066246180021 |
| 0.0648350526889927 | 0.5155063811589610 | 0.1115066246180021 |
| 0.3981683860230060 | 0.5155063811589610 | 0.1115066246180021 |

| | | |
|---|---|---|
| 0.7315017193559994 | 0.5155063811589610 | 0.1115066246180021 |
| 0.0648350526890109 | 0.7155063811579524 | 0.1115066246180021 |
| 0.3981683860230066 | 0.7155063811579524 | 0.1115066246180021 |
| 0.7315017193559543 | 0.7155063811579524 | 0.1115066246180021 |
| 0.0648350526889876 | 0.9155063811579294 | 0.1115066246180021 |
| 0.3981683860230023 | 0.9155063811579294 | 0.1115066246180021 |
| 0.7315017193560438 | 0.9155063811579294 | 0.1115066246180021 |
| 0.6307892314000183 | 0.5859748865998370 | 0.1920017484647686 |
| 0.9626085903686098 | 0.3849436259763598 | 0.1925441752208800 |
| 0.6302461161648711 | 0.9855765205714764 | 0.1928555917369169 |
| 0.6304394958861336 | 0.7849417903347568 | 0.1926201046932505 |
| 0.9635051916339307 | 0.7852393189667676 | 0.1928522351798913 |
| 0.9633253723667178 | 0.1853125500823997 | 0.1926257075544399 |
| 0.9634376192515107 | 0.5853526924347530 | 0.1926012733129634 |
| 0.9634316315515042 | 0.9850479509933354 | 0.1927813231345014 |
| 0.6304789167095194 | 0.1859324135545004 | 0.1942455046866401 |
| 0.2969300677302963 | 0.9852703354575690 | 0.1930093205263683 |
| 0.2969107725070684 | 0.5851775512441925 | 0.1929497904150939 |
| 0.2968848347644991 | 0.7850067067951729 | 0.1931029652149360 |
| 0.2967400103726546 | 0.1849620093744148 | 0.1927776384538970 |
| 0.6312350961428270 | 0.3855110943625008 | 0.1927089192474634 |
| 0.2972077272681072 | 0.3851584412332389 | 0.1937490385847976 |
| 0.8093560000439844 | 0.2392566389861299 | 0.2081977371968639 |
| 0.4766899705517855 | 0.8389770956647367 | 0.2081372415938782 |
| 0.8100640701035351 | 0.0384494926027408 | 0.2081675016897236 |
| 0.1434303675694782 | 0.8388700686042307 | 0.2079146101918818 |
| 0.1430964848488382 | 0.6381353375866704 | 0.2083828690625706 |
| 0.8100851211283401 | 0.8387257196942227 | 0.2082967569831654 |
| 0.1437804624464996 | 0.4383614175671658 | 0.2084180625917061 |
| 0.1432835605984795 | 0.0395402463031575 | 0.2083171573174787 |
| 0.8085607112071604 | 0.4375525067835533 | 0.2086849126054821 |
| 0.4759134563387723 | 0.6380600382930601 | 0.2080732203670061 |
| 0.4777545271224494 | 0.0399370944450823 | 0.2086746130212989 |
| 0.8095195717603061 | 0.6394427082093326 | 0.2081820358654070 |
| 0.1435745447664371 | 0.2392895973174583 | 0.2088014762734635 |
| 0.4764717423800117 | 0.2385367493525679 | 0.2086381157441312 |
| 0.4773816726198455 | 0.4393633343005001 | 0.2087095763510845 |
| 0.7049924444197674 | 0.1048774637149590 | 0.2854337915928196 |
| 0.3719439803947561 | 0.7088799913845368 | 0.2854717228991617 |
| 0.7028989318952766 | 0.3112209752131792 | 0.2902576817769206 |
| 0.0376401899751959 | 0.3068986103880112 | 0.2853222554378364 |
| 0.7061078592946715 | 0.9076681093018302 | 0.2856337990020100 |
| 0.3713590862542820 | 0.1052722877041082 | 0.2853947996316163 |
| 0.0380450873097756 | 0.5073103852870854 | 0.2857168691091507 |

| | | |
|---|---|---|
| 0.0386237232806629 | 0.7077203991787567 | 0.2857359702109150 |
| 0.7052124777527035 | 0.7083923033830118 | 0.2859960998156160 |
| 0.0388804568077965 | 0.9078103357913918 | 0.2857805616571504 |
| 0.3718905842734933 | 0.9079999669292043 | 0.2856853938760082 |
| 0.0378317024848926 | 0.1077330897881376 | 0.2859120548019840 |
| 0.7075428681659660 | 0.5116375376201627 | 0.2851427027878398 |
| 0.3696315972801546 | 0.5066777457940539 | 0.2887701653579572 |
| 0.3702712920759294 | 0.3063070421058163 | 0.2876630997388533 |
| 0.5543651492871795 | 0.7647389830580222 | 0.2985197563441069 |
| 0.8863797596798881 | 0.1619016598893079 | 0.2985868255478493 |
| 0.8875652242380017 | 0.5623738400032865 | 0.2988105900263194 |
| 0.5537317582933976 | 0.9603017293164273 | 0.2989549852553791 |
| 0.8868537023140488 | 0.7627569295767723 | 0.2993741068576572 |
| 0.2177183950747051 | 0.1576042527967953 | 0.2990028454581480 |
| 0.2178096732431659 | 0.5652461639764540 | 0.2990179577858831 |
| 0.8879437211186132 | 0.3624880537678116 | 0.2987541347207869 |
| 0.2201652933455377 | 0.7629502475059264 | 0.2991530748377023 |
| 0.2199627492798160 | 0.9609465952592566 | 0.2994121874104024 |
| 0.8870129589071853 | 0.9620642099357215 | 0.2993442724466234 |
| 0.2151059778014948 | 0.3603557297476218 | 0.2996658201026231 |
| 0.5524482269879811 | 0.5658782015929185 | 0.3013228823596475 |
| 0.5515613891894015 | 0.1602333376884775 | 0.3030853654260444 |
| 0.5498054356305103 | 0.3660615720478603 | 0.2991535315726693 |
